# Phenyl-Substituted Thiaboranes—Linked 2D and 3D Aromatics as Noncovalent Organic Framework Materials

**DOI:** 10.1021/acs.inorgchem.4c05457

**Published:** 2025-04-09

**Authors:** Jan Vrána, Josef Holub, Maksim A. Samsonov, Zdeňka Růžičková, Roman Bulánek, Jindřich Fanfrlík, Drahomír Hnyk, Rosa M. Gomila, Antonio Frontera, Aleš Růžička

**Affiliations:** † Department of General and Inorganic Chemistry, Faculty of Chemical Technology, University of Pardubice, Studentská 573, 532 10 Pardubice, Czech Republic; ‡ Institute of Inorganic Chemistry, Czech Academy of Sciences, Husinec-Řež 250 68, Czech Republic; § Department of Physical Chemistry, Faculty of Chemical Technology, University of Pardubice, Studentská 573, 532 10 Pardubice, Czech Republic; ∥ Institute of Organic Chemistry and Biochemistry of the Czech Academy of Sciences, Flemingovo náměstí 542/2, Praha 6 166 10, Czech Republic; ⊥ Departament de Química, Universitat de les Illes Balears, Crta de Valldemossa km 7.5, 07122 Palma de Mallorca (Baleares), Spain

## Abstract

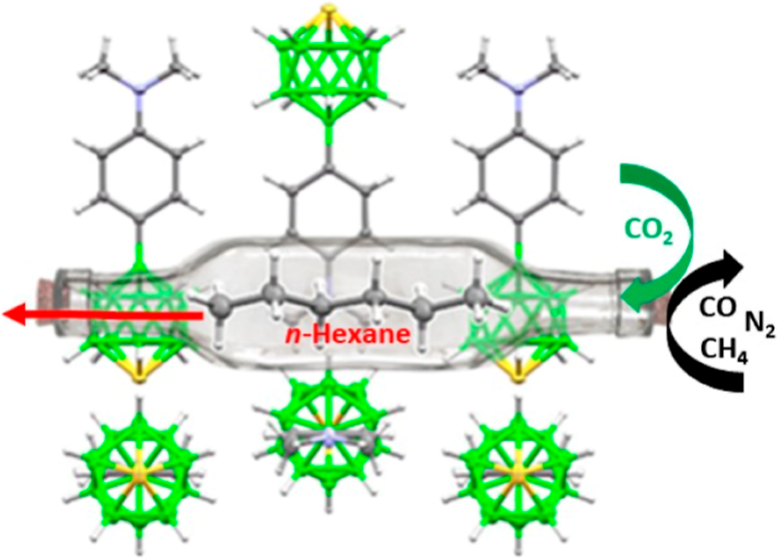

A series of 12-phenyl-*closo*-thiaboranes (12-(4-X-C_6_H_4_)-*closo*-1-SB_11_H_10_, where X = OMe (**2**), X = SMe (**3**), X = Ph (**4**), and X = NMe_2_ (**5**)) has been prepared. Except for **2**, all compounds exhibit a chalcogen bond of thiaborane to the phenyl ring or the neighboring molecule as major supramolecular structural motif. **5**, having the strongest (−12.47 kcal/mol) structure-making intermolecular interaction via noncovalent S···π(phenyl) chalcogen bond, was crystallized from different solvents in the form of various solvatopolymorphs. *n*-Hexane and diethyl ether can be removed from **5** easily upon the formation of a porous material with large cavities (up to 20.5% of the unit cell). This first stable and useful noncovalently bound organic framework material with an ultramicroporous structure exhibits a molecular sieve effect. The selective and repeatable adsorption of CO_2_ to the material crystallized from *n*-hexane was explained on the basis of cooperative and consecutive machine-like molecular interactions of quadrupolar CO_2_ molecule with B–H and amino groups inside rectangular cavities.

## Abstract 2

A porous material based on chalcogen bonds of phenyl-substituted thiaboranes was prepared by template-assisted crystallization. The material is sufficiently stable and able to absorb CO_2_ selectively and reversibly by interactions with borane core in a molecular sweep machine regime.

## Introduction

Traditional microporous materials with cavities of diameter < 2 nm, such as charcoal, ceramics, and zeolites,^1−6^ play key roles in a plethora of industrial applications such as adsorbents, heterogeneous catalysts, and molecular sieves as well as are means of everyday life. A relatively novel family of porous coordination polymers (PCPs)^7^ with a subclass of metal–organic frameworks (MOFs),^8,9^ porous organic frameworks (POFs),^10^ and covalent organic frameworks (COFs)^11−14^ has received much attention from scientists both in academia and industry. Several possible applications of these crystalline macromolecules arose in various physicochemical processes (filtration, gas separation, adsorption, extraction, cooling, etc.),^15−18^ heterogeneous catalysis,^19,20^ gas storage,^21^ carbon capture,^22−28^ the making of electronic devices,^29−32^ geology,^33^ environmental protection,^34,35^ and biology.^36,37^ As is clear from the common names of some of these materials, the subunits (metals and organic molecules in MOFs or purely organic molecules in COFs) are interconnected by very strong coordination or covalent bonds with dissociation energies of ∼20–150 kcal/mol.^38^ In zeolites, the situation is almost the same, except for the fact that the silicates or aluminosilicates are packed together by several Si–O, Al–O, and other element-oxygen bonds, thus forming networks based on connections of tetrahedral moieties derived from the orthosilicate structure. Considering the four-coordinate neighborhood of Si or Al atoms, more than 250 known natural or artificial zeolites, but several thousand MOFs and COFs, exist thanks to even higher structural variabilities and usually higher coordination numbers of metal atoms. Within these classes of compounds or supramolecular complexes, it is generally accepted that the strong bonds are structure-making and that the weak noncovalent interactions have only a weak effect and are usually responsible for ordered molecular motion or lamination/delamination processes.^3^

On the contrary, there is another group of molecular assemblies, so-called hydrogen-bonded organic frameworks (HOFs),^39−45^ where the aggregates were supposed to be too unstable with no effective permanent porosity because the strengths responsible for the molecular association are too weak (of about 10 kcal/mol only). Recently, some strategies have been developed for the preparation of these materials for various applications by the employment of oligo-carboxylic or sulfonic acids, heterocycles, or oligoamides. With respect to the mentioned families of compounds, the aggregation of molecules in order to create extrinsic porosity or cavities within the supramolecular architectures is not possible without a guest molecule holding the system together.

Combining the porosity and the specific movement of the organized molecules could provide access to molecular machines-like materials with specific functions such as directed motion or pumping the liquids by molecular propellers.^46−52^

The polyhedral boron compounds have valuable applications in several fields of related research such as medicine, drug delivery, or design of polymeric materials.^53−55^ In reticular chemistry, carboranes molecules are constructed and used as a spacer or scaffold mimicking thus organic groups (mainly *p*-substituted phenyl) for bridging metals in MOFs or reactive functional groups in COFs for almost 20 years. Concretely, these carborane porous materials^18,56−66^ MOFs (Zn, Co, Cu, Ln, and others) or COFs are robust and suitable to adsorb several gaseous or small molecules such as H_2_, CO_2_, methane, alkanes, or alcohols. In most of these materials, the main inner surface area of the cavities is composed not only of B–H but also C–H bonds, which exhibit slightly positive values of electrostatic potential. Robust material connected purely by noncovalent interactions and made dominantly by boron clusters has not been available up to now.

**Figure 1:**
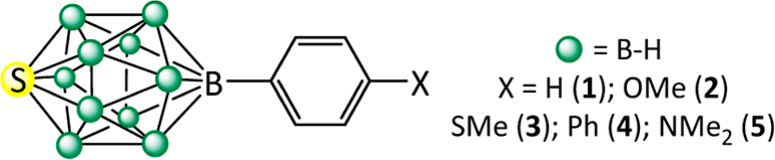
Numbering scheme of **1**–**5**.

Ten years ago, we started to investigate chalcogen bonding, made possible by the σ-hole localized on the sulfur atom and the π-system of phenyl-ring interaction, within the group of thiaboranes.^67^ This S···π chalcogen bond is a counterintuitive interaction considering that the S atom is covalently bound to electropositive B atoms. However, since the S atom is incorporated into the borane cluster via multicenter 2e-3c and 2e-4c chemical bonds,^68^ which do not conform to the classical electronegativity concept, there are areas of highly positive electrostatic potential (ESP) on the S atom that enable an attractive interaction with electron donors. The first derivative prepared, 12-phenyl-*closo*-1-SB_11_H_10_ (**1**), exhibits a relatively strong connection of such type (∼8.5 kcal/mol), which plays a dominant role in the type of crystal packing and is responsible for tight molecular aggregation (the sulfur-ring centroid distance of 3.24 Å).^53−55^ These molecules are air-stable and robust toward reactions with weak and medium-strong acids and bases.^69^ In the search for even more strongly interacting molecules, a series of 12-phenyl-*closo*-1-thiaboranes *para*-substituted by electron donating groups (12-(4-X-C_6_H_4_)-*closo*-1-SB_11_H_10_, where X = OMe (**2**), X = SMe (**3**), X = Ph (**4**), and X = NMe_2_ (**5**)) has been prepared (Figure 1).

## Results and Discussion

**Figure 2:**
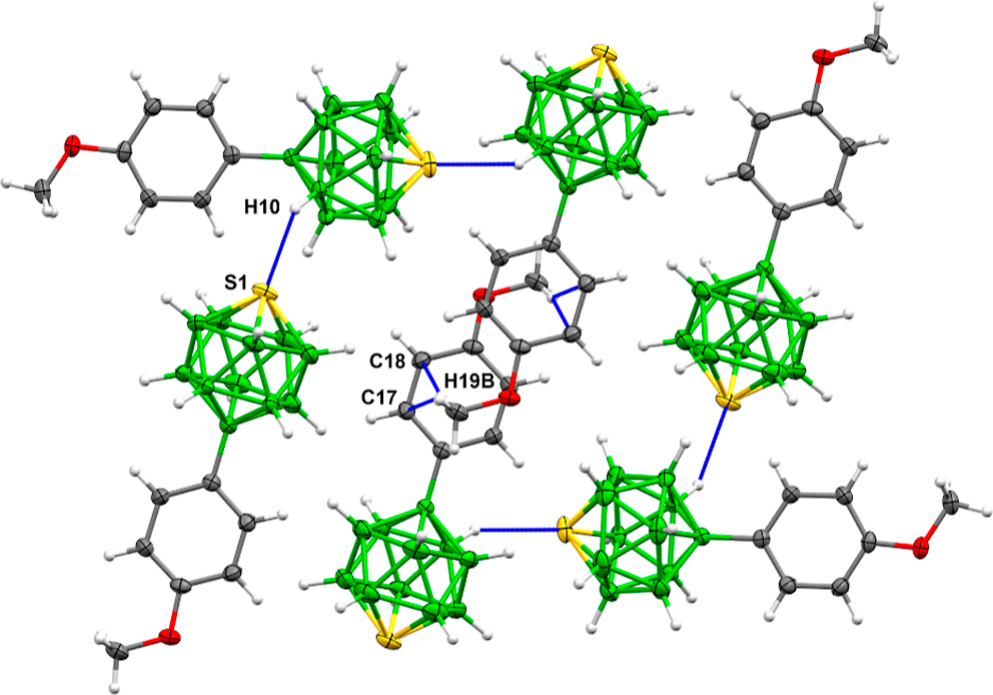
Detail of the supramolecular architecture of **2**.

First, the substitution of phenylthiaboranes by the most electron-donating group of the series—methoxy in **2**—dominates the course of chalcogen-bond formation by very strong nonclassical interactions of the oxygen atom with the B–H group of the thiaborane cage (Figures 2, S10, and S23). Weak interactions between B–H and sulfur or oxygen atoms, C–H···H–B, B–H···H–B, are responsible for the supramolecular architecture of **2**. In addition, very weak contacts attributed to a chalcogen bond between sulfur and oxygen atoms have also been detected. The separation of the sulfur atom and the aromatic-ring centroid is about 6 Å, and the axial vector of the molecule is almost parallel to the plane of the aromatic ring of the neighboring molecule; for the maximum interaction, however, they should be perpendicular. Moreover, the structure is also dominated by the parallel arrangement of the arenes.

**Figure 3:**
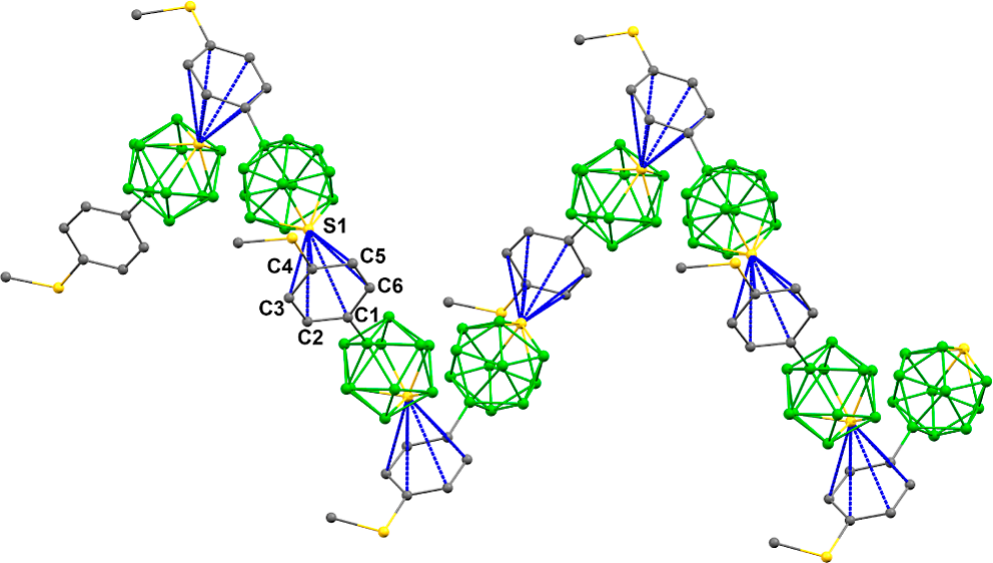
Detail of the supramolecular architecture of **3**; one spiral motif of the double-helical structure.

The obvious choice of a softer donor group has led us to the preparation of the SMe derivative (**3**). In the crystal structure of **3**, the chalcogen bond is described by the angle between the B12 (axial vertex), S1, and the phenyl-ring centroid of the neighboring molecule (C_g_) as well as by the separation of the sulfur and the aromatic-ring centroid, which seem to be the best descriptors, 160.3(3)° and 3.141(4) Å, respectively (Figure S11). These values are not so distant from the ideal straight angle, with the extreme value found for the palladium benzenethiolate complex (2.790 Å)^70^ or the only additional S···π contact below 3.15 Å (90% of ∑*r*_vdW_) found in the Cambridge Crystallographic Database [3.041 Å for the iron(II) isothiocyanate complex].^71^ In terms of the supramolecular architecture, the molecules form a double-helix structure (Figures 3 and S11).

**Figure 4:**
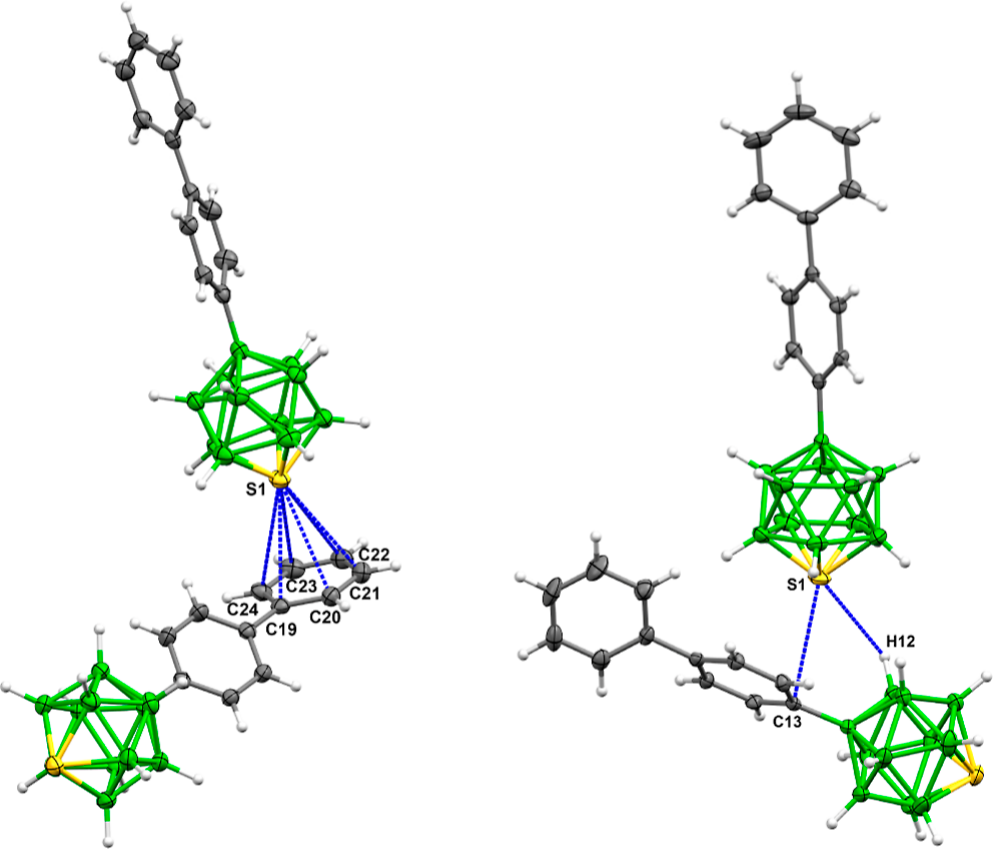
Details of the supramolecular architecture of **4h** (left) and **4b** (right).

The biphenyl derivative (**4**) seems to be an even weaker donor to the thiaborane cage. In general, however, there are two eligible phenyl rings with which the sulfur atom could interact. When **4** is crystallized from *n*-hexane (**4h**), the thiaborane interacts with the terminal phenyl ring of the neighboring molecule [Figures 4 (left) and S13], but the crystallization from benzene (**4b**) yields the structure depicted in Figures 4 (right) and S12, where the much weaker chalcogen bond to the bridging phenyl ring is pronounced. The explanation could be based on the nonexistent interaction of the solvent molecule and **4** in the case of hexane solution and thus the formation of an adduct that is sterically and thermodynamically more stable. Nevertheless, the benzene solvent interacts by π–π stacking preferably with the terminal phenyl ring, making space for a σ-hole interaction with the bridging phenyl only (for computational details, see Supporting Information, p. 34). Both structures, especially the mutual arrangement of the interacting phenyl ring and the neighboring thiaborane, are undoubtedly different (Figures S12 and S13). In the material crystallized from hexane, the molecules form stair-like architecture with the angle of the axial vectors of the neighboring molecules approaching the perpendicular arrangement and a relatively short distance between S and the centroid [B12–S1–C_g_ 165.7(2)° and S1–C_g_ 3.116(3) Å]. On the other hand, in the layered *zigzag* structure of **4b**, there are additional B–H···S contacts and the mentioned parameters are 132.4(3)° and 3.615(3) Å.

**Figure 5:**
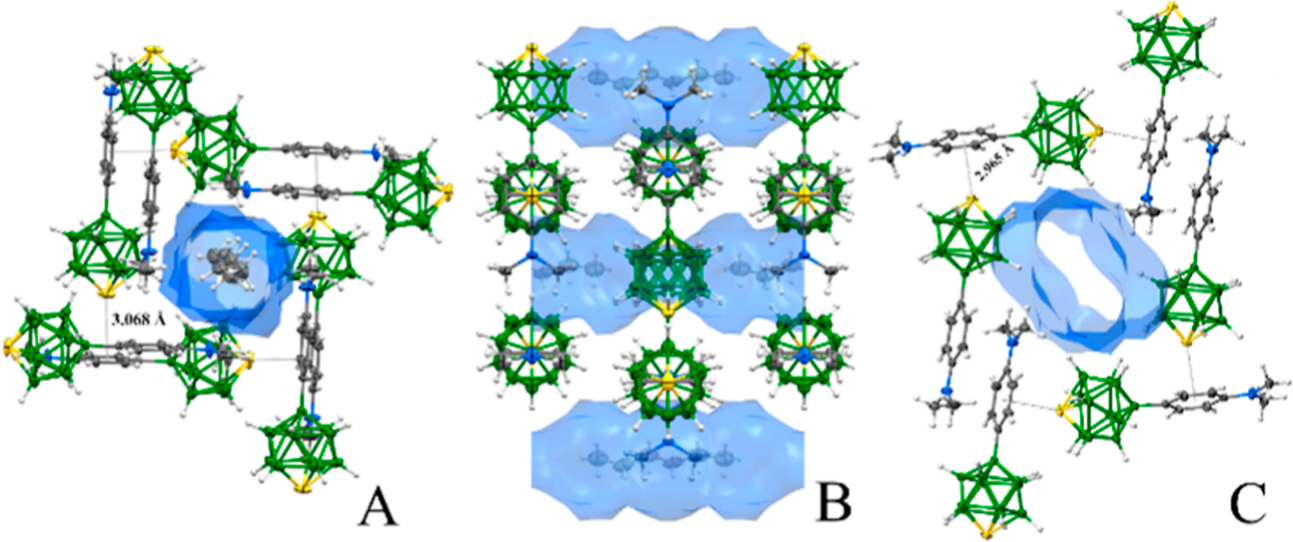
Details of the supramolecular architecture of **5**: (A) front view of a rectangular tunnel in the structure of **5** with the *n*-hexane molecule; σ-hole interactions are depicted with dotted lines; (B) side view of the tunnels in **5** with *n*-hexane molecules; and (C) front view of an elliptical tunnel in sublimed **5**.

Based on the experience with the concurrent interactions of **4** with benzene, **5** (NMe_2_ derivative) was crystallized from *n*-hexane. When the nicely shaped off-white crystals were removed from the mother liquor either under an argon atmosphere or in the air, an immediate color change to pale yellow was observed. The structure determined contains layers formed by tetragonal arrangements of four molecules of **5** held together by four very strong chalcogen bonds [Figures 5 and S20, 179.6(4)° and 3.065(3) Å] and C–H···π interactions. The layers are interconnected by many C–H···H–B dihydrogen-like bonds. It seems that the *n*-hexane molecules are accommodated inside the ‘tetragonal’ cavities created by chalcogen bonding.

**Figure 6:**
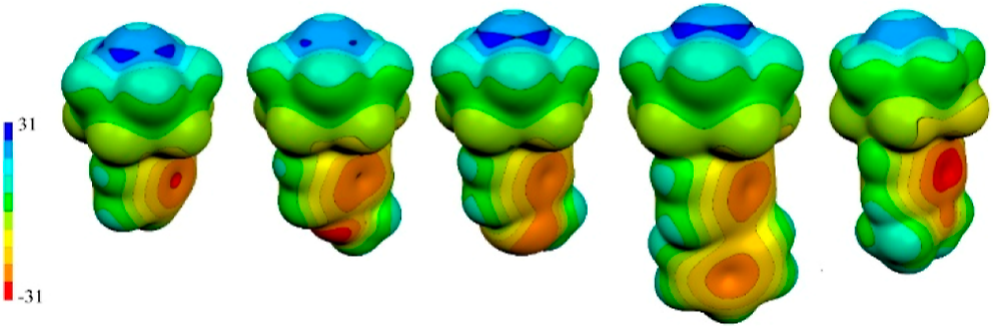
Computed ESP molecular surfaces of 12-(4-X-C_6_H_4_)-*closo*-1-SB_11_H_10_, where (from left to right) X = H (**1**), OMe (**2**), SMe, (**3**), Ph (**4**), and NMe_2_ (**5**). ESP color range is in kcal/mol.

The theoretical investigation started with the evaluation of the dipole moments of all molecules, where the expected modulation of dipole moments by *exo*-substitutions does not take place in **2**–**4** (∼3.6 D, Table S7), but the NMe_2_ group in **5** has significantly decreased the dipole moment value to 1.0 D. This can be explained by inducing an extra vector of the opposite direction with respect to that in *closo*-SB_11_H_11_. As a consequence, the sulfur atom in **5** exhibits the least positive σ-hole (*V*_S,max_ = 24.0 kcal/mol) of the whole series (Table S7 and Figures 6 and S25); however, it also has the most negative *V*_S,min_ (−29.0 kcal/mol) located on the phenyl ring. The SMe, OMe, and Ph groups do not change *V*_S,max_ (S) and *V*_S,min_ (ring) values considerably. In addition, the interaction energies are dependent not only on the phenyl substitution but also on the mutual orientation of both molecules, where the perpendicular one is the most attractive (Figure S24).

**Figure 7:**
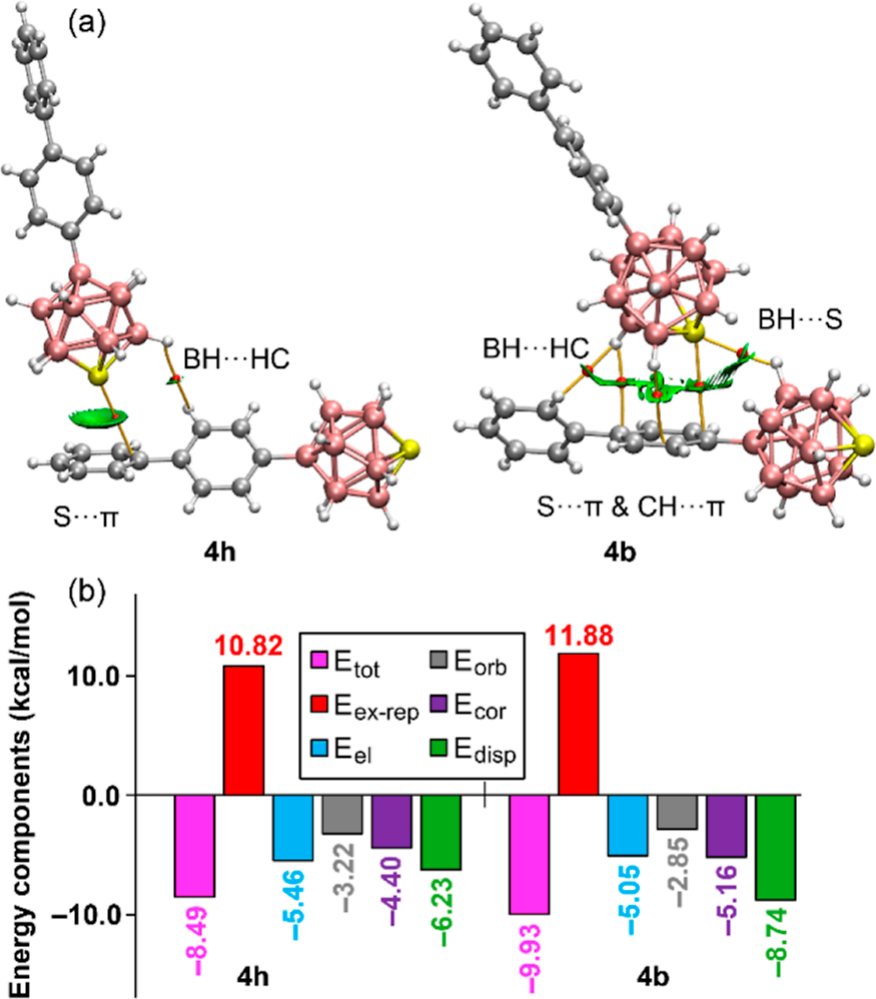
(a) QTAIM distribution of BCPs (red spheres) and bond paths (orange lines) and overlaid NCI plot RDG isosurfaces (ρ cutoff = 0.04 au, RDG = 0.5, scale ± 0.035 au). (b) Bar plot of the EDA analysis of the dimers of **4h** and **4b**. Level of theory: PBE0-D4/def2-TZVP.

More comprehensive DFT analyses were conducted to investigate the nature of S···π interactions described above, focusing on the binding modes observed in **4h** and **4b**, as illustrated in Figure 4. These dimers were examined using a combination of QTAIM (quantum theory of atoms in molecules)^72^ and NCI (noncovalent interaction)^73^ plot analyses, with results depicted in Figure 7. In **4h**, the distribution of bond critical points (BCPs, small red spheres) and bond paths (orange lines) is straightforward, featuring one BCP linking the S atom to a phenyl C atom and another connecting a BH bond of the borane to a CH bond of the central phenyl ring. The NCI plot describes better the S···π nature of the chalcogen bond, displaying a reduced density gradient (RDG) isosurface enveloping the entire π-system. The BH···HC contact appears to contribute minimally to stabilization, indicated by a small RDG isosurface and the positive nature of both connected H atoms. The QTAIM/NCI plot analysis of **4b** reveals the aromatic ring is linked to the thiaborane via three BCPs and bond paths that connect three C atoms to two H atoms of the thiaborane and the S atom. The RDG (reduced density gradient) isosurface’s shape between the S atom and the phenyl ring differs from that of **4h**, suggesting that the S···π interaction predominantly involves a single C atom of the ring. Additionally, like in **4h**, a BH···HC interaction characterized by a BCP, bond path, and a green RDG isosurface were observed. In addition, the combined QTAIM/NCI plot analysis also confirms the existence of a BH···S contact. The ESP surface of **4** shown in Figure 6 indicates that the five H atoms in alpha to the S atom on the thiaborane are electrophilic, while those in beta are nucleophilic, thus electrostatically favoring the BH···S contact observed in **4b**.

The interaction energies were explored using EDA (energy decomposition)^74^ analysis, as detailed in Figure 7b and Table S6. This analysis partitions the total energy (*E*_tot_, shown in magenta bars) into individual components: exchange-repulsion (*E*_ex-rel_, red bars), electrostatic (*E*_ele_, blue bars), orbital (*E*_orb_, gray bars), correlation (*E*_cor_, green bars), and dispersion (*E*_disp_, dark green bars). It was found that both binding modes have similar total energies, −8.49 kcal/mol for **4h** and −9.33 kcal/mol for **4b**, suggesting that the combination of S···π and CH···π interactions is favored over a more centered S···π interaction. Notably, the energy differences between the binding modes are not due to electrostatic forces or orbital interactions. Instead, correlation and particularly the dispersion contribution, which is 2.5 kcal/mol more favorable in **4b** than in **4h**, contribute significantly. This is likely due to the engagement of the π-system in three contacts in **4b** instead of one in **4h**.

The ESP surface of sulfur in compounds **1**–**5**, depicted in Figure 6, exhibits anisotropy with five σ-holes (ESP maxima) aligned along the B–S bonds, while the lone pair region shows a local ESP minimum. Contrary to expectations, the EDA of **4h** and **4b**, presented in Figure 7b, indicates that dispersion, rather than electrostatic forces, predominates in these homodimers. Thus, we wondered if this lone pair has some active role in the dimer formation. As example, the ELF (electron localization function)^75^ analysis computed for the homodimer of compound **3** illustrates the lone pair on the sulfur atom directed toward the aromatic ring (see Figure S40, SI). At this point, we decided to extend the DFT study to compounds **3** and **5**, using additional tools like NBO (natural bond orbitals)^76^ analysis for orbital donor–acceptor interactions and the comparative analysis of the electron density (ED) and electrostatic potential (ESP) minima along the bond path.

**Figure 8:**
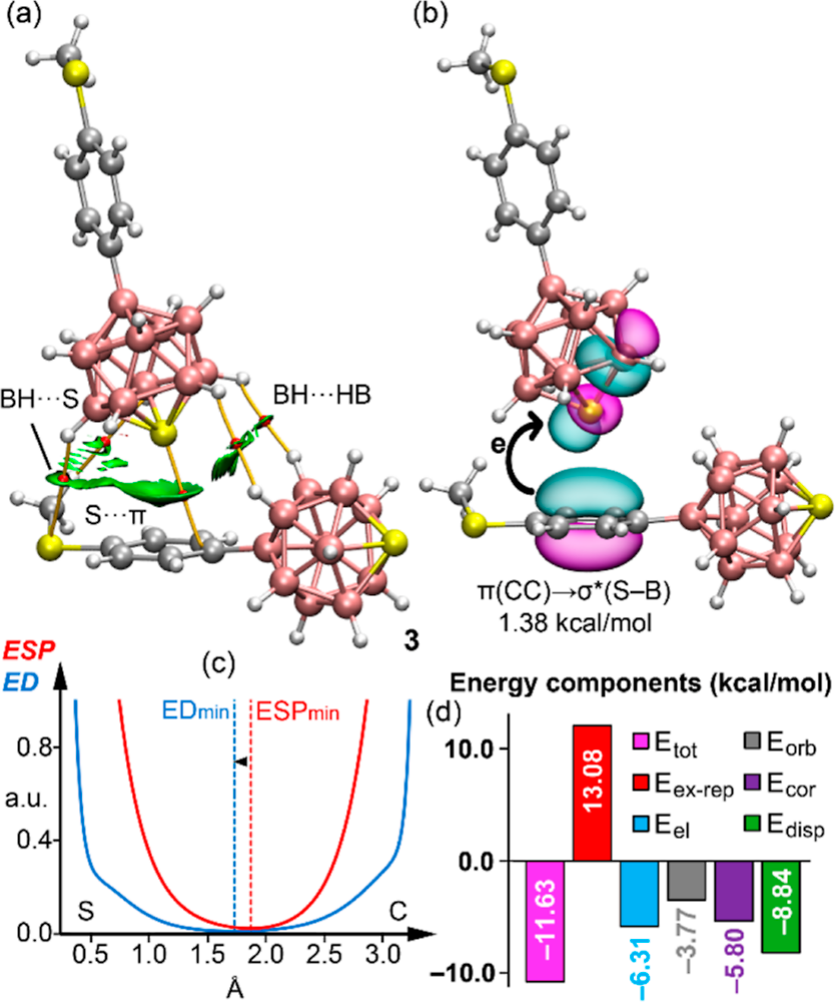
(a) QTAIM distribution of BCPs (red spheres) and bond paths (orange lines) and overlaid NCI plot RDG isosurfaces (ρ cutoff = 0.04 au, RDG = 0.5, scale ± 0.035 au). (b) Plot of the NBOs involved in the π(CC) → σ*(S–B) charge transfer, including the concomitant stabilization energy. (c) ED vs ESP plot along the path connecting the S to the C atom. (d) Bar plot of the EDA analysis of the dimer of **3**. Level of theory: PBE0-D4/def2-TZVP.

These tools help identify the electron donor and acceptor roles within noncovalent interactions. Notably, the ED minimum, aligning with the BCP, shifts toward the electron acceptor, while the ESP minimum shifts toward the electron donor. The QTAIM/NCI plot analysis of the dimer of compound **3** (Figure 8a) reveals a distribution similar to compound **4h** in terms of the S···π contact. Moreover, it highlights electrostatically favorable BH^δ+^···^δ−^HB interactions, alongside additional BH···S and CH···HB contacts. NBO analysis indicates the primary charge transfer from π-orbitals of the aromatic ring to the antibonding σ*(S–B) orbitals (*E*^(2)^ = 1.38 kcal/mol), without significant back-donation from sulfur’s lone pair to the aromatic ring (Figure 8b).

The corresponding ED vs ESP plot (Figure 8c) confirms sulfur’s electrophilic role, with the ED minimum nearer to the sulfur atom and the ESP minimum closer to the carbon atom. The EDA reveals that homodimerization in compound **3** is slightly more favorable than in compound **4**, primarily due to stronger dispersion forces and significant contributions from BH^δ+^···^δ−^HB and BH^δ+^···S interactions. These findings align with observations for compound **5**, as detailed in Figure S40, demonstrating similar interaction dynamics across these compounds.

The color changes and the unequal amount of *n*-hexane inside the cavities prompted us to remove the solvate completely. This can be achieved by gentle heating to 60–70 °C in the ultimate vacuum <10^–7^ mbar for 2 h, but more than 90% of hexane is already removed by treatment at 60–70 °C/1 mbar for 1 h. The He pycnometric density measurements of freshly crystallized (from *n*-hexane) material was performed to give the value of 1.082 g/cm^3^. After 1 h of vacuo treatment (2 mbar, 50 °C), the skeletal density increased by ca. 4.5% to 1.129 g/cm^3^, which corresponds roughly also the calculated pores volume (4.16%). The process is also corroborated by the TG measurement (Figure S37), which was conducted in the flow of dinitrogen. Surprisingly enough, there are hardly any structural changes of the thiaborane molecule and the supramolecular architecture after only partial or total removal of the *n*-hexane guest molecules. The square-shaped variable-size pores with a contact cross-section of 2.898–5.322 Å take about 11.5% (contact surface, probe radius 1.5 Å; solvent accessible surface of 4.3%, probe radius 1.0 Å) of the unit-cell volume, found by investigating the crystallographic data when the electron density is completely masked by the SQUEEZE procedure.^77^

Different guest molecules, including aliphatic and aromatic hydrocarbon solvents, ethers, carbon disulfide, and halogenated solvents, were tested to determine their suitability for the preparation of these assemblies, built purely by noncovalent interactions. The crystals obtained by vacuum sublimation again exhibit a structure (Figure 5C, Figure S22) characterized by the formation of layers through strong chalcogen bonding [164.1(4)° and 2.965(3) Å] and π–π stacking, which is probably responsible for lowering the symmetry from tetragonal, found for **5** grown from *n*-hexane, to orthorhombic. Several dihydrogen bonds form interlayer contacts like in the previous case. In contrast, these crystals grown without any structure-making guest include even larger cavities of the elliptical channels with major and minor ellipse axes of 8.338 and 5.648 Å, annexing about 20.5% of the unit-cell volume. The crystal structure obtained from the diethyl ether solution is exactly the same, with one molecule of solvent per two thiaboranes inside the channels, from which it immediately escapes after crystals are removed from the solution or given to *vacuo*. The crystal structures obtained from aromatic solvents, such as benzene, toluene, mesitylene or hexafluorobenzene, CS_2_, decalin, and CCl_4_, strongly depend on the guest molecule present in the crystal lattice (Figures S14–S22), but the most consolidating element of them all is the presence of the guest molecule interacting via dihydrogen bonding or π–π stacking with the assembly of the hosts. These interactions, which are not present in **5h** at all and only to a limited degree in **5**·(Et_2_O)_0.5_, are most probably responsible for the fact that other guest molecules than *n*-hexane and diethyl ether could not be removed from the cavities without structural changes (see the cavities in Figures S14–S22).

In order to shed more light on this phenomenon, the samples of **5** crystallized from *n*-hexane (**5h**) and diethyl ether (**5e**) were investigated using similar approaches, methods, and tools as those used for the characterization of MOFs or COFs.^7−13^ Textural properties (specific surface area and porosity) were investigated by the physisorption of dinitrogen at the temperature of liquid nitrogen (Figure S31). Sample **5h** degassed at room temperature exhibits very low adsorption capability, and the isotherm resembles an adsorption isotherm of type III (according to IUPAC classification). The specific surface area was estimated to be as small as 0.5 m^2^/g (B.E.T.). This indicates that the material is absolutely nonporous and has large particles or that the pores are still blocked by the solvent, which cannot be removed by outgassing at room temperature. In order to verify this, the sample was degassed at the elevated temperature of 70 °C (**5h**_**70**_). The amount adsorbed increased significantly. The shape of the isotherm changed to type II, characteristic of multilayer adsorption on nonporous or macroporous material. The specific surface area of **5h**_**70**_ increased to 16.6 m^2^/g. However, the isotherm shows neither the presence of micropores (steep initial increase or intercept on the *y*-axis) nor mesopores for which a step associated with capillary condensation and a hysteresis loop is characteristic. Thus, it is clear that for dinitrogen, the material is still nonporous (due to diffusion limitations or molecular sieve effect) and the increase in specific area is due to a change in particles morphology or surface properties (wetting for liquid nitrogen) by removing *n*-hexane, which would correspond to a change in isotherm character from type III to type II according to IUPAC. Sample **5e** exhibited a specific surface area of 11.5 m^2^/g even after outgassing at room temperature due to the easier evaporation of the solvent from the sample. It should be noted here that all experimental studies of textural properties and CO_2_ adsorption for all materials were carried out on a single crystalline material (evaluated prior to and after the experiments by sc-XRD techniques) of approximate dimensions 0.2 mm × 0.2 mm × 0.1 mm, which is far from the studies made on common microcrystalline material. From another point of view, the prepared material should differ significantly from characteristics of known materials such as zeolites, MOFs, COFs, and HOFs, where the accommodation of dinitrogen molecules, covered by negative electrostatic potential, is much easier due to predominant interactions with rather positively charged metal centers’, C–H, O–H or N–H bonds. In the case of novel borane-based materials, the polarity of the surface B–H bonds is opposite, and in addition, the driving force for the dinitrogen permeation into **5** are only interactions with C–H groups of the phenyl and methyl groups, which are again very close to hydridic B–H and amino groups. This structural discrepancy is most probably the reason why the classical measurement and concepts of textural properties of this type of material, together with narrow cavities, give much lower values than would be expected for classical materials. Another supportive proof is the easy accommodation and cocrystallization from various hydrocarbon solvents (see above), where the principally positively charged surface of C–H bonds is prone to interact with terminal B–H bonds on the surface of the molecules making the cavities.

**Figure 9:**
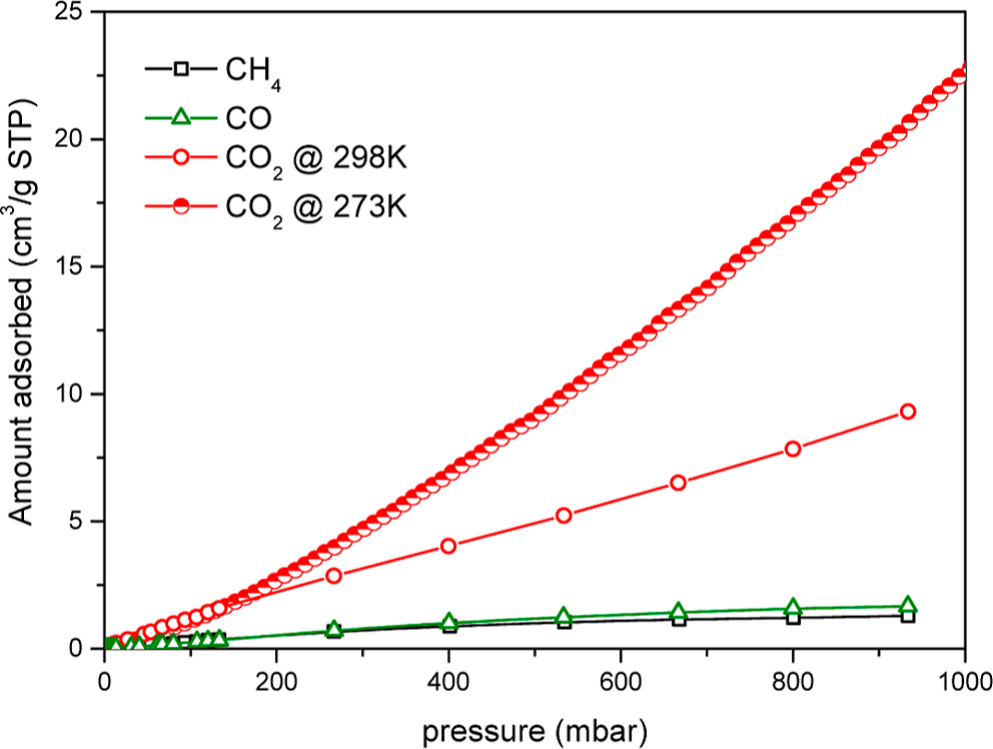
Adsorption isotherms of methane, CO, and CO_2_ at 298 K (open symbols) and CO_2_ at 273 K (half-up symbols) on the **5h**_70_ sample.

One of the central topics of current interest, with the aim of reducing the greenhouse effect, is the capture of carbon in the form of CO_2_. Samples **5h**_**70**_ and **5e** were subjected to the adsorption of CO_2_ at 273 K [23.8 cm^3^/g STP (1.062 mmol of CO_2_/3.559 mmol **5h**_**70**_)—see Figure 9]. The characteristics of both samples, such as the specific surface area (309 and 105 m^2^/g for **5h**_**70**_ and **5e**, respectively) and the size of the micropores (0.6–0.8 nm for **5h**_**70**_ and 0.45–0.6 nm for **5e**), had been derived from CO_2_ adsorption isotherms by application of the NL-DFT approach for calculation. It should be noted that the model developed to describe the adsorption of CO_2_ on carbon-based materials was used to evaluate the adsorption data, which does not correspond exactly to the real system but is still the closest of the developed models. The discrepancy between N_2_- and CO_2_-probed surface areas can be caused by the differences in the kinetic diameters of both molecules (CO_2_ is smaller) and the diffusivity of molecules into pores, related to the electronic character of the molecules (CO_2_ is a quadrupole). Based on this information and facts about the electrostatic potential of the molecular surface of **5**, one can expect the cooperative interaction of terminal B–H and amino moieties with the carbon atom and methyl or phenyl C–H moieties with the oxygen atoms of the CO_2_ molecules (see Supporting Information and below for theoretical models and interaction energies).

In order to gain more insight into the adsorption properties of the **5h**_**70**_ sample, which has the largest surface area, the adsorption of the other gases, differing in size and polarity (like methane, carbon monoxide, and carbon dioxide), at 298 K was investigated (Figure 9). It is evident that the adsorption of CO_2_ is remarkably higher when compared to that of the other two gases. This indicates the possibility to separate CO_2_ from methane or CO with relatively high selectivity. It is necessary to note that the adsorption capacity of this material is significantly lower than that of the other adsorbents, such as zeolites or some MOFs.^26,78,79^ On the other hand, this drawback is compensated by the fact that the maximum possible ratio of CO_2_ adsorbed to **5h** is equimolar (the mass fraction of 0.156), where one-third of the value is reached under ambient conditions, and thus comparable to the most promising materials. Unfortunately, the bespoke CO_2_ adsorption in **5h**_**70**_ proven by physicochemical methods and color changes (Figure S34) is not solidly supported by changes in powder XRD and Raman spectra (Figures S35 and S36), which differ only negligibly.

**Figure 10:**
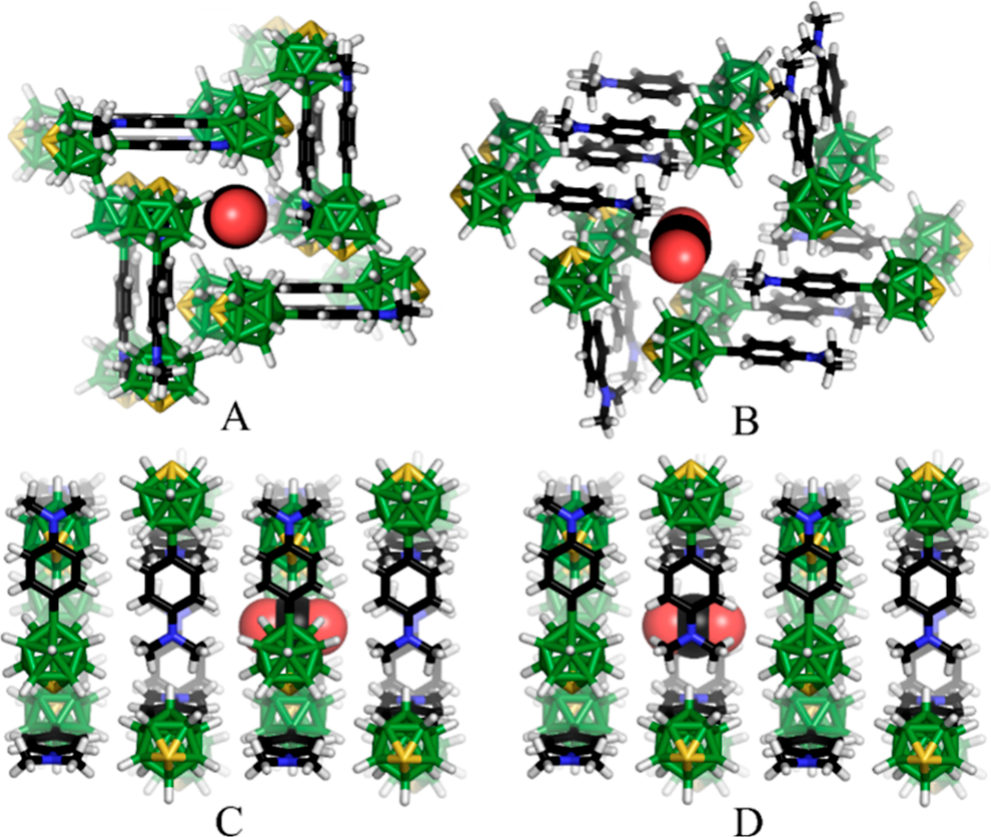
Models of the CO_2_ molecules adsorbed into the cavities of the crystallographically determined structures of **5**. (A) Front view of a rectangular tunnel in **5h**_**70**_, interacting with four NMe_2_ groups; (B) front view of an elliptical tunnel in **5sub** or **5e**, interacting with two thiaborane groups, *E*_int_ = −5.6 kcal/mol; (C) side view of a rectangular tunnel in **5h**_**70**_, interacting with four thiaborane groups, *E*_int_ = −6.1 kcal/mol; and (D) side view of a rectangular tunnel in **5h**_**70**_, interacting with four NMe_2_ groups, *E*_int_ = −9.2 kcal/mol.

The counterintuitive adsorption properties of **5h**_**70**_ and **5e** samples (**5e** has larger cavities but adsorbs less CO_2_) led us to calculate the interaction energies between the CO_2_ molecule and the molecules forming the cavity. Based on our calculations and crystallographic results, the straight tunnels in **5h** are made of alternating layers, which form cavities with nonuniform rectangular cross sections slued by ca. 30° (Figure S26). The first type of cavity is made of four thiaboranes, which allows an interaction between many negative B–H groups and the positive part of CO_2_ (*d* = 5.58 Å, *E*_int_ = −6.1 kcal/mol) (Figure 10A). In the second type of cavity, CO_2_ interacts with four amino groups (*d* = 7.64 Å, *E*_int_ = −9.2 kcal/mol) (Figure 10B) like in the case of the liquid amines used for CO_2_ capture and storage.^80−82^ All together, these rectangular cavities work as cooperative molecular machines of the proper type, size, and shape, where the CO_2_ molecules move through the tunnel through the rotation of thiaboranes and amino groups; once the CO_2_ interacts with nitrogen, it is pushed forward by the repulsion of oxygen and B–H groups. The same is valid for the carbon from the CO_2_ interaction with B–H groups, where the repulsion of oxygen and amino groups is the driving force of the motion. On the contrary, only a part of the elliptical shape of the larger cavity in **5e** is able to interact with CO_2_ by two thiaboranes (Figure 10C) at the same time, only leading to the *E*_int_ of −5.6 kcal/mol, instead of four thiaboranes or amines in the case of **5h** (Figure 10D).

## Conclusions

We have prepared the first stable porous materials purely based on the chalcogen bonding of phenyl-substituted thiaboranes. Insights into the physical nature of S···π chalcogen bonding were obtained through DFT calculations and various computational tools, demonstrating that dispersion forces are the dominant contributors, surpassing electrostatic effects. The properties of this porous framework differ from conventional porous materials such as MOFs, COFs, HOFs, and materials with intrinsic microporosity^83^ or based on the chalcogen-bonded organic framework^84^ because its inner cavity/tunnel surface hydrogen atoms of B–H bonds are negatively charged in comparison to the C–H bond surface of these materials. Thus, it is able to accommodate a significant quantity of CO_2_ selectively and reversibly. Cooperative treatment of CO_2_ interacting with different parts of thiaborane molecules as a molecular machine (Movie S1) could establish a new area of stable and useful noncovalent organic framework materials with tunable adsorption properties by opposite surface polarity and specific pore geometry driven by collaborative interactions with moving parts of the framework.

## Experimental Section

### Synthesis

All manipulations were carried out under an argon atmosphere using the standard Schlenk tube technique. Solvents were dried using Pure Solv-Innovative Technology equipment under an argon gas atmosphere. The starting compound 12-I-*closo*-1-SB_11_H_10_ was prepared according to the published procedure.^85^ Other compounds were purchased and used without further purification. Elemental analyses were performed on an LECO-CHNS-932 analyzer.

Compounds **2**–**5** were prepared by modified Negishi coupling of iodinated thiaborane 12-I-*closo*-1-SB_11_H_10_ with the corresponding organozinc reagent (4-X-C_6_H_4_–ZnBr, where X = OMe, SMe, Ph, or NMe_2_) catalyzed by PdCl_2_(PPh_3_)_2_ (Figure S1). Though harsh reaction conditions (high loading of catalyst, 4 equiv of the organozinc reagent, and heating to reflux) were needed in order to prepare desired adducts, all compounds were isolated in moderate (40% for **5**) to high isolated yields (80–85% for **2**–**4**). All compounds were characterized in solution by multinuclear NMR spectroscopy. ^1^H and ^13^C NMR spectra revealed one set of expected signals for the organic moieties. ^11^B NMR spectra exhibited an identical pattern of 1:5:5 with the signal of the B12 boron atom being shifted upfield compared to the parent thiaborane (Δδ(^11^B) ∼ 27.5 ppm).

### NMR Spectroscopy

^1^H, ^11^B, and ^13^C NMR spectra were recorded on Bruker Avance 500 MHz spectrometer or Bruker Ultrashield 400 MHz, using a 5 mm tunable broad-band probe. Appropriate chemical shifts in ^1^H and ^13^C NMR spectra were related to the residual signals of C_6_D_6_: δ(^1^H) = 7.16 ppm and δ(^13^C) = 128.39 ppm. ^11^B chemical shifts were related to external standard BF_3_•OEt_2_ [δ(^11^B) = 0.0 ppm].

### sc-XRD

Full sets of diffraction data (Tables S2–S5) for **2**–**5cl** were collected at 150(2)K with a Bruker D8-Venture diffractometer equipped with Cu (Cu Kα radiation; λ = 1.54178 Å for **4h**, **5f**, 5m) or Mo (Mo Kα radiation; λ = 0.71073 Å rest of the compounds) microfocus X-ray (IμS) sources, Photon CMOS detector, and an Oxford Cryosystems cooling device.

The frames were integrated with the Bruker SAINT software package using a narrow frame algorithm. Data were corrected for absorption effects using the Multi-Scan method (SADABS). Obtained data were treated by XT-version 2014/5 and SHELXL-2017/1 software^86^ implemented in the APEX3 v2016.9-0 (Bruker AXS) system.^87^

Hydrogen atoms were mostly localized on a difference Fourier map; however, to ensure uniformity of treatment of crystal, all hydrogens were recalculated into idealized positions (riding model) and assigned temperature factors *H*_iso_(H) = 1.2 U_eq_ (pivot atom) or of 1.5 U_eq_ (methyl).

Minor disorders of carborane cages or solvent molecules in **3**, **5h**, **5b**, **5f**, **5e**, **5cs**, **5d**, and **5cl** were treated by standard methods. In **5cl**, data completeness (0.904) value is the reason for an A-alert during the PLANTON checkCIF procedure. The model, Fourier map, thermal ellipsoid of heavier elements, as well as the uniformity of the structure refinement enables its publication.

Crystallographic data for structural analysis has been deposited with the Cambridge Crystallographic Data Centre, CCDC nos. 2068574–2068586. Copies of this information may be obtained free of charge from The Director, CCDC, 12 Union Road, Cambridge CB2 1EY, UK (fax: + 44–1223–336033; e-mail: deposit@ccdc.cam.ac.uk or www: http://www.ccdc.cam.ac.uk).

### Computational Details

The molecular ESP surfaces were computed on the 0.001 au molecular surfaces at the HF/cc-pVDZ level using the Gaussian09^88^ and Molekel4.3^89,90^ programs.

The chalcogen bonding crystallographic motifs were examined by the symmetry-adapted perturbation-theory (SAPT) methodology, which enables decomposition of the interaction energies. We employed the simplest truncation of SAPT (SAPT0) decomposition^91^ in combination with the recommended jun-cc-pVDZ basis sets.^92^ H and B atoms were optimized at the RI-DFT-D3/DBLYP/DZVP^93^ level while the remaining atoms were kept fixed in crystal positions.

The binding of CO_2_ to **5** was evaluated at the RI-DFT-D3/DBLYP/DZVP^92^ level. The model of the cavity consisted of 16 molecules of **5** and a single CO_2_ (over 500 atoms). The Turbomole (7.0),^94^ P_SI_4,^95^ and Cuby4^96^ program packages were used. Movie S1 was prepared using Amber14,^97^ Cuby4,^96^ and Chimera 1.10.2^98^ programs. The GAFF force field was employed.^99^ The missing parameters for boron atoms were transferred from UFF.^100^

The QTAIM,^101^ EDA (Kitaura-Morokuma),^102^ ELF,^103^ ED vs ESP and NCIplot^104^ calculations were performed at the PBE0-D4/def2-TZVP level of theory^105−108^ since it has proven adequate for the evaluation of chalcogen-bonding interactions,^109,110^ by means of the Turbomole 7.0 software.^94^ The QTAIM and NCIPlot analyses were represented using the VMD software.^111^ The Multiwfn program^112^ was used for the QTAIM and NCIplot calculations. The following settings were used to represent the NCIplot in the figures of this manuscript: RDG = 0.5, ρ cutoff = 0.04 au color code −0.035 au ≤ (signaλ2)ρ ≤ 0.035 au The natural bond orbital (NBO) analysis^113^ was performed using the NBO7 program.^114^

### Adsorption Isotherm Measurements

The adsorption isotherms were collected using a Micromeritics ASAP 2020 automatic gas sorption analyzer equipped with oil-free turbomolecular vacuum pumps (ultimate vacuum <10^–7^ mbar) and valves, guaranteeing contamination-free measurements. All used gases (He, N_2_, H_2_, CO_2_, CH_4_) were of ultrahigh purity (UHP, grade 5.0, 99.999% or better), and the STP volumes are given according to the NIST standards (293.15 K, 101.325 kPa). Helium (99.9999%) was used for the determination of the free spaces of the sample tubes. H_2_ and N_2_ adsorption isotherms were measured at 77 K (liquid nitrogen bath), whereas CO_2_, CO, and CH_4_ adsorption isotherms were measured at temperatures ranging from 273 to 298 K, controlled by a high-precision custom-made cryostat (E-lab services, Czech Republic). Prior to adsorption measurements, the samples were degassed for 2 h under a vacuum generated by the turbomolecular pump at room temperature (samples denoted—RT) or at 70 °C (samples denoted—70C). Adsorption data was evaluated by MicroActive data reduction software (v4.2, Micromeritics, USA). The specific surface area was evaluated from nitrogen adsorption isotherms by B.E.T. theory applied to the data in an interval of relative pressures satisfying the condition of the positive derivation of the data in the so-called Rouquerol plot [*n*_ads_(1 – *P*/*P*_0_) vs *P*/*P*_0_ dependence]. CO_2_ adsorption isotherms measured at 273 K were subjected to pore size distribution by applying the NL DFT approach using the relevant kernel (CO2@273-Carbon Slit Pores 10 atm).

### Thermogravimetry

TG experiment was measured on a NETZSCH STA 449F5 Jupiter TG-DSC instrument. The mass of the **5h** sample freshly taken from the *n*-hexane mother liquor was 3.65 mg.

## Supporting Information Available

Materials and methods as well as additional text and figures, synthesis, NMR spectra for all compounds, XRD parameters and data, TGA, adsorption data, microscopy, and computational details (PDF)
Cooperative treatment of CO_2_ interacting with different parts of thiaborane molecules as a molecular machine (MP4)
