# Supplementary material for: Phenyl-Substituted Thiaboranes—Linked 2D and 3D Aromatics as Noncovalent Organic Framework Materials
Source: Inorg Chem. 2025 Apr 9;64(15):7377–87. doi: 10.1021/acs.inorgchem.4c05457 (PMC12015963; doi:10.1021/acs.inorgchem.4c05457)
Supplement: Supplementary file 1 — ic4c05457_si_001.pdf [file ic4c05457_si_001.pdf]

Supporting Information

Phenyl-Substituted Thiaboranes – linked 2D and 3D Aromatics as Non-Covalent Organic Framework Materials

Jan Vrána,[‡] Josef Holub,[∆] Maksim A. Samsonov,[‡] Zdeňka Růžičková,[‡] Roman Bulánek,[†] Jindřich Fanfrlík,[◊] Drahomír Hnyk,[∆] Rosa M. Gomila,[§] Antonio Frontera[§] and Aleš Růžička*[‡]

[‡]Department of General and Inorganic Chemistry, Faculty of Chemical Technology, University of Pardubice, Studentská 573, 532 10 Pardubice, Czech Republic. Correspondence to: ales.ruzicka@upce.cz
[∆]Institute of Inorganic Chemistry, Czech Academy of Sciences, 250 68 Husinec-Řež, Czech Republic
[†]Department of Physical Chemistry, Faculty of Chemical Technology, University of Pardubice, Studentská 573, 532 10 Pardubice, Czech Republic.
[◊]Institute of Organic Chemistry and Biochemistry of the Czech Academy of Sciences, Flemingovo náměstí 542/2, 166 10 Praha 6, Czech Republic
[§]Departament de Química, Universitat de les Illes Balears, Crta de Valldemossa km 7.5, 07122 Palma de Mallorca (Baleares), SPAIN.

Table of Contents

Materials and Methods......S2
Synthesis......S2
NMR spectroscopy......S2
sc-XRD......S2
Computational details......S2
Adsorption isotherm measurements S3
Thermogravimetry......S3
Supplementary Text......S4
Synthesis......S4
Crystallographic section......S16
Binding modes in X-ray crystal structures......S34
Dipole moments and electrostatic potential (ESP) molecular surfaces......S37
Interactions with CO_2......S38
Adsorption data......S42
Optical Microscopy......S47
PXRD......S48
Raman Spectroscopy......S49
Thermogravimetry......S50
References......S51

Materials and Methods

Synthesis

All manipulations were carried out under an argon atmosphere using the standard Schlenk tube technique. Solvents were dried using Pure Solv-Innovative Technology equipment under an argon gas atmosphere. The starting compound 12-I-*closo*-1-$SB_{11}H_{10}$ was prepared according to the published procedure[1]. Other compounds were purchased and used without further purification. Elemental analyses were performed on an LECO-CHNS-932 analyzer.

NMR spectroscopy

^{1}H, ^{11}B, and ^{13}C NMR spectra were recorded on Bruker Avance 500 MHz spectrometer or Bruker Ultrashield 400 MHz, using a 5 mm tuneable broad-band probe. Appropriate chemical shifts in ^{1}H and ^{13}C NMR spectra were related to the residual signals of C_6D_6: $\delta(^{1}H) = 7.16$ ppm and $\delta(^{13}C) = 128.39$ ppm. ^{11}B chemical shifts were related to external standard BF_3•OEt_2 ($\delta(^{11}B) = 0.0$ ppm).

sc-XRD

Full-sets of diffraction data (Tables S2–S5) for **2**–**5cl** were collected at 150(2)K with a Bruker D8-Venture diffractometer equipped with Cu (Cu/Kα radiation; λ = 1.54178 Å for **4h**, **5f**, **5m**) or Mo (Mo/Kα radiation; λ = 0.71073 Å rest of the compounds) microfocus X-ray (IµS) sources, Photon CMOS detector and Oxford Cryosystems cooling device was used for data collection.

The frames were integrated with the Bruker SAINT software package using a narrow frame algorithm. Data were corrected for absorption effects using the Multi-Scan method (SADABS). Obtained data were treated by XT-version 2014/5 and SHELXL-2017/1 software[2] implemented in APEX3 v2016.9-0 (Bruker AXS) system[3].

Hydrogen atoms were mostly localized on a difference Fourier map, however to ensure uniformity of treatment of crystal, all hydrogens were recalculated into idealized positions (riding model) and assigned temperature factors Hiso(H) = 1.2 Ueq (pivot atom) or of 1.5 Ueq (methyl).

Minor disorders of carborane cages or solvent molecules in **3**, **5h**, **5b**, **5f**, **5e**, **5cs**, **5d**, **5cl** were treated by standard methods. In **5cl**, data completeness (0.904) value is the reason for an A-alert during Platon checkcif procedure. The model, Fourier map, thermal ellipsoid of heavier elements, as well as the uniformity of the structure refinement enables its publication.

$R_{int} = \sum|F_o^2 - F_{o,mean}^2|/\sum F_o^2$, GOF $= [\sum(w(F_o^2 - F_c^2)^2)/(N_{diffrs} - N_{params})]^{1/2}$ for all data, $R(F) = \sum||F_o| - |F_c||/\sum|F_o|$ for observed data, $wR(F^2) = [\sum(w(F_o^2 - F_c^2)^2)/(\sum w(F_o^2)^2)]^{1/2}$ for all data.

Crystallographic data for structural analysis has been deposited with the Cambridge Crystallographic Data Centre, CCDC nos. 2068574–2068586. Copies of this information may be obtained free of charge from The Director, CCDC, 12 Union Road, Cambridge CB2 1EY, UK (fax: +44-1223-336033; e-mail: deposit@ccdc.cam.ac.uk or www: http://www.ccdc.cam.ac.uk).

Computational details

The molecular ESP surfaces were computed on the 0.001 a.u. molecular surfaces at the HF/cc-pVDZ level using the Gaussian09[4] and Molekel4.3[5, 6] programs.

The chalcogen bonding crystallographic motifs were examined by the symmetry-adapted perturbation-theory (SAPT) methodology, which enables decomposition of the interaction energies. We employed the simplest truncation of SAPT (SAPT0) decomposition[7] in combination with the recommended jun-cc-pVDZ basis sets[8]. H and B atoms were optimized at the RI-DFT-D3/DBLYP/DZVP[9] level while the remaining atoms were kept fixed in crystal positions.

The binding of CO_2 to **5** was evaluated at the RI-DFT-D3/DBLYP/DZVP[8] level. The model of the cavity consisted of 16 molecules of **5** and a single CO_2 (over 500 atoms). The Turbomole (7.0)[10], $P_{SI}4$[11], and Cuby4[12] program packages were used. The movie S1 was prepared using Amber14[13], Cuby4[12], and Chimera 1.10.2[14] programs. The GAFF force field was employed[15]. The missing parameters for boron atoms were transferred from UFF[16].

The QTAIM,[17] EDA (Kitaura-Morokuma),[18] ELF,[19] ED vs ESP and NCIplot[20] calculations were performed at the PBE0-D4/def2-TZVP level of theory[21-24] since it has proven adequate for the evaluation of chalcogen bonding interactions,[25-26] by means of the Turbomole 7.7 software.[10] The QTAIM and NCIPlot analyses were represented using the VMD software.[27] The Multiwfn program[28] was used for the QTAIM and NCIplot calculations. The following settings were used to represent the NCIplot in the Figures of this manuscript: RDG = 0.5, ρ cut-off = 0.04 a.u. color code –0.035 a.u. ≤ $(\text{signa}\lambda_2)\rho$ ≤ 0.035 a.u. The natural bond orbital (NBO) analysis[29] was performed using the NBO7 program.[30]

Adsorption isotherm measurements

The adsorption isotherms were collected using a Micromeritics ASAP 2020 automatic gas sorption analyzer equipped with oil-free turbomolecular vacuum pumps (ultimate vacuum $<10^{-7}$ mbar) and valves, guaranteeing contamination-free measurements. All used gases (He, N_2, H_2, CO_2, CH_4) were of ultra-high purity (UHP, grade 5.0, 99.999% or better) and the STP volumes are given according to the NIST standards (293.15 K, 101.325 kPa). Helium (99.9999%) was used for the determination of the free spaces of the sample tubes. H_2 and N_2 adsorption isotherms were measured at 77 K (liquid nitrogen bath), whereas CO_2, CO and CH_4 adsorption isotherms were measured at temperatures ranging from 273 to 298 K, controlled by high precision custom-made cryostat (E-lab services, Czech Republic). Prior to adsorption measurements, the samples were degassed for 2 h under a vacuum generated by the turbomolecular pump at room temperature (samples denoted –RT) or at 70°C (samples denoted –70C). Adsorption data was evaluated by MicroActive data reduction software (v4.2, Micromeritics, USA). The specific surface area was evaluated from nitrogen adsorption isotherms by B.E.T. theory applied to the data in an interval of relative pressures satisfying the condition of the positive derivation of the data in the so-called Rouquerol plot ($n_{ads}(1\text{-}P/P_0)$ vs P/P_0 dependence). CO_2 adsorption isotherms measured at 273 K were subjected to pore size distribution by applying the NL DFT approach using the relevant kernel (CO2@273-Carbon Slit Pores 10 atm).

Thermogravimetry

TG experiment was measured on Netzsch STA 449F5 Jupiter TG-DSC instrument. The mass of **5h** sample freshly taken from the hexane mother liquor was 3.65 mg.

Supplementary Text

Synthesis

Compounds **2**–**5** were prepared by modified Negishi coupling of iodinated thiaborane 12-I-*closo*-1-$SB_{11}H_{10}$ with the corresponding organozinc reagent (4-X-C_6H_4-ZnBr, where X = OMe, SMe, Ph or NMe_2) catalyzed by $PdCl_2(PPh_3)_2$ (Fig. S1). Though harsh reaction conditions (high loading of catalyst, 4 equivalents of the organozinc reagent, and heating to reflux) were needed in order to prepare desired adducts, all compounds were isolated in moderate (40 % for **5**) to high yields (80–85 % for **2**–**4**). All compounds were characterized in the solution by multinuclear NMR spectroscopy. ^{1}H and ^{13}C NMR spectra revealed one set of expected signals for the organic moieties. ^{11}B NMR spectra exhibited an identical pattern of 1:5:5 with the signal of the B12 boron atom being shifted upfield compared to the parent thiaborane ($\Delta\delta(^{11}B) \sim 27.5$ ppm).

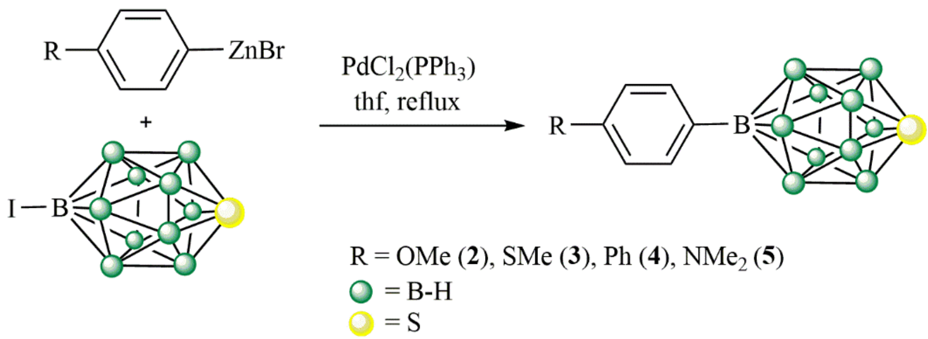

Figure S1: General scheme of the coupling reactions of 12-I-*closo*-1-$SB_{11}H_{10}$ with organozinc reagents.

Synthesis of 12-(4-OMe-C_6H_4)-1-$SB_{11}H_{10}$ (**2**)

A solution of 4-bromoanisole (0.17 mL, 1.32 mmol) in thf (5 mL) was added to a stirred suspension of magnesium turnings (96 mg, 3.96 mmol) in thf (5 mL). The reaction mixture was heated to reflux for one hour and then cooled down to room temperature. The suspension was filtered off, and the colourless filtrate was added dropwise to a solution of zinc chloride (180 mg, 1.32 mmol) at 0 °C. The reaction mixture was heated to reflux for 30 minutes and again cooled to room temperature. The solution of the organozinc reagent was added dropwise to a solution of 12-I-1-$SB_{11}H_{10}$ (95 mg, 0.33 mmol) and $(Ph_3P)_2PdCl_2$ (12 mg, 0,016 mmol) in thf (10 mL). The orange solution was heated to reflux for two hours, forming a dark brown suspension. After cooling to room temperature, the reaction mixture was added to a stirred mixture of concentrated hydrochloric acid/water/hexane (50 mL, 1:3:1). The organic layer was separated, washed once with brine (10 mL), and dried with magnesium sulfate. Evaporation of the slightly orange solution and recrystallization of the solid from hexane yielded **2** in the form of colourless crystals. Yield 72 mg (85%). Mp 90 °C. Anal. Calc. for $C_7H_{17}B_{10}OS$ (257.38): C 32.7, H 6.7; found C 32.8, H 6.8. ^{1}H NMR (25 °C, C_6D_6, 500 MHz): δ = 1.48–3.78 (m broad, 10H, B*H*), 3.30 (s, 3H, OC*H*$_3$), 6.81 (d, $^{3}J(^{1}H\text{-}^{1}H)$ = 7.9 Hz, 2H, *m*-Ar*H*), 7.51 (d, $^{3}J(^{1}H\text{-}^{1}H)$ = 7.9 Hz, 2H, *o*-Ar*H*) ppm. ^{11}B NMR (25 °C, C_6D_6, 160.42 MHz): δ = –6.7 (d, $^{1}J(^{1}H\text{-}^{11}B)$ = 182 Hz, 5B, B2,3,4,5,6), –3.9 (d, $^{1}J(^{1}H\text{-}^{11}B)$ = 152 Hz, 5B, B7,8,9,10,11), 27.5 (s, 1B, B12) ppm. $^{13}C\{^{1}H\}$ NMR (25 °C, C_6D_6, 125.76 Hz): δ = 54.9 (s, OC*H*$_3$), 113.9 (s, *m*-Ar*C*), 133.2 (s, *o*-Ar), 136.0 (s broad, *ipso*-Ar*C*), 160.7 (s, *p*-Ar*C*) ppm.

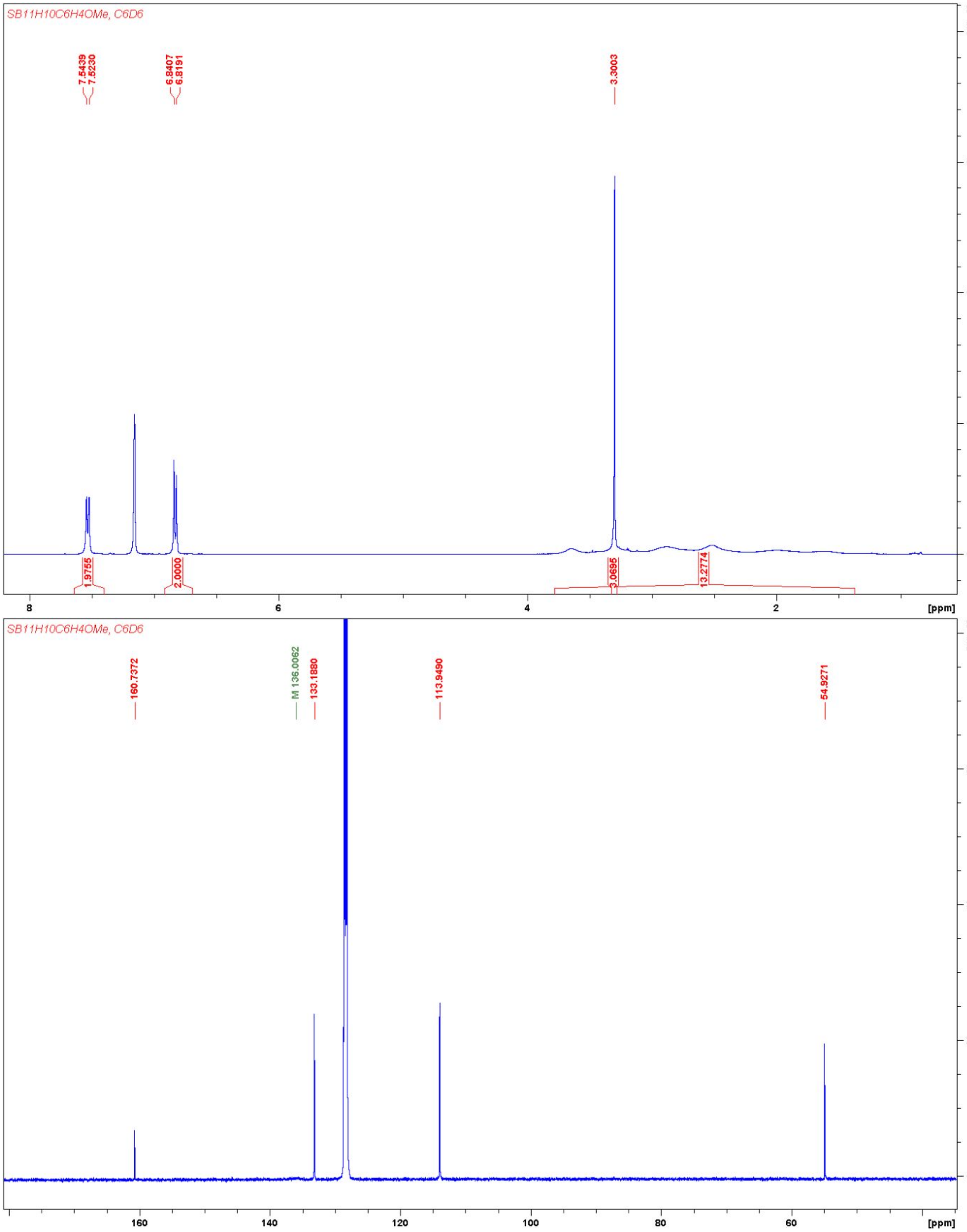

Figure S2: ${}^{1}H$ (top) and ${}^{13}C$ (bottom) NMR spectrum of 12-(4-OMe-C_6H_4)-1-$SB_{11}H_{10}$ (**2**).

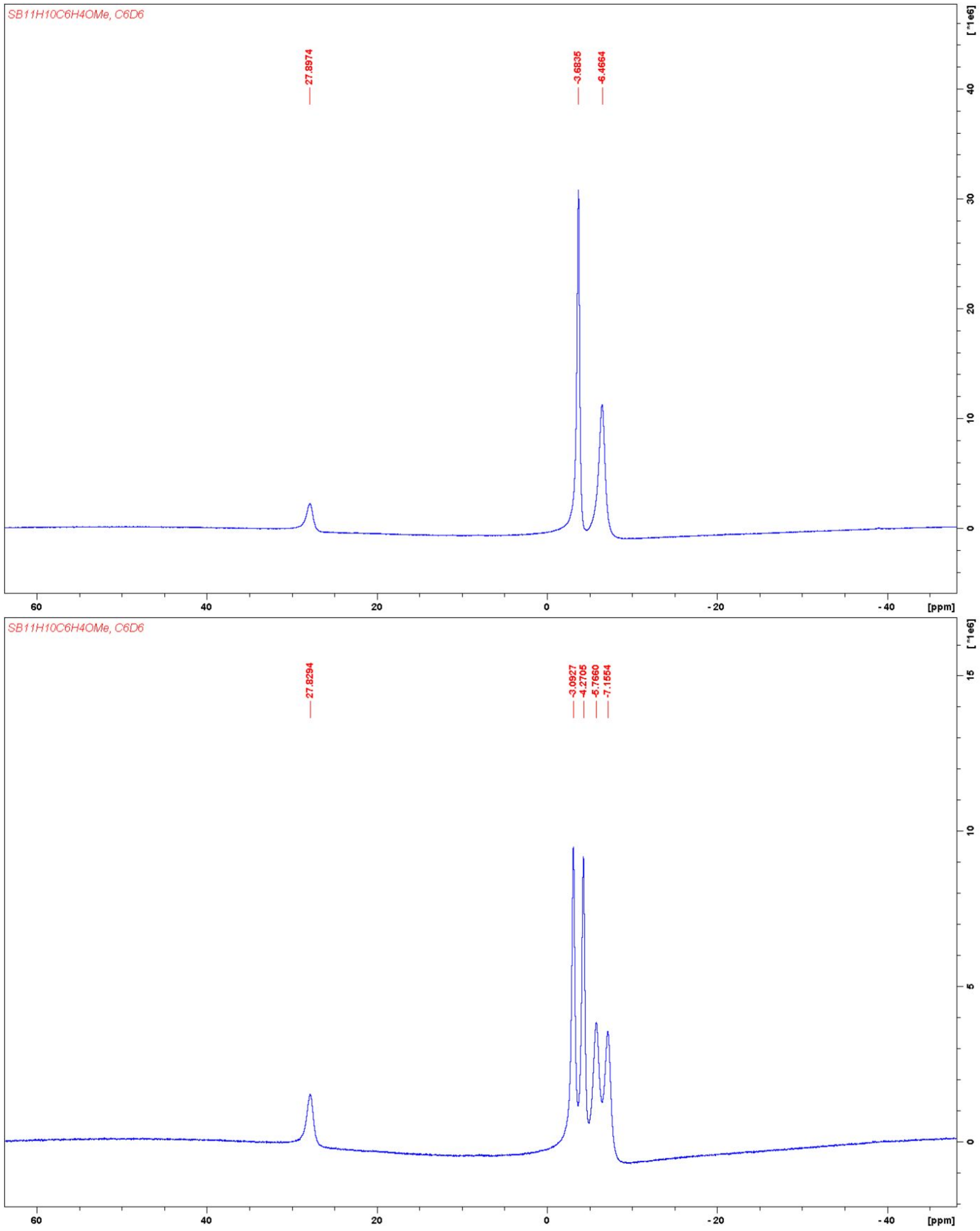

Figure S3: $^{11}B\{^1H\}$ (top) and ^{11}B (bottom) NMR spectrum of 12-(4-OMe-C_6H_4)-1-$SB_{11}H_{10}$ (**2**).

Synthesis of 12-(4-SMe-C_6H_4)-1-$SB_{11}H_{10}$ (**3**)

A solution of 4-bromothioanisole (282 mg, 1.39 mmol) in thf (5 mL) was added to a stirred suspension of magnesium turnings (101 mg, 4.17 mmol) in thf (5 mL). The reaction mixture was heated to reflux for one hour and then cooled down to room temperature. The suspension was filtered off, and the colourless filtrate was added dropwise to a solution of zinc chloride (189 mg, 1.39 mmol) at 0 °C. The reaction mixture was heated to reflux for 30 minutes and again cooled to room temperature. The solution of the organozinc reagent was added dropwise to a solution of 12-I-1-$SB_{11}H_{10}$ (100 mg, 0.35 mmol) and $(Ph_3P)_2PdCl_2$ (12 mg, 0,017 mmol) in thf (10 mL). The orange solution was heated to reflux for two hours, forming a dark brown suspension. After cooling to room temperature, the reaction mixture was added to a stirred mixture of concentrated hydrochloric acid/water/hexane (50 mL, 1:3:1). The organic layer was separated, washed once with brine (10 mL), and dried with magnesium sulfate. Evaporation of the slightly orange solution and recrystallization of the solid from hexane yielded **3** in the form of colourless crystals. Yield 78 mg (82%). Mp 133 °C. Anal. Calc. for $C_7H_{17}B_{10}S_2$ (273.45): C 30.8, H 6.3; found C 30.7, H 6.4. ^{1}H NMR (25 °C, C_6D_6, 500 MHz): δ = 1.99 (s, 3H, SCH_3), 7.14 (d, $^{3}J(^{1}H-^{1}H)$ = 8.1 Hz, 2H, Ar*H*), 7.46 (d, $^{3}J(^{1}H-^{1}H)$ = 8.1 Hz, 2H, Ar*H*) ppm. ^{11}B NMR (25 °C, C_6D_6, 160.42 MHz): δ = –6.5 (d, $^{1}J(^{1}H-^{11}B)$ = 177 Hz, 5B, B2,3,4,5,6), –3.8 (d, $^{1}J(^{1}H-^{11}B)$ = 151 Hz, 5B, B7,8,9,10,11), 27.1 (s, 1B, B12) ppm. $^{13}C\{^{1}H\}$ NMR (25 °C, C_6D_6, 125.76 Hz): δ = 15.3 (s, S*C*H_3), 126.1 (s, *m*-Ar*C*), 132.1 (s, *o*-Ar*C*), 138.9 (s, *p*-Ar*C*) ppm.

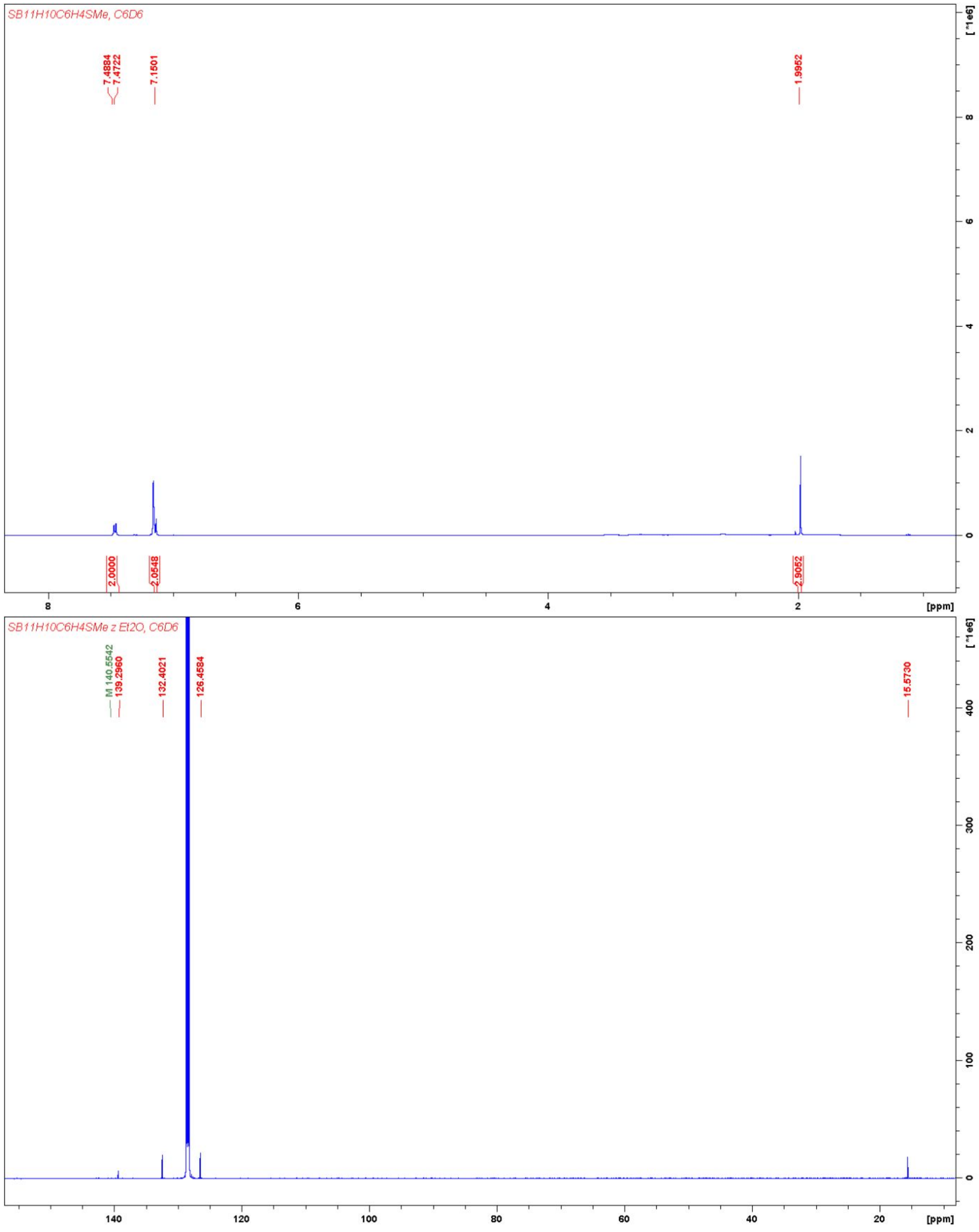

Figure S4: ^{1}H (top) and ^{13}C (bottom) NMR spectrum of 12-(4-SMe-C_6H_4)-1-$SB_{11}H_{10}$ (**3**).

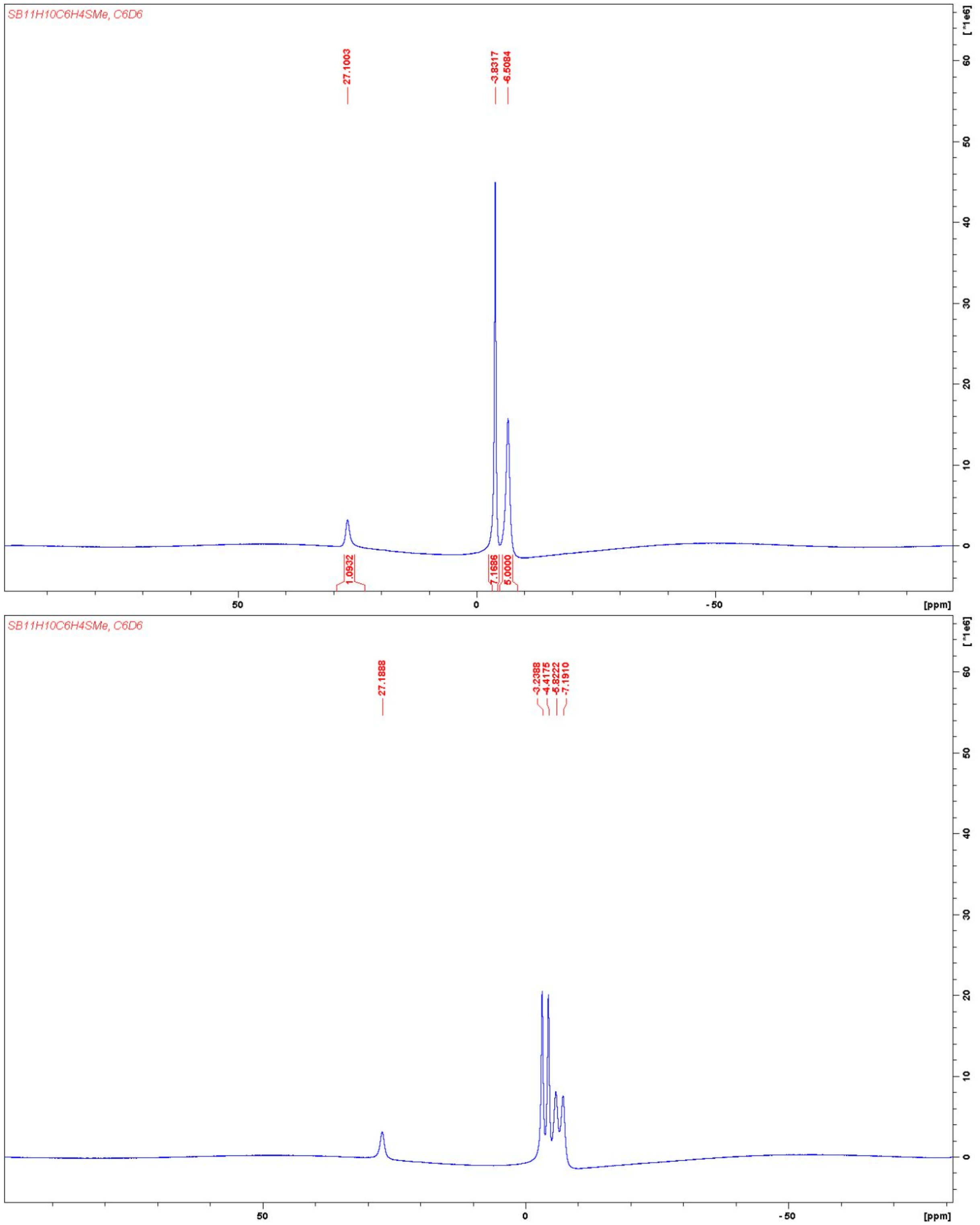

Figure S5: $^{11}B\{^{1}H\}$ (top) and ^{11}B (bottom) NMR spectrum of 12-(4-SMe-C_6H_4)-1-$SB_{11}H_{10}$ (**3**).

Synthesis of 12-(4-C_6H_5-C_6H_4)-1-$SB_{11}H_{10}$ (**4**)

A solution of 4-bromobiphenyl (314 mg, 1.35 mmol) in thf (5 mL) was added to a stirred suspension of magnesium turnings (98 mg, 4.04 mmol) in thf (5 mL). The reaction mixture was heated to reflux for one hour and then cooled down to room temperature. The suspension was filtered off, and the colourless filtrate was added dropwise to a solution of zinc chloride (184 mg, 1.35 mmol) at 0 °C. The reaction mixture was heated to reflux for 30 minutes and again cooled to room temperature. The solution of the organozinc reagent was added dropwise to a solution of 12-I-1-$SB_{11}H_{10}$ (97 mg, 0.34 mmol) and $(Ph_3P)_2PdCl_2$ (12 mg, 0,017 mmol) in thf (10 mL). The orange solution was heated to reflux for two hours, forming a dark brown suspension. After cooling to room temperature, the reaction mixture was added to a stirred mixture of concentrated hydrochloric acid/water/hexane (50 mL, 1:3:1). The organic layer was separated, washed once with brine (10 mL), dried with magnesium sulfate and evaporated. The solid residue was extracted with hexane to remove the traces of the catalyst and evaporated in vacuo. The residual biphenyl was removed by sublimation at 70 °C in *vacuo* giving **4** in the form of colourless crystals. Yield 108 mg (80%). Mp. 167 °C. Anal. Calc. for $C_{12}H_{19}B_{10}S$ (303.45): C 47.5, H 6.3; found C 47.3, H 6.2. ^{1}H NMR (25 °C, C_6D_6, 500 MHz): δ = 7.12 (t, $^3J(^1H-^1H)$ = 7.3 Hz, 1H, *p*-C_6H_5), 7.21 (t, $^3J(^1H-^1H)$ = 7.7 Hz, 2H, *m*-C_6H_5), 7.50 (d, $^3J(^1H-^1H)$ = 7.7 Hz, 4H, *m*-C_6H_4 + *o*-C_6H_5), 7.66 (d, $^3J(^1H-^1H)$ = 8.0 Hz, 2H, *o*-C_6H_4) ppm. ^{11}B NMR (25 °C, C_6D_6, 160.42 MHz): δ = –6.6 (d, $^1J(^1H-^{11}B)$ = 170 Hz, 5B, B2,3,4,5,6), –3.8 (d, $^1J(^1H-^{11}B)$ = 150 Hz, 5B, B7,8,9,10,11), 27.1 (s, 1B, B12) ppm. $^{13}C\{^1H\}$ NMR (25 °C, C_6D_6, 125.76 Hz): δ = 127.1 (s, *m*-C_6H_4), 127.7 (s, *p*-C_6H_5), 128.0 (s, *o*-C_6H_5), 129.4 (s, *m*-C_6H_5), 132.5 (s, *o*-C_6H_4), 141.5 (s, *p*-C_6H_4), 142.2 (s, *ipso*-C_6H_5), 142.8 (s, *ipso*-C_6H_4) ppm.

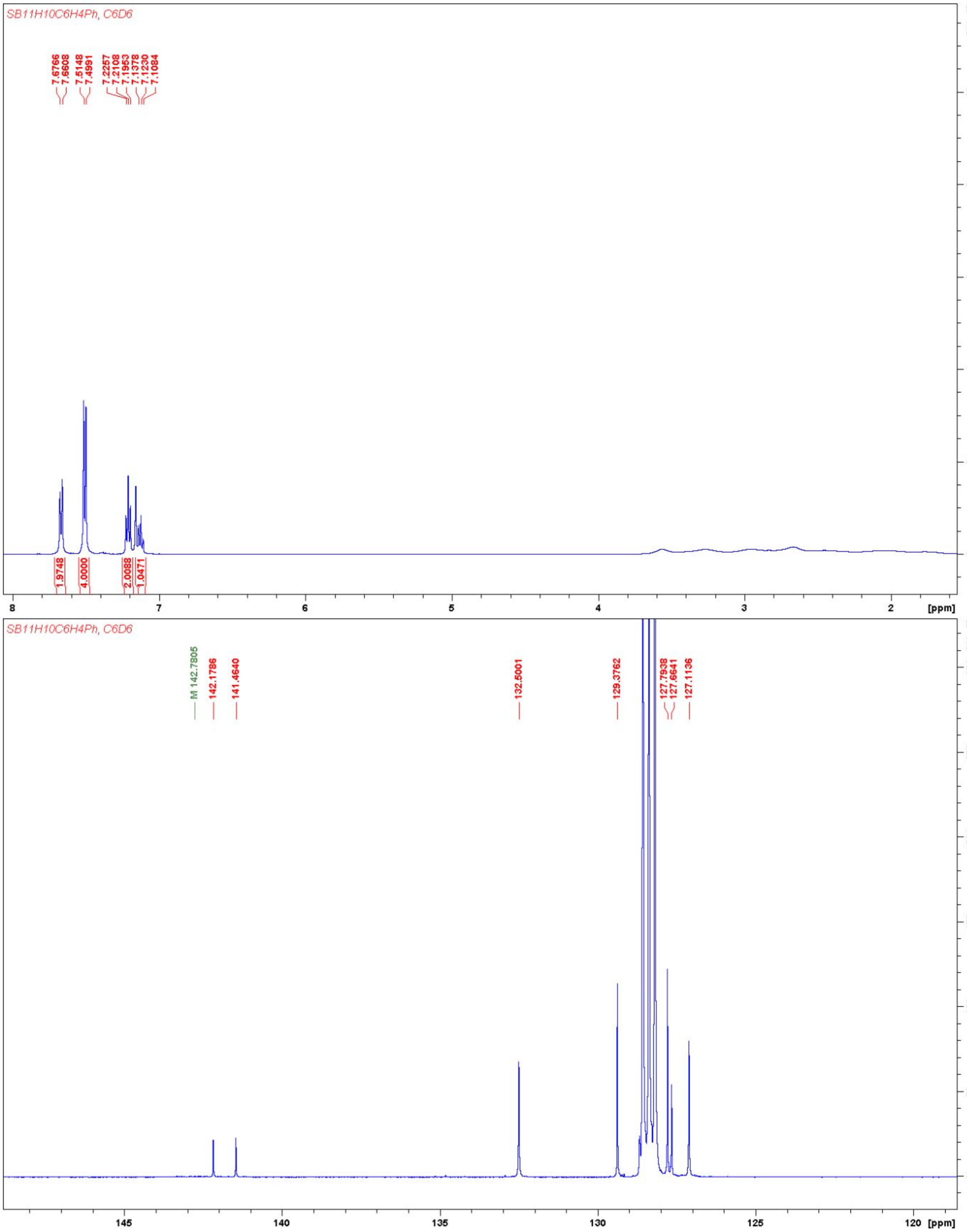

Figure S6: ^{1}H (top) and ^{13}C (bottom) NMR spectrum of 12-(4-C_6H_5-C_6H_4)-1-$SB_{11}H_{10}$ (**4**).

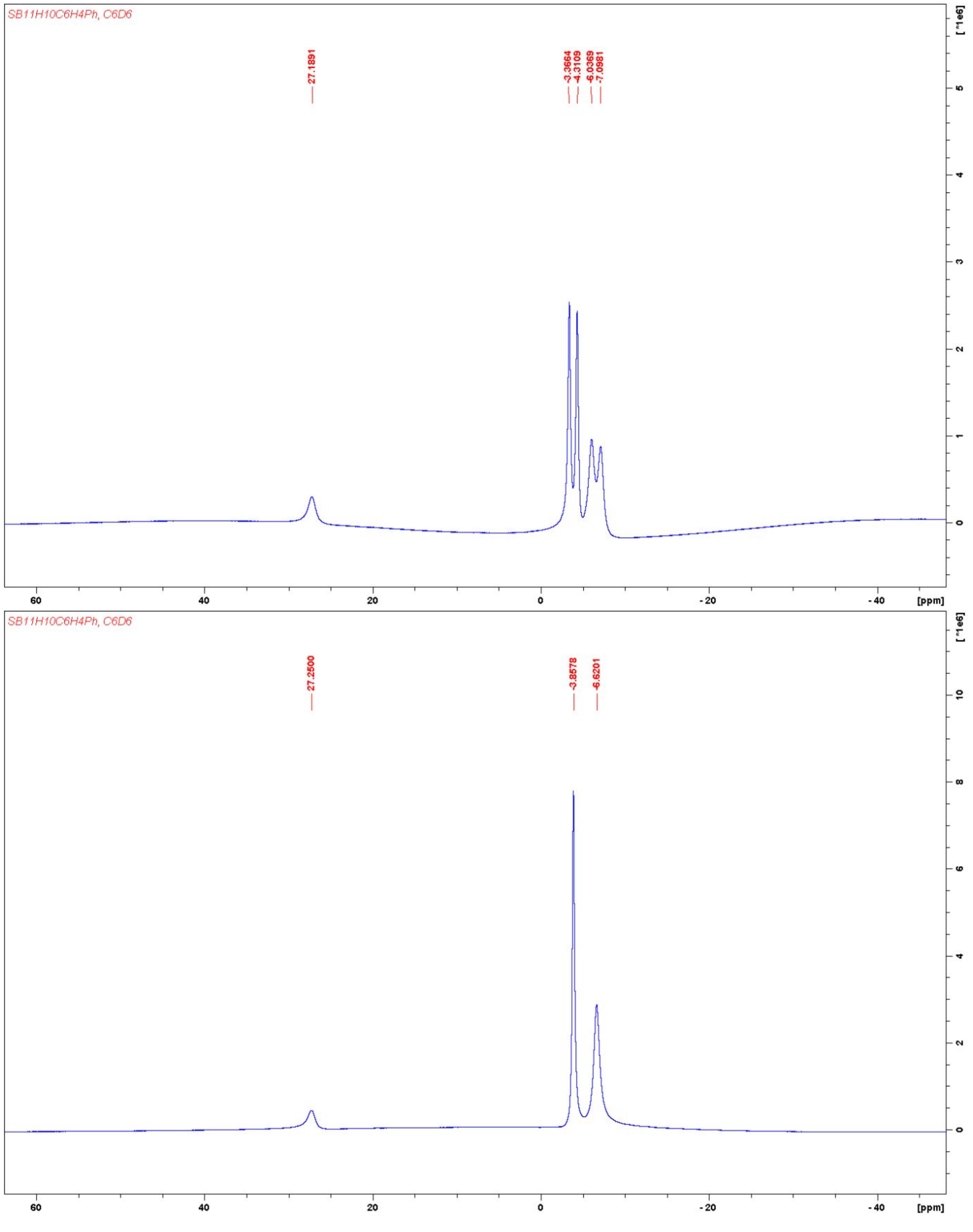

Figure S7: $^{11}B\{^{1}H\}$ (top) and ^{11}B (bottom) NMR spectrum of 12-(4-C_6H_5-C_6H_4)-1-$SB_{11}H_{10}$ (**4**).

Synthesis of 12-(4-NMe_2-C_6H_4)-1-$SB_{11}H_{10}$ (**5**)

A solution of 4-bromo-*N*,*N*-dimethylaniline (292 mg, 1.46 mmol) in thf (5 mL) was added to a stirred suspension of magnesium turnings (106 mg, 4.38 mmol) in thf (5 mL). The reaction mixture was heated to reflux for one hour and then cooled down to room temperature. The suspension was filtered off, and the colourless filtrate was added dropwise to a solution of zinc chloride (199 mg, 1.46 mmol) at 0 °C. The reaction mixture was heated to reflux for 30 minutes and again cooled to room temperature. The solution of the organozinc reagent was added dropwise to a solution of 12-I-1-$SB_{11}H_{10}$ (105 mg, 0.36 mmol) and $(Ph_3P)_2PdCl_2$ (12 mg, 0,017 mmol) in thf (10 mL). The orange solution was heated to reflux for two hours, forming a dark brown suspension. After cooling to room temperature, the reaction mixture was added to a stirred mixture of concentrated hydrochloric acid/water/hexane (50 mL, 1:3:1). The aqueous layer was separated and neutralized with a saturated solution of $NaHCO_3$. The suspension was extracted two times with benzene (2x10 mL). The organic layer was separated, washed once with brine (10 mL), and dried with magnesium sulfate. Evaporation of the slightly brown solution and recrystallization of the solid from hexane yielded **4** in the form of colourless crystals. Yield 35 mg (35%). Mp 144 °C. Anal. Calc. for $C_8H_{20}B_{11}NS$ (273.45): C 34.2, H 7.2; found C 34.3, H 7.4. ^{1}H NMR (25 °C, C_6D_6, 500 MHz): δ = 2.51 (s, 6H, NCH_3), 6.63 (d, $^{3}J(^{1}H-^{1}H)$ = 8.6 Hz, 2H, Ar*H*), 7.58 (d, $^{3}J(^{1}H-^{1}H)$ = 8.6 Hz, 2H, Ar*H*) ppm. ^{11}B NMR (25 °C, C_6D_6, 160.42 MHz): δ = –6.7 (d, $^{1}J(^{1}H-^{11}B)$ = 178 Hz, 5B, B2,3,4,5,6), –3.9 (d, $^{1}J(^{1}H-^{11}B)$ = 152 Hz, 5B, B7,8,9,10,11), 28.3 (s, 1B, B12) ppm. $^{13}C\{^{1}H\}$ NMR (25 °C, C_6D_6, 125.76 Hz): δ = 39.8 (s, NCH_3), 112.07, 132.1, 150.5 (s, Ar*C*) ppm.

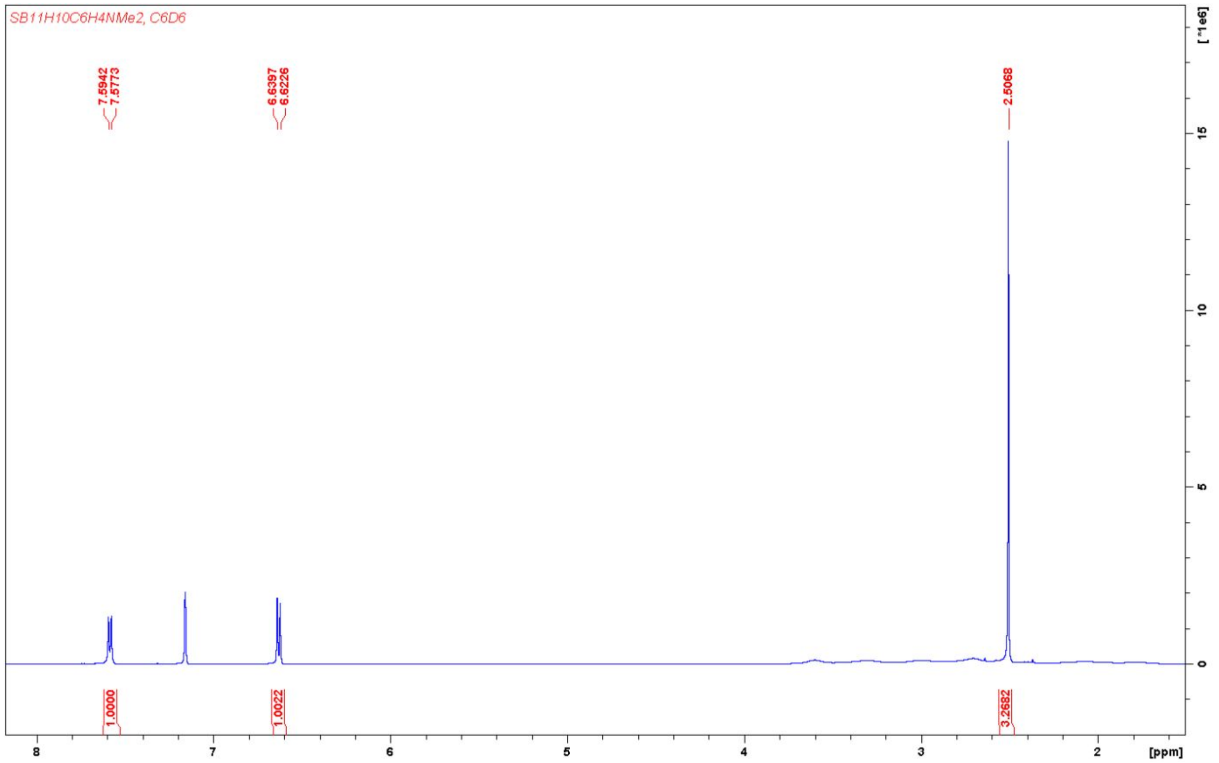

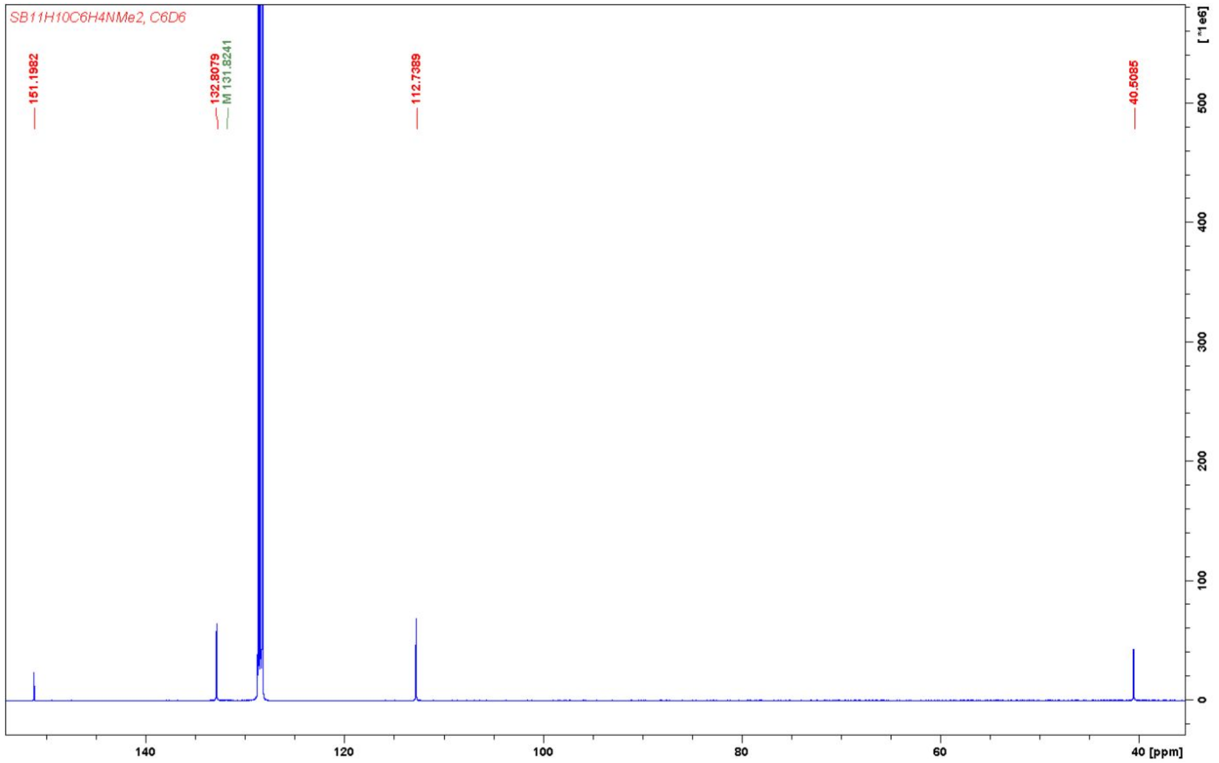

Figure S8: ^{1}H (top) and ^{13}C (bottom) NMR spectrum of 12-(4-NMe_2-C_6H_4)-1-$SB_{11}H_{10}$ (**5**).

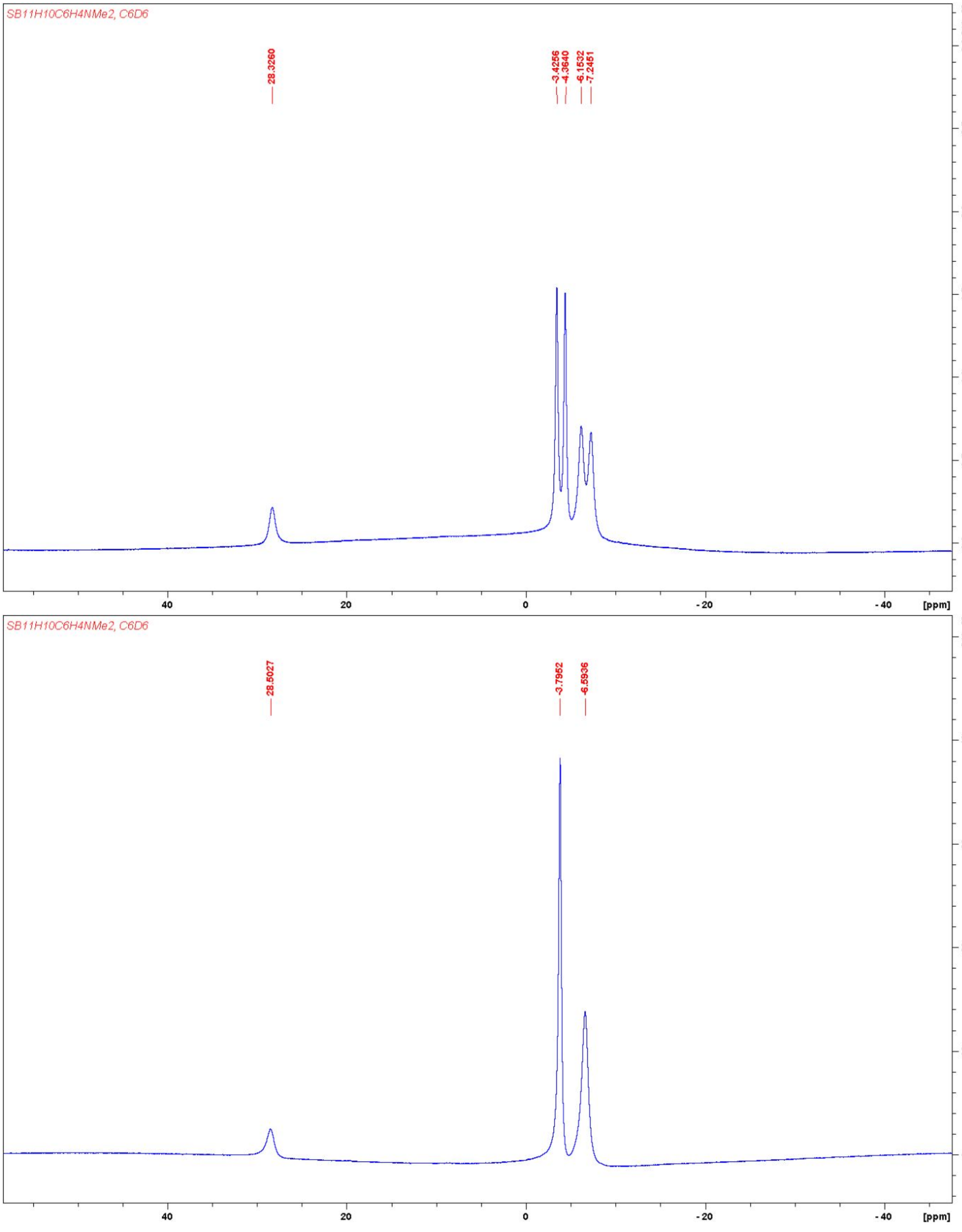

Figure S9: $^{11}B\{^{1}H\}$ (top) and ^{11}B (bottom) NMR spectrum of 12-(4-NMe_2-C_6H_4)-1-$SB_{11}H_{10}$ (**5**).

Crystallographic section

The crystal structures of 13 different compounds and solvatopolymorphs were determined by sc-XRD techniques. Out of these compounds, only **2** (S…Cg ~ 6Å) does not exhibit an interaction via a chalcogen bond of sulfur atom and an aromatic ring of substituted thiaborane. The separations of sulfur and the centroid of the appropriate ring and the angle of the B12 atom, sulfur, and the centroid of the interacting aromatic ring of the rest of the compounds is given in Table S1. In fact, the solvatopolymorphs and sublimed material of **5** generally exhibit shorter interactions by 0.1 Å than the other compounds. The value of the B12-S-Cg angle, which could be taken as an evaluation criterion for the existence of the S…Cg interaction (see the computational part) as well, is found in the range ~ 160–180°. Lower values sign improper orientation of the molecules. In the case of **5h**, the value is very close to the straight angle, expressing the fact this solvatopolymorph is the only compound where the interactions of four molecules of **5** into the closed square-shaped cycle.

The molecular structures of **2**–**5** (and all solvatopolymorphs) are shown in Figs. S10–S22, along with the orientation of solvates, crystal packing details, and cavities/tunnels formed by the removal of solvents. The removal of the small molecules is carried out experimentally for **5h** (decrease of **5**/*n*-hexane molar ratio from 1/4 to 1/16 when dry crystals are exposed to air, a complete removal is accomplished by dynamic vacuo of 1 mbar at 70°C for one hour), **5e** (diethylether is removed spontaneously on the air) or by the sublimation of **5** at 130 °C. Other low boiling point solvates can be removed either partially or upon a destruction of the crystal lattice, as in the cases of benzene, hexafluorobenzene, carbon tetrachloride, and carbon disulfide. Removal of higher boiling point solvents such as mesitylene or decalin is not possible at temperature < 80°C even when high dynamic vacuo is applied. In the later two groups, the solvates were removed artificially by PLATON/SQUEEZE[31] program, masking the potential solvent accessible volume, contact volume with the probe radius of 1.2 Å is shown using Mercury 4.3.0 program.[32] The solvent removal or masking resulted in cavities or tunnels of 11.5 (for **5h**) up to 23.4% (for **5cs**) of the unit cell volume. Hypothetical cavities after virtual removal of high boiling solvents by PLATON/SQUEEZE[31] program, such as benzene, hexafluorobenzene, carbon tetrachloride, carbon disulfide, mesitylene or decalin are shown just for comparison.

Table S1: Selected parameters of intermolecular S•••π contacts in compounds studied.

Compound	Distance Cg…S (Å)	Angle B12-S-Cg(°)
**3**	3.141<br>3.143	160.36<br>162.48
**4b**	3.609	132.68
**4h**	3.119	165.79
**5sub**	2.965	164.19
**5b**	3.007<br>3.025	159.39<br>161.07
**5cl**	3.037<br>2.993<br>2.985<br>2.934	159.21<br>167.16<br>158.95<br>166.39
**5cs**	3.077	158.72
**5d**	3.028	161.11
**5e**	2.973	163.82
**5f**	3.035<br>3.188	163.42<br>163.87
**5h**	3.068	179.48
**5m**	3.080<br>3.104	157.86<br>155.46

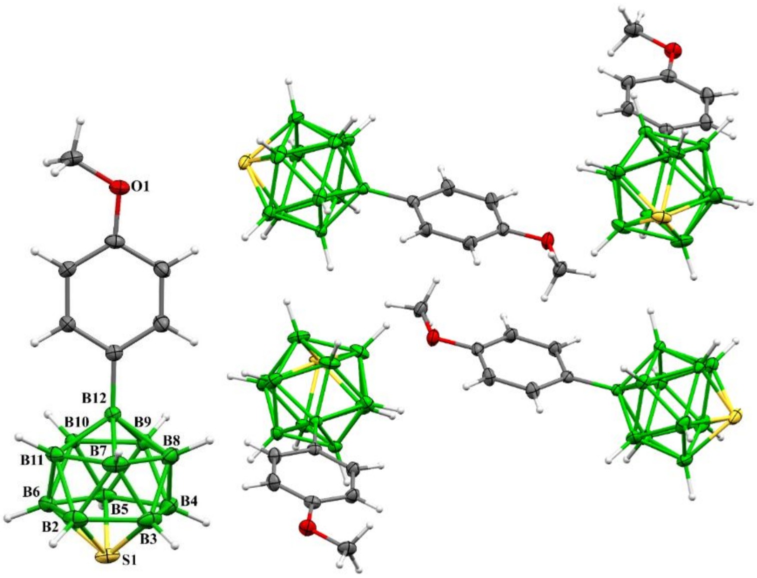

Figure S10: The molecular structure and a fragment of the crystal packing of **2**. Thermal ellipsoids are drawn with the 40% probability level.

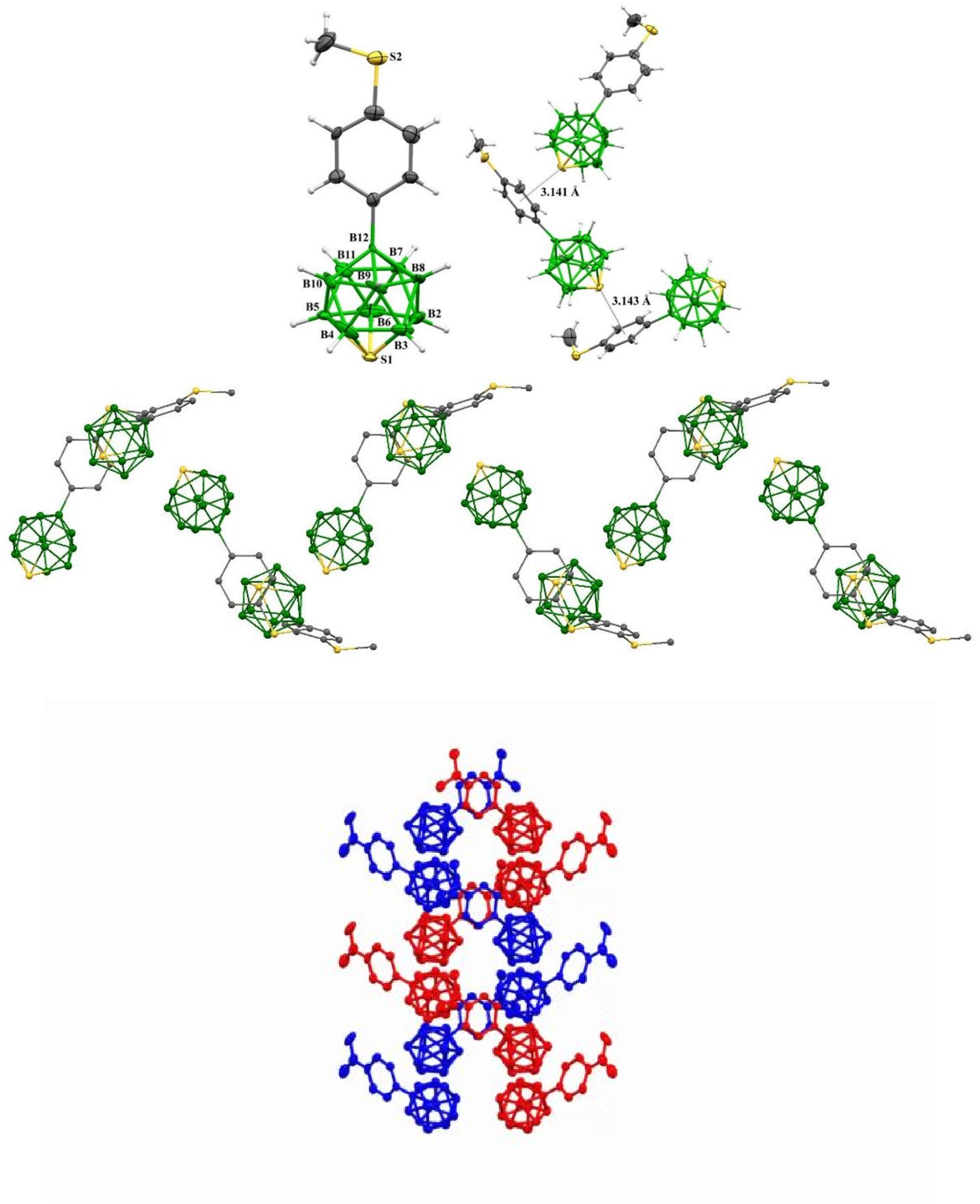

Figure S11: The molecular structure, a fragment of the crystal packing, a part of helical structure and schematic view of the double-helix structure of **3**. Thermal ellipsoids are drawn with the 40% probability level.

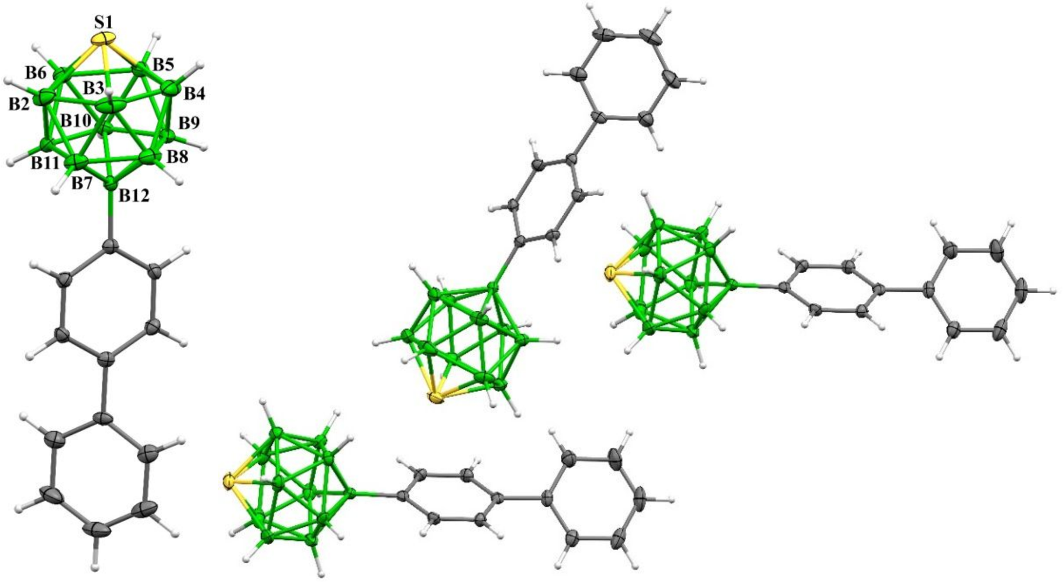

Figure S12: The molecular structure and a fragment of the crystal packing of **4b**. Thermal ellipsoids are drawn with the 40% probability level.

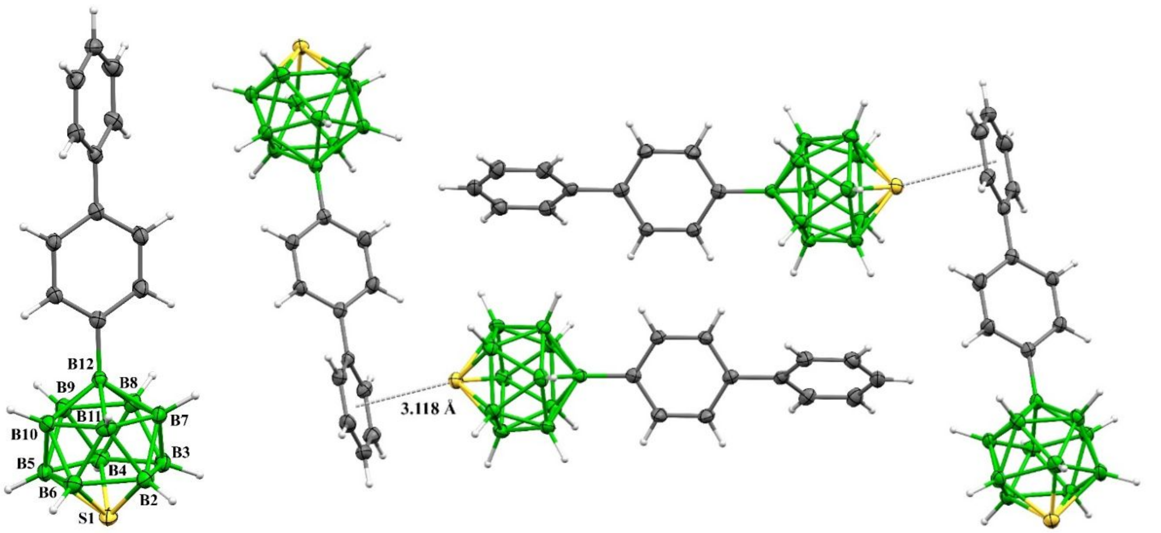

Figure S13: The molecular structure and a fragment of the crystal packing of **4h**. Thermal ellipsoids are drawn with the 40% probability level.

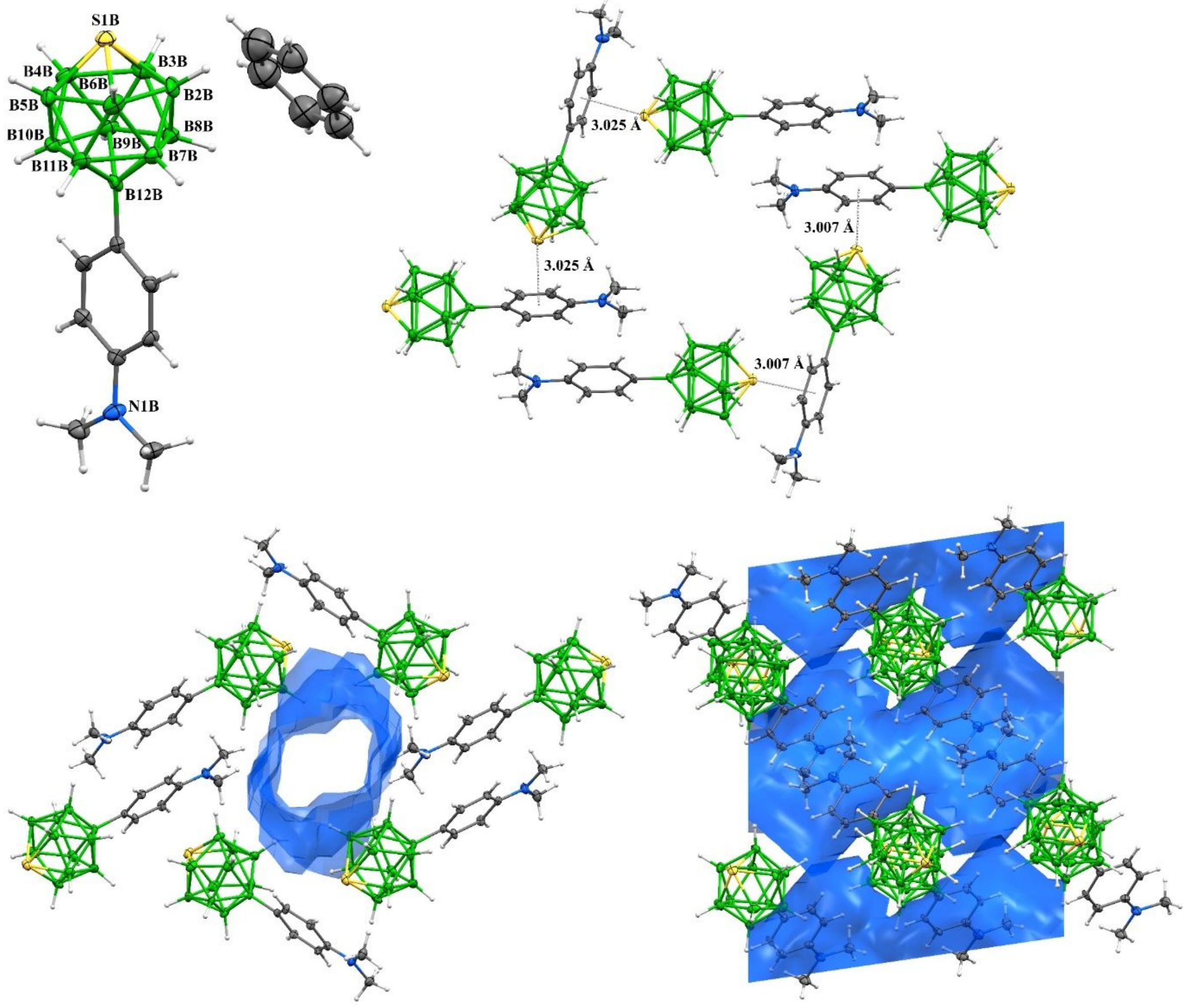

Figure S14: The molecular structure and a fragment of the crystal packing of **5b** (top). Voids formed when solvent molecules are removed by SQUEZZE procedure[31] (769.69 Å^3 or 20.4 % of the unit cell volume) (bottom). Thermal ellipsoids are drawn with the 40% probability level.

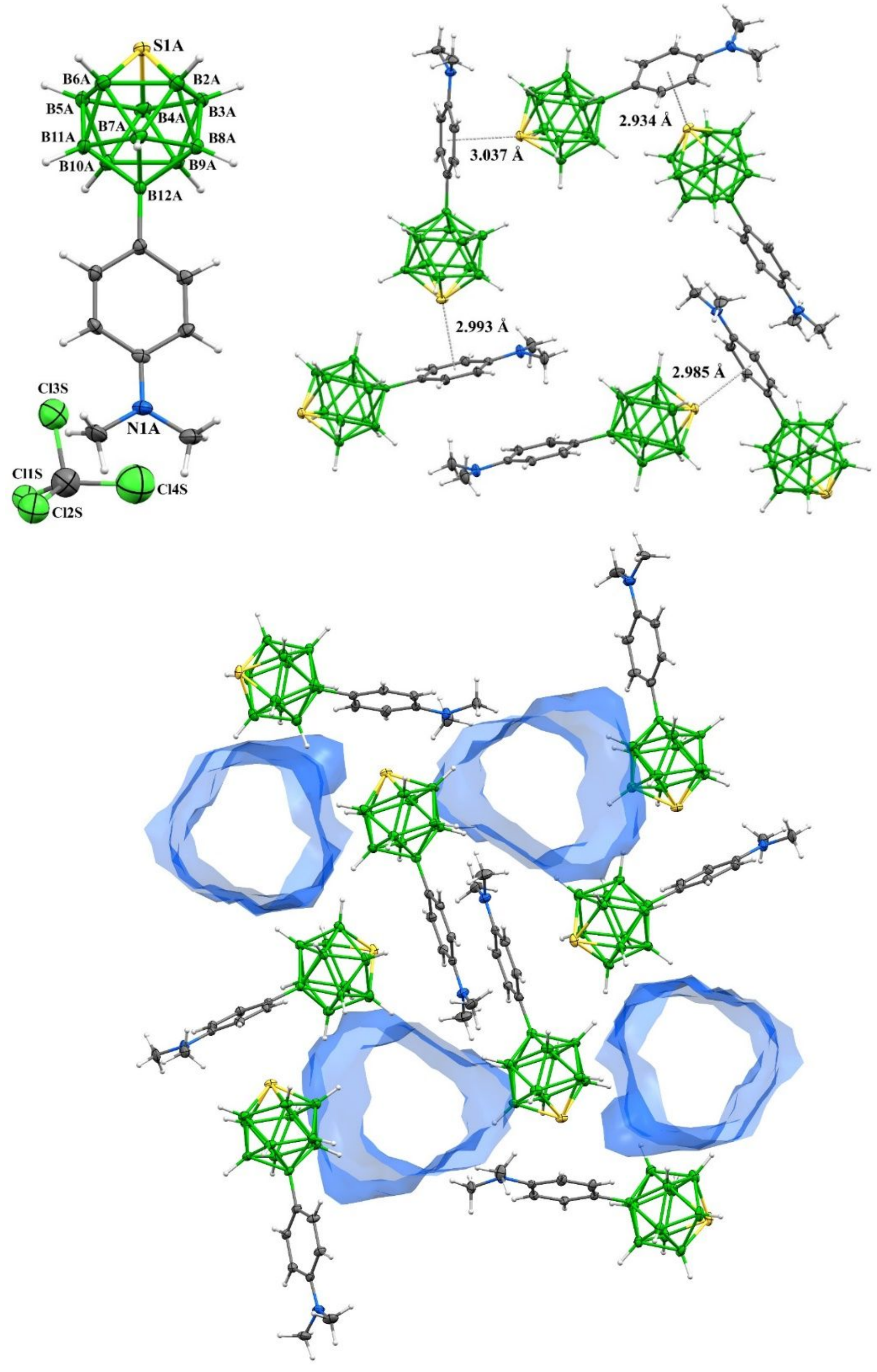

Figure S15: The molecular structure and fragment of crystal package of **5cl** (top). Voids formed when solvent molecules are removed by SQUEZZE procedure[31] (756.58 Å^3 or 19.5% of unit cell volume) (bottom). Thermal ellipsoids are drawn with the 40% probability level.

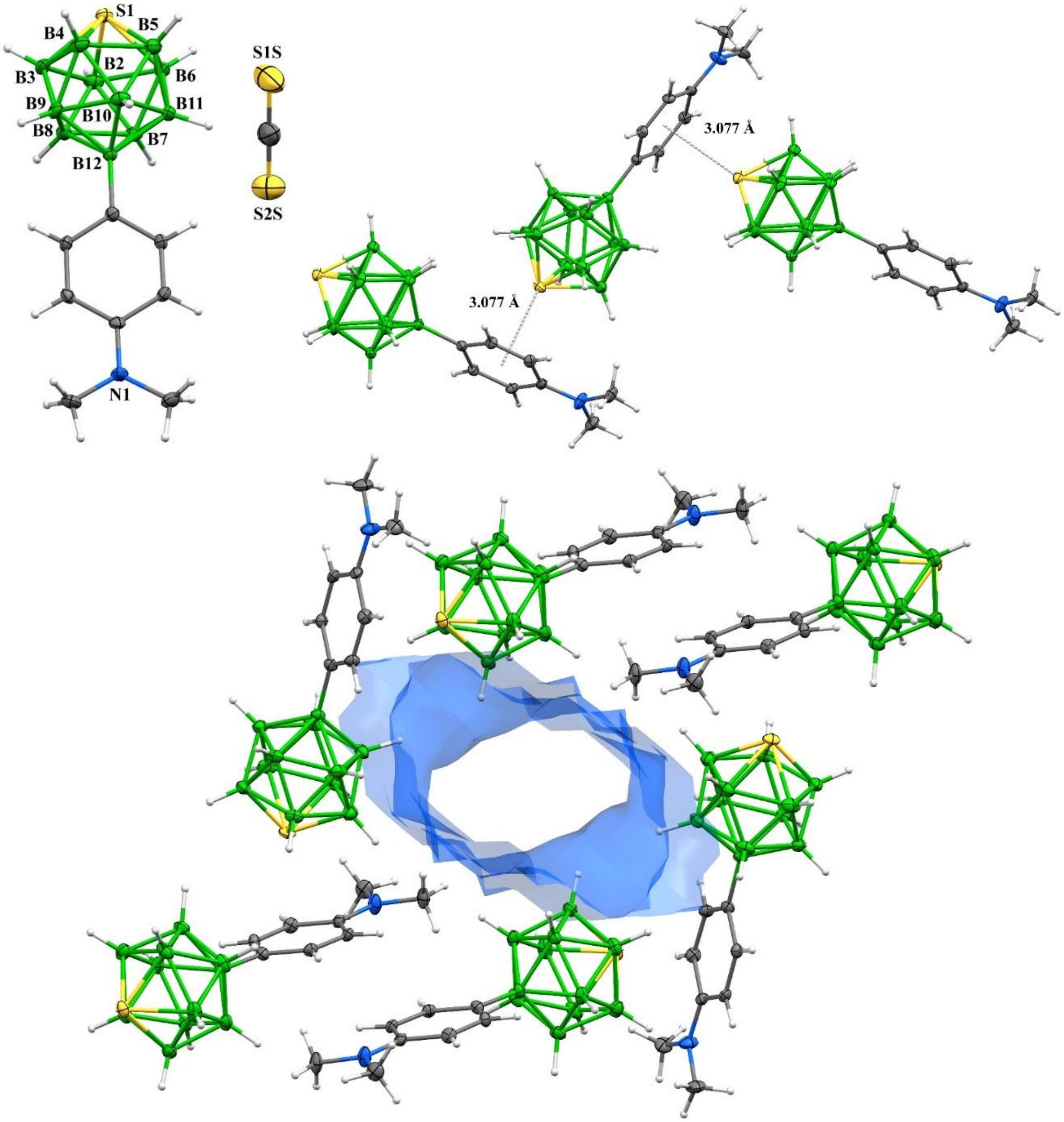

Figure S16: The molecular structure and fragment of crystal package of **5cs** (top). Voids formed when solvent molecules are removed by SQUEZZE procedure[31] (447.19 Å^3 or 23.4% of unit cell volume) (bottom). Thermal ellipsoids are drawn with the 40% probability level.

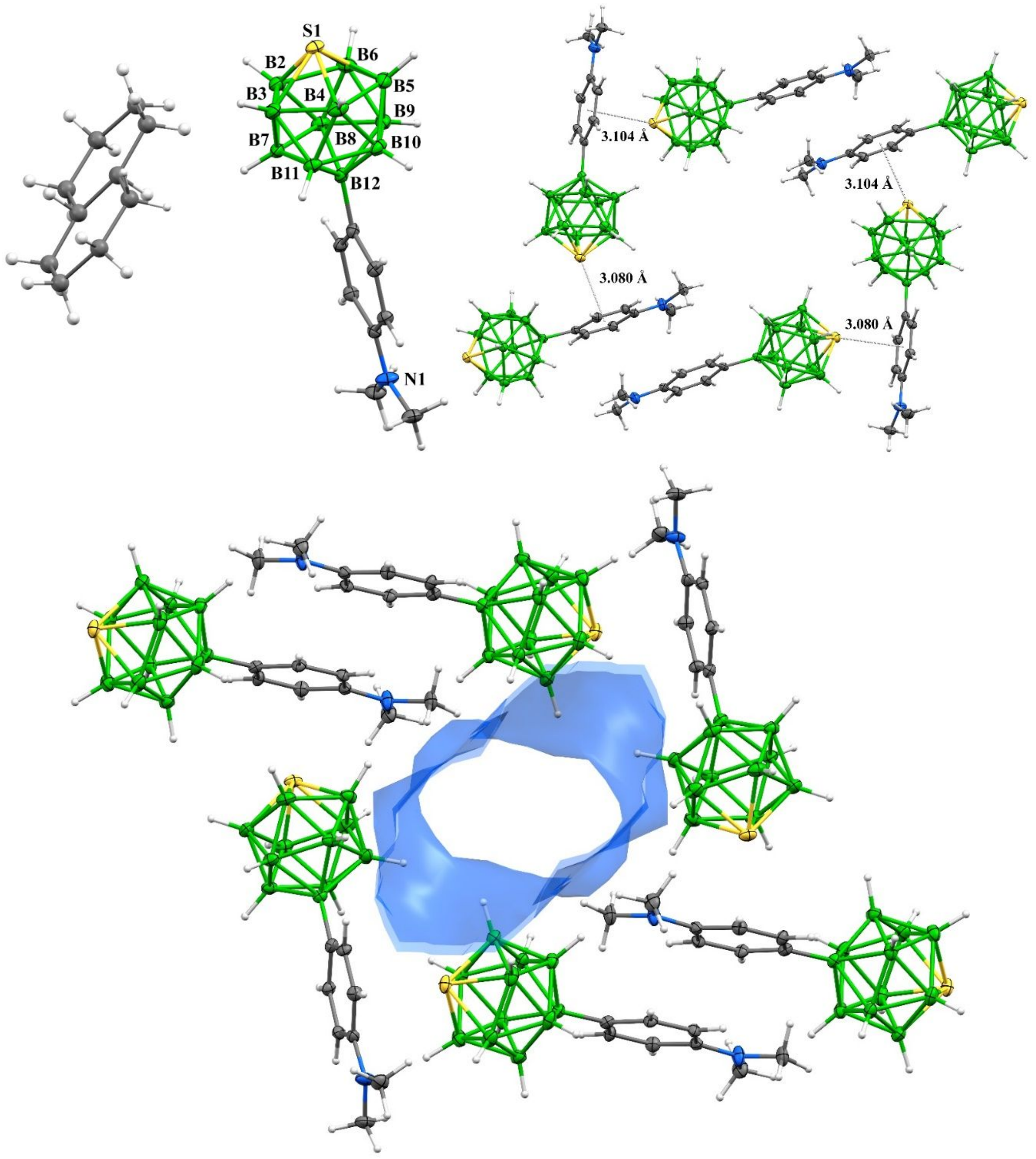

Figure S17: The molecular structure and fragment of crystal package of **5d** (top). Voids formed when solvent molecules are removed by SQUEZZE procedure[31] (443.68 Å^3 or 23.3 % of unit cell volume) (bottom). Thermal ellipsoids are drawn with the 40% probability level.

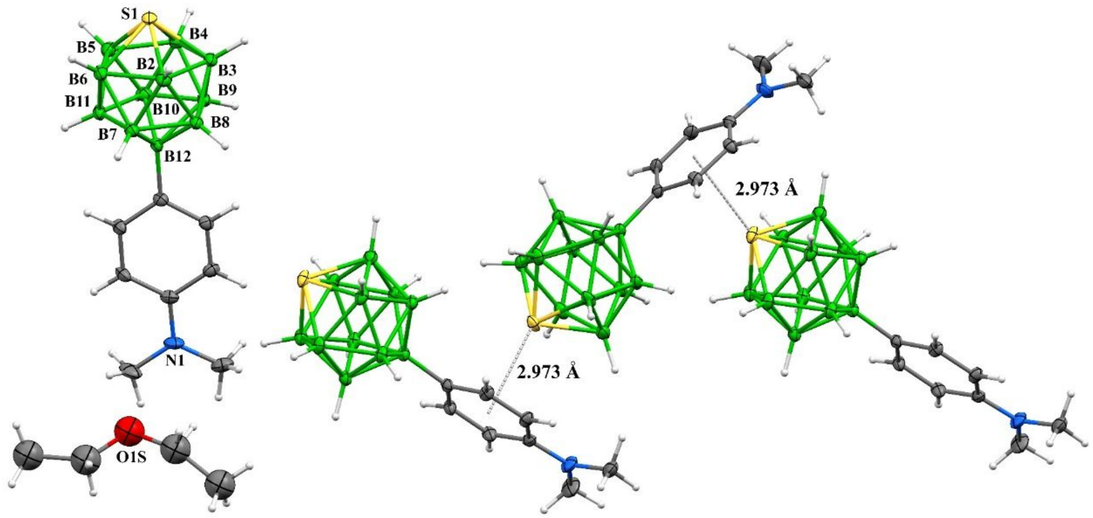

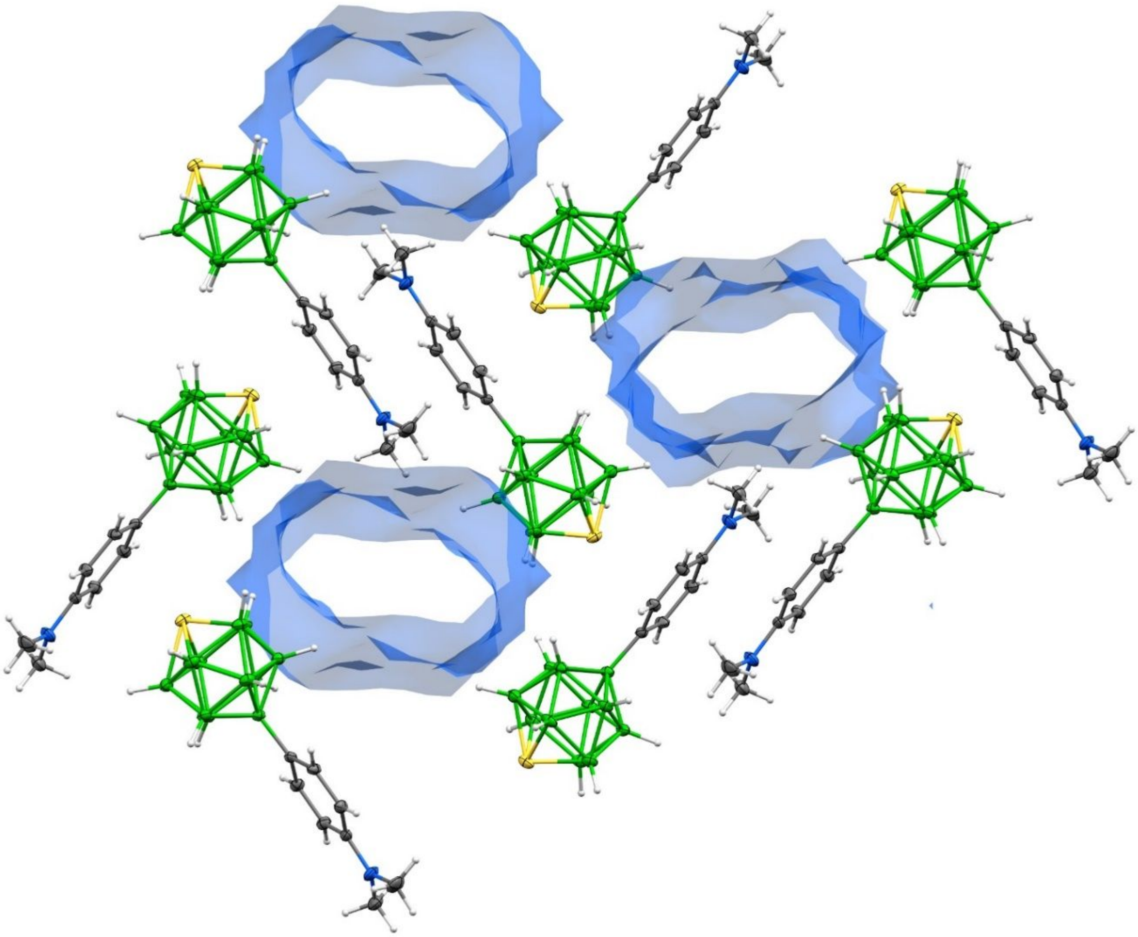

Figure S18: The molecular structure and fragment of crystal package of **5e** (top). Voids formed when solvent molecules are removed by SQUEZZE procedure[31] or crystals were exposed to air (390.88 Å^3 or 20.5 % of the unit cell volume) (bottom). Thermal ellipsoids are drawn with the 40% probability level.

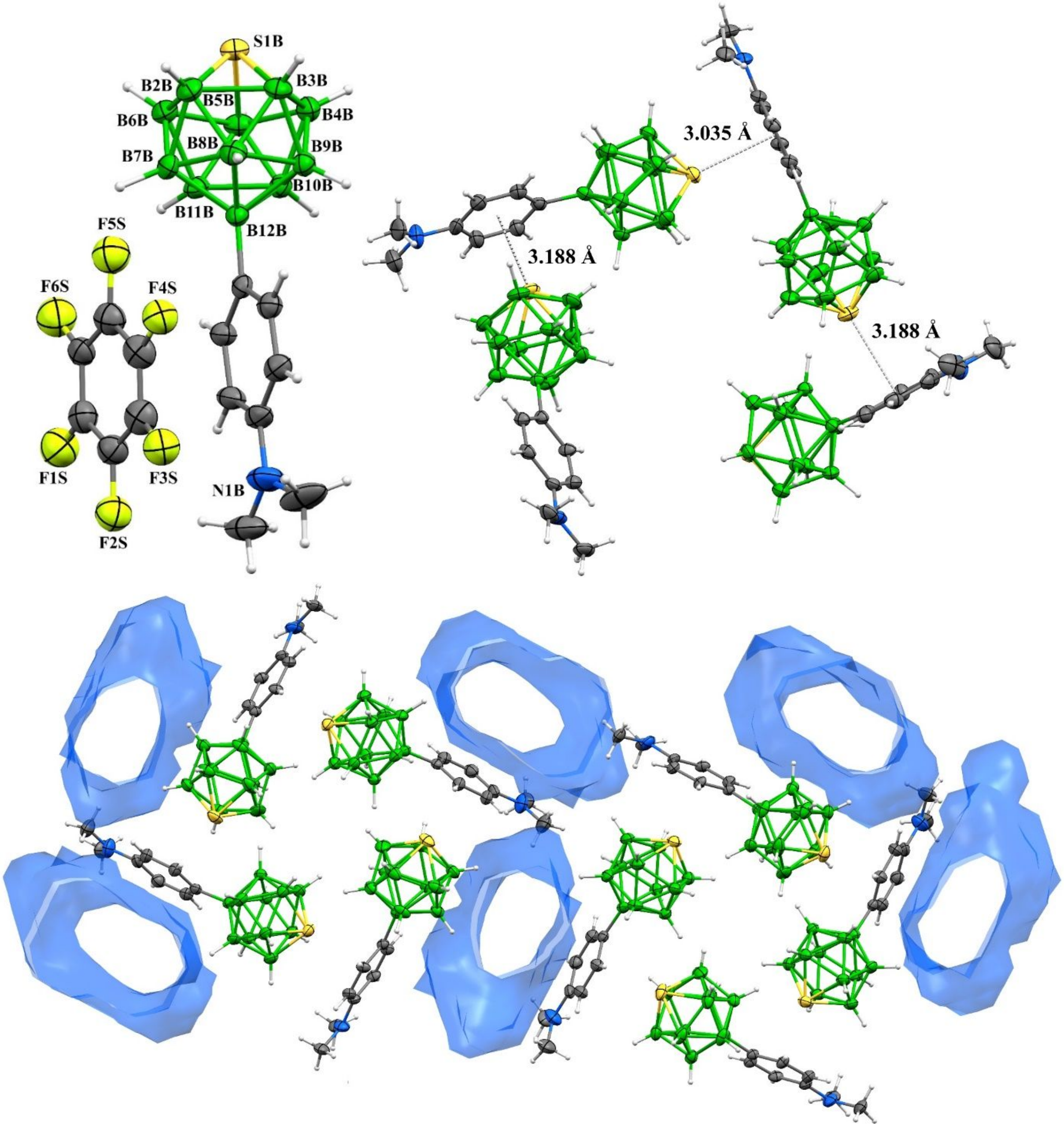

Figure S19: The molecular structure and fragment of crystal package of **5f** (top). Voids formed when solvent molecules are removed by SQUEZZE procedure[31] (2702.98 $Å^3$ or 17.3 % of the unit cell volume) (bottom). Thermal ellipsoids are drawn with the 40% probability level.

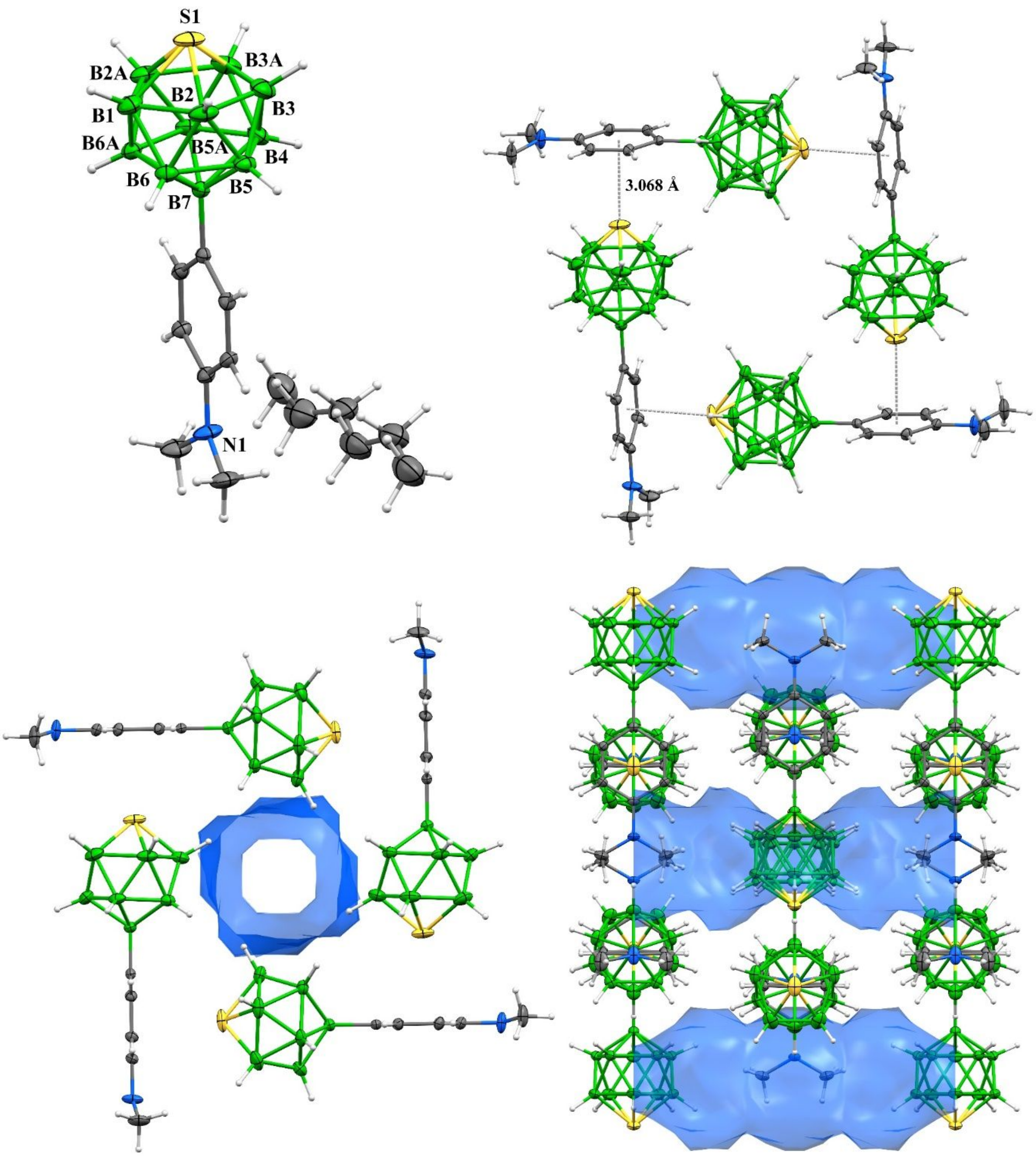

Figure S20: The molecular structure and fragment of crystal package of **5h** (top). Voids formed when solvent molecules are removed by vacuo (408.89 Å^3 or 11.5 % of the unit cell volume) (bottom). Thermal ellipsoids are drawn with the 40% probability level.

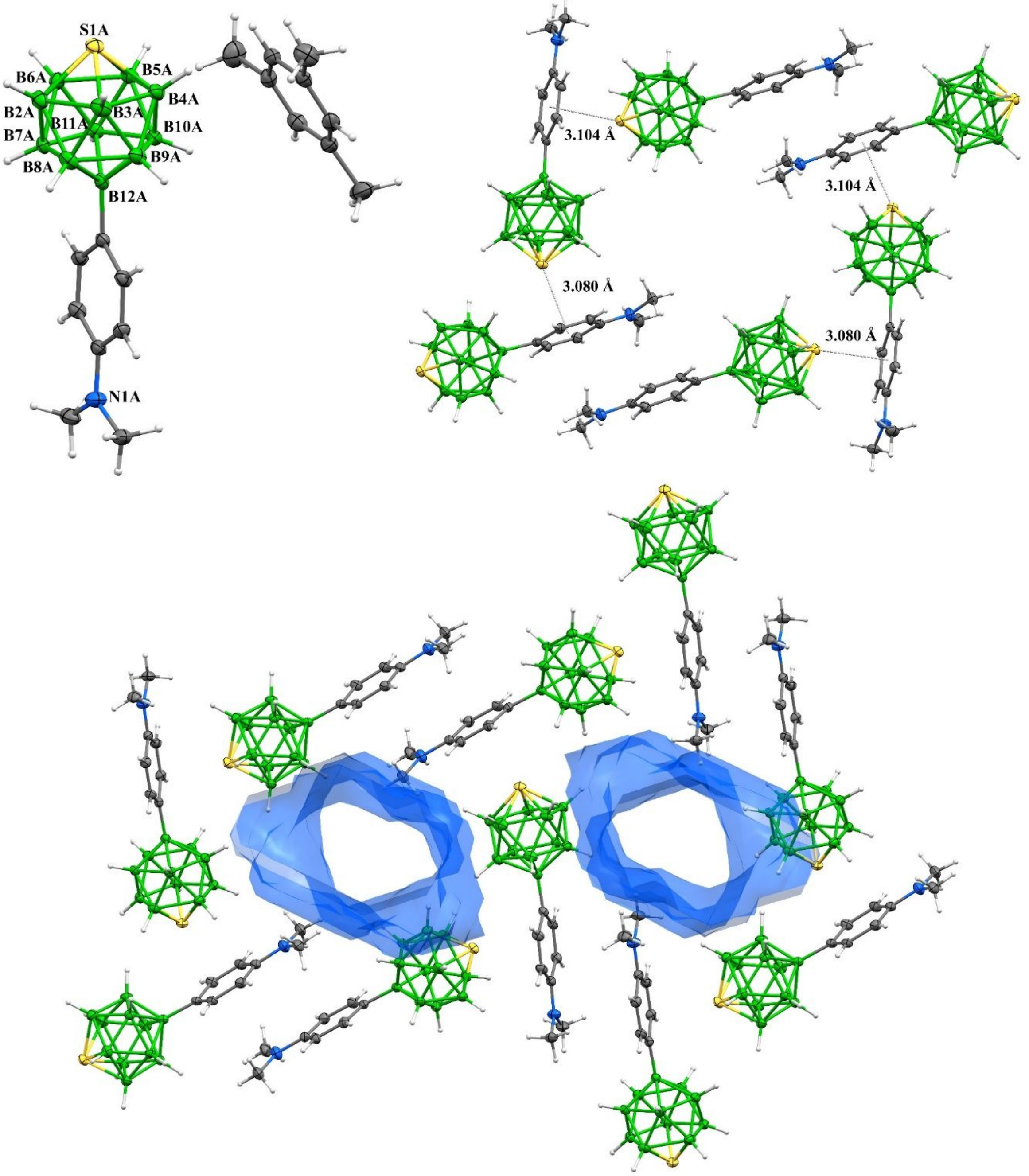

Figure S21: The molecular structure and fragment of crystal package of **5m** (top). Voids formed when solvent molecules are removed by SQUEZZE procedure[31] (750.58 Å^3 or 19.1 % of the unit cell volume) (bottom). Thermal ellipsoids are drawn with the 40% probability level.

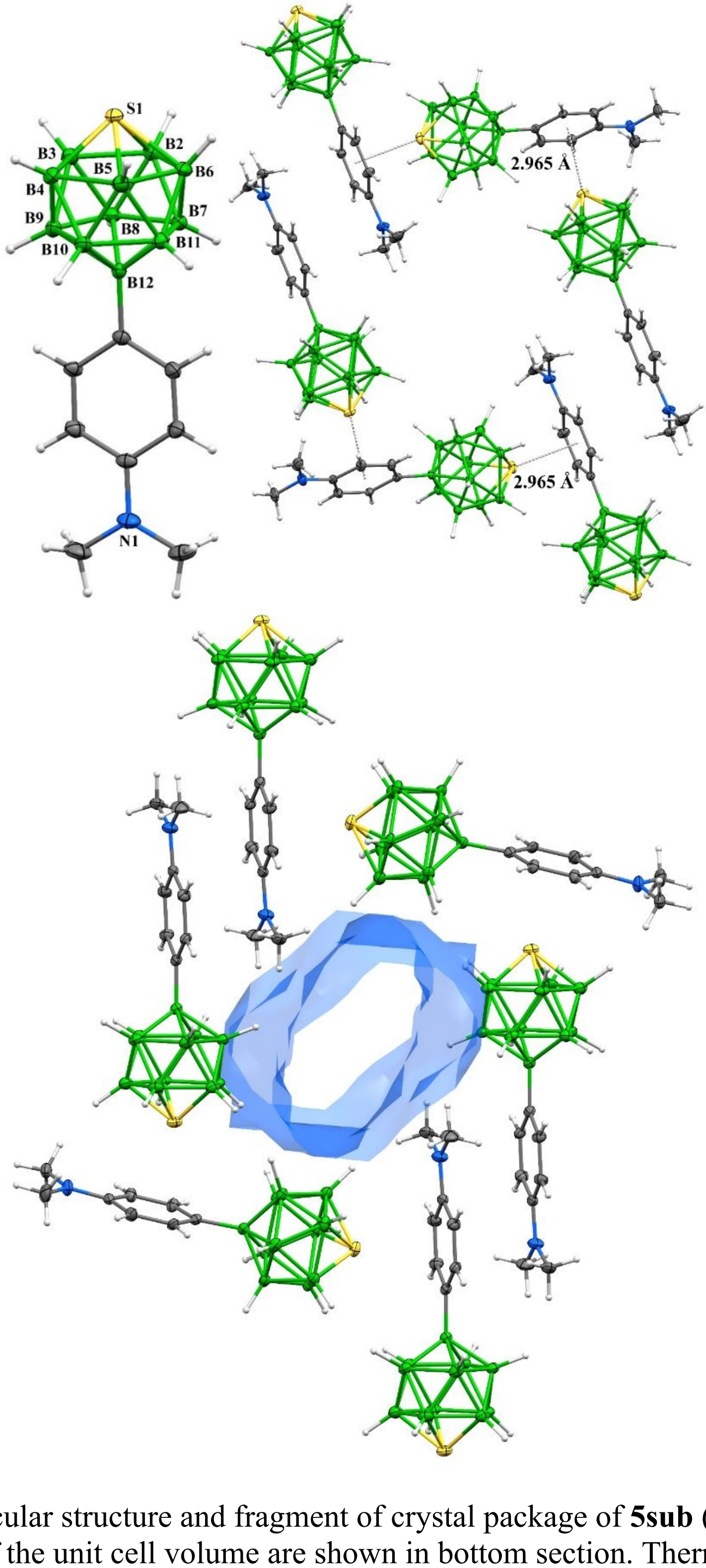

Figure S22: The molecular structure and fragment of crystal package of **5sub** (top). Voids of 389.04 Å^3 or 20.4 % of the unit cell volume are shown in bottom section. Thermal ellipsoids are drawn with the 40% probability level.

Table S2: Crystallographic data for compounds **2**, **3**, **4b** and **4h**.

**Complex CCDC nr.**	**2 2068574**	**3 2068575**	**4b 2068584**	**4h 2068577**
Empirical formula	$C_7H_{17}B_{11}OS$	$C_7H_{17}B_{11}S_2$	$C_{12}H_{19}B_{11}S$	$C_{12}H_{19}B_{11}S$
Formula weight	268.17	284.23	314.24	314.24
Crystal system	Monoclinic	Monoclinic	Orthorhombic	Monoclinic
Space group	$P2_1$/n	$P2_1$/c	$P2_12_12_1$	$P2_1$/c
Unit cell dimensions				
a[Å]	7.1638(3)	14.4565(18)	10.344(2)	8.0489(3)
b[Å]	18.7493(9)	7.3738(9)	12.8080(14)	15.6980(6)
c[Å]	10.8823(6)	28.920(4)	13.2502(12)	13.9182(5)
α[°]	90	90	90	90
β[°]	91.478(2)	90.344(4)	90	93.9782(15)
γ[°]	90	90	90	90
Volume [Å^3]	1461.18(12)	3082.8(7)	1755.5(4)	1754.35(11)
Z	4	8	4	4
Density (calculated) [Mg m^{-3}]	1.219	1.225	1.189	1.190
Absorption coefficient [mm^{-1}]	0.199	0.319	0.172	1.486
Max. and min. transmission	0.7456 and 0.6857	0.9582 and 0.5677	0.7456 and 0.6164	0.7536 and 0.6105
Crystal size [mm]	0.544 x 0.173 x 0.076	0.334 x 0.158 x 0.110	0.592 x 0.374 x 0.359	0.494 x 0.230 x 0.205
θ range for data collection [°]	2.164 to 27.516	2.532 to 24.997	2.498 to 27.506	4.251 to 72.464
Reflections collected / unique	42210 / 3358	45074 / 5326	14416 / 4027	22080 / 3471
R(int)	0.1109	0.0692	0.0511	0.0535
Data / restraints / parameters	3358 / 0 / 182	5326 / 32 / 369	4027 / 291 / 257	3471 / 0 / 217
Final R indices [I>2sigma(I)]	R_1 = 0.0524, wR_2 = 0.1241	R_1 = 0.1615, wR_2 = 0.3509	R_1 = 0.0415, wR_2 = 0.0872	R_1 = 0.0392, wR_2 = 0.1035
R indices (all data)	R_1 = 0.0774, wR_2 = 0.1346	R_1 = 0.1717, wR_2 = 0.3557	R_1 = 0.0539, wR_2 = 0.0920	R_1 = 0.0481, wR_2 = 0.1086
Goodness-of-fit on F^2	1.081	1.124	1.032	1.064
Largest diff. peak and hole [e/Å^3]	0.289 and –0.292	1.388 and –0.932	0.192 and –0.289	0.265 and –0.278

Table S3: Crystallographic data for compounds **5b**, **5cl**, **5cs** and **5d**.

Complex	**5b** **2068578**	**5cl** **2068586**	**5cs** **2068582**	**5d** **2068585**
Empirical formula	$C_{16}H_{40}B_{22}N_2S_2$ C_6H_6	$C_8H_{20}B_{11}NS$ $0.5\cdot CCl_4$	$C_8H_{20}B_{11}NS$ CS_2	$C_8H_{20}B_{11}NS$ $0.5\cdot C_{10}H_{18}$
Formula weight	640.55	358.12	357.35	350.34
Crystal system	Monoclinic	Triclinic	Monoclinic	Monoclinic
Space group	$P2_1$/c	P-1	$P2_1$/c	$P2_1$/c
Unit cell dimensions				
a[Å]	14.4500(8)	6.6626(4)	7.5570(3)	7.5692(4)
b[Å]	14.0288(7)	23.5030(15)	14.0024(6)	13.9922(8)
c[Å]	18.7716(10)	25.0440(14)	18.2250(8)	18.1347(10)
α[°]	90	91.828(2)	90	90
β[°]	98.316(4)	95.257(3)	97.275(2)	97.251(2)
γ[°]	90	95.418(3)	90	90
Volume [Å^3]	3765.3(3)	3884.5(4)	1912.97(14)	1905.28(18)
Z	4	8	4	4
Density (calculated) [Mg m^{-3}]	1.130	1.225	1.241	1.221
Absorption coefficient [mm^{-1}]	1.403	0.431	0.377	0.166
Max. and min. transmission	0.5461 and 0.2665	0.9009 and 0.8076	0.9281 and 0.8473	0.9420 and 0.9011
Crystal size [mm]	1.256 x 0.397 x 0.304	0.419 x 0.286 x 0.219	0.508 x 0.216 x 0.142	0.686 x 0.454 x 0.340
θ range for data collection [°]	3.091 to 70.485	2.452 to 25.500	2.682 to 25.994	2.264 to 25.996
Reflections collected / unique	36860 / 7124	57012 / 13033	47011 / 3754	38543 / 3735
R(int)	0.1439	0.0591	0.0924	0.0525
Data / restraints / parameters	7124 / 72 / 512	13033 / 280 / 1063	3754 / 13 / 268	3735 / 75 / 302
Final R indices [I>2sigma(I)]	R_1 = 0.1063, wR_2 = 0.2405	R_1 = 0.0918, wR_2 = 0.2475	R_1 = 0.0499, wR_2 = 0.1200	R_1 = 0.0489, wR_2 = 0.1332
R indices (all data)	R_1 = 0.1550, wR_2 = 0.2670	R_1 = 0.1100, wR_2 = 0.2613	R_1 = 0.0683, wR_2 = 0.1278	R_1 = 0.0599, wR_2 = 0.1399
Goodness-of-fit on F^2	1.060	1.023	1.054	1.066
Largest diff. peak and hole [e/Å^3]	0.725 and –0.543	1.706 and –1.240	0.527 and –0.585	0.449 and –0.684

Table S4: Crystallographic data for compounds **5e**, **5f**, **5h** and **5m**.

Complex	**5e** **2068580**	**5f** **2068579**	**5h** **2068576**	**5m** **2068583**
Empirical formula	$C_8H_{20}B_{11}NS$ $0.5 \cdot C_2H_5O$	$C_{16}H_{40}B_{22}N_2S_2$ C_6F_6	$C_8H_{20}B_{11}NS$ $0.25 \cdot C_6H_{14}$	$C_8H_{20}B_{11}NS$ $0.5 \cdot C_9H_{12}$
Formula weight	318.28	748.50	302.76	341.31
Crystal system	Monoclinic	Orthorhombic	Tetragonal	Monoclinic
Space group	$P2_1/c$	*F*dd2	*I*4/m	$P2_1/c$
Unit cell dimensions				
a[Å]	6.6678(4)	34.7127(11)	16.9662(13)	14.0095(4)
b[Å]	21.0495(12)	61.221(2)	16.9662(13)	22.1267(6)
c[Å]	13.8047(7)	7.3654(3)	12.3880(12)	13.4460(4)
α[°]	90	90	90	90
β[°]	99.450(2)	90	90	109.148(2)
γ[°]	90	90	90	90
Volume [Å^3]	1911.25(19)	15652.6(10)	3565.9(6)	3937.4(2)
Z	4	16	8	8
Density (calculated) [Mg m^{-3}]	1.106	1.270	1.128	1.152
Absorption coefficient [mm^{-1}]	0.161	1.655	0.168	1.369
Max. and min. transmission	0.7453 and 0.5955	0.7536 and 0.3904	0.9420 and 0.8416	0.7846 and 0.4398
Crystal size [mm]	0.582 x 0.402 x 0.379	0.210 x 0.200 x 0.180	0.496 x 0.494 x 0.370	0.448 x 0.218 x 0.216
θ range for data collection [°]	2.446 to 25.999	2.927 to 72.167	2.401 to 25.999	3.339 to 72.529
Reflections collected / unique	45884 / 3750	17898 / 6884	22404 / 1840	40456 / 7798
R(int)	0.0739	0.0473	0.0923	0.0782
Data / restraints / parameters	3750 / 68 / 268	6884 / 160 / 584	1840 / 20 / 156	7798 / 0 / 547
Final R indices [I>2sigma(I)]	R_1 = 0.0607, wR_2 = 0.1936	R1 = 0.0724, *w*R2 = 0.1872	R_1 = 0.0461, wR_2 = 0.1138	R_1 = 0.0525, wR_2 = 0.1253
R indices (all data)	R_1 = 0.0662, wR_2 = 0.1998	R1 = 0.0935, *w*R2 = 0.2059	R_1 = 0.0664, wR_2 = 0.1214	R_1 = 0.0762, wR_2 = 0.1375
Goodness-of-fit on F^2	1.098	1.021	1.070	1.043
Largest diff. peak and hole [e/Å^3]	1.450 and –0.468	0.649 and –0.380	0.342 and –0.382	0.264 and –0.341

Table S5: Crystallographic data for compound **5sub**.

Complex	**5sub 2068581**
Empirical formula	$C_8H_{20}B_{11}NS$
Formula weight	281.22
Crystal system	Monoclinic
Space group	$P2_1$/c
Unit cell dimensions	
a[Å]	6.6729(6)
b[Å]	20.9895(17)
c[Å]	13.8388(12)
α[°]	90
β[°]	99.771(3)
γ[°]	90
Volume [$Å^3$]	1910.2(3)
Z	4
Density (calculated) [Mg m^{-3}]	0.978
Absorption coefficient [mm^{-1}]	0.153
Max. and min. transmission	0.9705 and 0.7493
Crystal size [mm]	0.341 x 0.325 x 0.230
θ range for data collection [°]	2.449 to 25.999
Reflections collected / unique	18344 / 3684
R(int)	0.0556
Data / restraints / parameters	3684 / 0 / 232
Final R indices [I>2sigma(I)]	R_1 = 0.0931, wR_2 = 0.2571
R indices (all data)	R_1 = 0.1051, wR_2 = 0.2657
Goodness-of-fit on F^2	1.127
Largest diff. peak and hole [e/$Å^3$]	1.091 and –0.521

Binding modes in X-ray crystal structures

The previously published thiaborane **1** forms S•••π chalcogen bond with the separation of 3.24 Å (Figure S23). The axes (S-B12) of the two molecules of **1** are not perpendicular (B12-S-Ph angle is 155°), which agrees with the prediction of nonlinearity of the chalcogen bond of the thiaboranes.[16] We modelled a hypothetical chalcogen-bonded dimer of **1** with the perpendicular arrangement for comparison, and this dimer had comparable stability as the motif found in the crystal structure (Figure S24). The strength of the chalcogen bond decreases when the S1-B12-Cg angle is smaller than 150°. The chalcogen bond of **1** has highly negative interaction energy values of –9.6 kcal/mol at the SAPT0/jun-cc-pVDZa level (Table S6), which is in reasonable agreement with the benchmark CCSD(T) value of –8.6 kcal/mol.[16] The chalcogen bond is dominated by the dispersion and electrostatic contribution, which form 59 and 31 %, respectively, of the sum of the attractive terms in the SAPT decomposition. The chalcogen bond is associated with the most negative interaction energy value in the crystal structure of **1**.[16]

In **2**, S•••π contacts were not found in the crystal structure. Instead, the S•••H-B chalcogen bond between the S atom and the hydridic B-H vertex is formed with the S•••H separation of 2.92 Å. Additionally, S•••O contact longer than $\sum r_{vdW}$ is also presented in the crystal structure of **2**. Although the interaction energies of the S•••H-B and S•••O contacts (–4.8 and –3.7 kcal/mol, respectively) are less favourable than that of the hypothetical S•••π chalcogen bond of **2** (–10.6 kcal/mol, see Table S6), their sum is only slightly smaller. Additionally, the crystal packing enables the formation of favourable π•••π stacking with ΔE value of –8.1 kcal/mol.

The S•••π chalcogen bond found in the crystal structure of **3**, is shorter than the chalcogen bond of **1**, and its interaction energy is more negative (–12.0 kcal/mol). The nature of the chalcogen bond is, however, very similar, i.e. dispersion and electrostatic contribution from about 60 and 30 %, respectively, of the sum of attractive SAPT0 terms.

The biphenyl derivative **4** has two eligible phenyl rings that can form the S•••π chalcogen bond. The chalcogen bond with the terminal phenyl ring is shorter and more perpendicular. It might thus be surprising that the chalcogen bonding motif of the terminal ring has less negative interaction energy than the chalcogen bond with the bridging ring. This can, however, be rationalized by the SAPT decomposition, which shows that while the chalcogen bond with the terminal ring has slightly more favourable electrostatic stabilisation, the chalcogen with the bridging ring has considerably more favourable dispersion contribution (see Table S6).

A very short chalcogen bond of compound **5** of 3.07 Å (i.e. 88 % of the sum of r_{vdW} of C and S atoms) should be noticed. In agreement with such a shorter S•••π separation, the chalcogen bond of **5** has the most negative interaction energy of the studied compounds (ΔE of –12.5 kcal/mol). The nature of this chalcogen bond is, however, very similar to that of **1**, i.e. dispersion and electrostatic form about 60 and 30 %, respectively, of the sum of attractive SAPT0 terms.

Table S6: SAPT0 energies for the dimers taken from the X-ray crystal structures. Hydrogen and boron atoms were optimized by the DFT-D3/BLYP/DZVP method. Energies in kcal/mol. The relative values in parentheses show the contribution to the sum of all the attractive terms.

	SAPT0/jun-cc-pVDZa				
	total	E_{disp}	E_{elec}	E_{ind}	E_{exch}
12-Ph-*closo*-1-$SB_{11}H_{10}$ (**1**) S•••π chalcogen bond	–9.64	–12.06 (59%)	–6.27 (31%)	–1.96 (10%)	10.65
12-(4-OMe-C_6H_4)-*closo*-1-$SB_{11}H_{10}$ (**2**) S•••O chalcogen bond	–3.90	–5.09 (65%)	–2.02 (26 %)	–0.67 (9%)	3.89
12-(4-OMe-C_6H_4)-*closo*-1-$SB_{11}H_{10}$ (**2**) π•••π stacking	–8.10	–12.07 (68%)	–4.17 (24%)	–1.51 (9%)	9.65
12-(4-OMe-C_6H_4)-*closo*-1-$SB_{11}H_{10}$ (**2**) S•••HB	–4.68	–6.57 (72%)	–1.92 (21%)	–0.64 (7%)	4.44
12-(4-OMe-C_6H_4)-*closo*-1-$SB_{11}H_{10}$ (**2**) hypothetical S•••π not found in X-ray	–10.62	–12.79 (61%)	–6.25 (30%)	–2.07 (10%)	10.49
12-(4-SMe-C_6H_4)-*closo*-1-$SB_{11}H_{10}$ (**3**) S•••π chalcogen bond	–11.98	–16.55 (60%)	–8.36 (30%)	–2.80 (10%)	15.72
12-(4-Ph-C_6H_4)-*closo*-1-$SB_{11}H_{10}$ (**4**) S•••π and S•••HB chalcogen bonds (with S•••HB and S•••C_6H_4 (**bridging**) intereractions)	–10.62	–15.27 (65%)	–6.16 (26%)	–1.93 (8%)	12.74
12-(4-Ph-C_6H_4)-*closo*-1-$SB_{11}H_{10}$ (**4**) S•••π chalcogen bond (with S•••C_6H_5 (**terminal**) intereractions)	–8.73	–11.15 (57%)	–6.22 (32%)	–2.06 (11%)	10.70
12-(4-NMe_2-C_6H_4)-*closo*-1-$SB_{11}H_{10}$ (**5**) S•••π chalcogen bond	–12.47	–14.95 (59%)	–7.60 (30%)	–2.73 (11%)	12.81

[a] Interaction energies were decomposed into dispersion (E_{disp}), electrostatic (E_{elec}), exchange (E_{exch}) and induction (E_{ind})contributions. The relative values in parentheses express the contribution to the sum of all attractive energy terms of SAPT0.

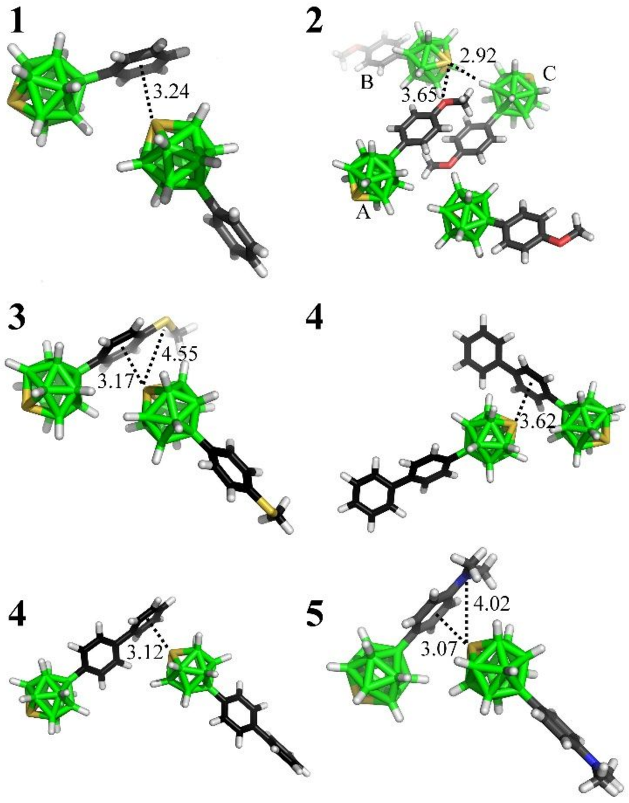

Figure S23: Crystal packing of **1**, **2**, **3**, **4** and **5**. Distances in Å. Positions of H and B atoms optimized at the DFT-D3/BLYP/DZVP level.

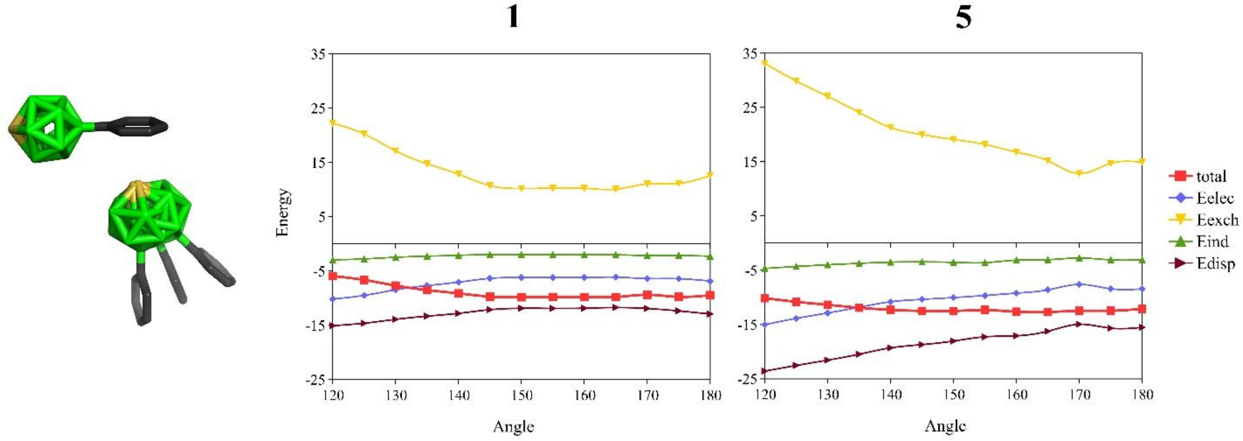

Figure S24: SAPT0 energies plotted against the B12-S1-Cg(second molecule) angle for the chalcogen-bonded dimers of **1** and **5**. Angles and energies are in degrees and kcal/mol, respectively. The geometries of the dimers were optimized by the DFT-D3/BLYP/DZVP method. The angle was restrained during the optimization.

Dipole moments and electrostatic potential (ESP) molecular surfaces

The *closo*-$SB_{11}H_{11}$ molecule has very large dipole moment of 3.6 D as found experimentally.[1] The dipole moment can be significantly modulated by *exo*-substitutions, e.g. 12-Cl-*closo*-1-$SB_{11}H_{10}$ has dipole moment of 5.5 D (error of 0.2 D).[1] The same computational methodology was used to estimate dipole moment for the compounds under scrutiny (Table S7). The dipole moment of **2** – **4** were similar to the parent *closo*-$SB_{11}H_{11}$. However, the NMe_2 group in **5** significantly decreased the dipole moment value to 1.0 D. Another tool for the evaluation of possible contacts is the electrostatic potential of the molecular surface (ESP) and interaction energy. For **1**–**5**, the calculated maximum and minimum values of the ESP ($V_{S,max}$ located on the sulfur atom of ~ 25 kcal/mol, and $V_{S,min}$ phenyl ring of ~ −26 kcal/mol) as well as the interaction energies (dimers taken from the crystal structures ~ −11 kcal/mol) show the same trend (±3 kcal/mol, Fig. 6, Table S7) in the whole series. Additionally, the lone pairs of S and O are more negative than the π ring (Table S7).

Table S7: Dipole moments and maximal and minimal values of the ESP of the molecular surface ($V_{S,max}$ and $V_{S,min}$, respectively) computed at the HF/cc-pVDZ level. Dipole moment in D and ESP in kcal/mol.

Molecule	Dipole moment	$V_{S,max}$ (S)	$V_{S,min}$ (ring)	$V_{S,min}$ (heteroatom)
closo-$SB_{11}H_{11}$	3.4 (exp. 3.6)	28.2	--	--
12-Cl-*closo*-1-$SB_{11}H_{10}$	5.3 (exp. 5.5)	30.7	--	--
12-Ph-*closo*-1-$SB_{11}H_{10}$ (**1**)	3.3	26.7	–25.1	--
12-(4-OMe-C_6H_4)-*closo*-1-$SB_{11}H_{10}$ (**2**)	3.2	25.8	–24.8	–30.9 (O)
12-(4-SMe-C_6H_4)-*closo*-1-$SB_{11}H_{10}$ (**3**)	3.9	27.1	–23.6	–24.4
12-(4-Ph-C_6H_4)-*closo*-1-$SB_{11}H_{10}$ (**4**)	3.4	27.0	–23.7	--
12-(4-NMe_2-C_6H_4)-*closo*-1-$SB_{11}H_{10}$ (**5**)	1.0	24.0	–29.0	–22.1 (N)

Interactions with CO_2

The ESP molecular surface of CO_2 (Fig. S25) can be characterized by the areas of a negative ESP on the oxygen atoms ($V_{S,min}$ of –16.5 kcal/mol) and by the ring of highly positive ESP around the carbon atom ($V_{S,max}$ of –33.9 kcal/mol). CO_2 can thus be expected to form various favorable interactions with crystal structures of **5**, see Figures S26 - S28. Specifically, the carbon atom of CO_2 can favorably interact with hydridic BH vertices, π electrons of the phenyl ring, and the nitrogen atom. Moreover, the oxygen atom of CO_2 can interact with the σ-hole of the S atom. In order to get deeper insight into these interactions, we have computed interaction energies of CO_2 with the fragments of **5**, a single molecule of **5,** and models of crystal structures of **5** at the DFT-D3 level, see Figure S29. The obtained results confirmed a strong interaction between the N atom of **5** and CO_2 (ΔE of –4.2 kcal/mol, the C•••N separation of 3.0 Å). This interaction energy is comparable to the interaction with NMe_2, which has an interaction energy of –5.0 kcal/mol. The other modeled interaction motifs are less favorable. The single C•••H-B tetrel bond has interaction energy of –1.2 kcal/mol, the motif combining C•••H-B tetrel bonds and B-S•••O chalcogen bond has interaction energy of –2.1 kcal/mol, and the stacking complex of CO_2 with the phenyl ring has interaction energy of –2.1 kcal/mol. Considering the model of crystal structures, the most favorable binding motif was found for the smaller cavity (**5h**), where the carbon atom of CO_2 can interact simultaneously with four nitrogen atoms of **5** (ΔE of –9.2 kcal/mol, four C•••N contacts of 3.8 Å). CO_2 can also interact with BH vertices of four thiaborane cages in this model, but this motif has less negative interaction energy of –6.1 kcal/mol. In the case of the large cavity (**5e**, **5sub**) in the crystal structure of **5**, the N atoms are not sterically accessible, and the computed interaction energy is thus less favorable. The visualization of selective and reversible adsorption of CO_2 is demonstrated in Movie S1.

Movie S1.

Visualization of selective and reversible adsorption of CO_2 to the material of $\mathbf{5h_{70}}$

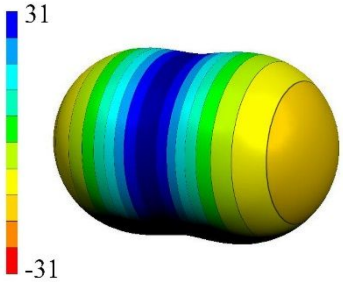

Figure S25: Computed ESP molecular surfaces of CO_2. The ESP color ranges from –31.4 (red) to 31.4 (blue) kcal/mol.

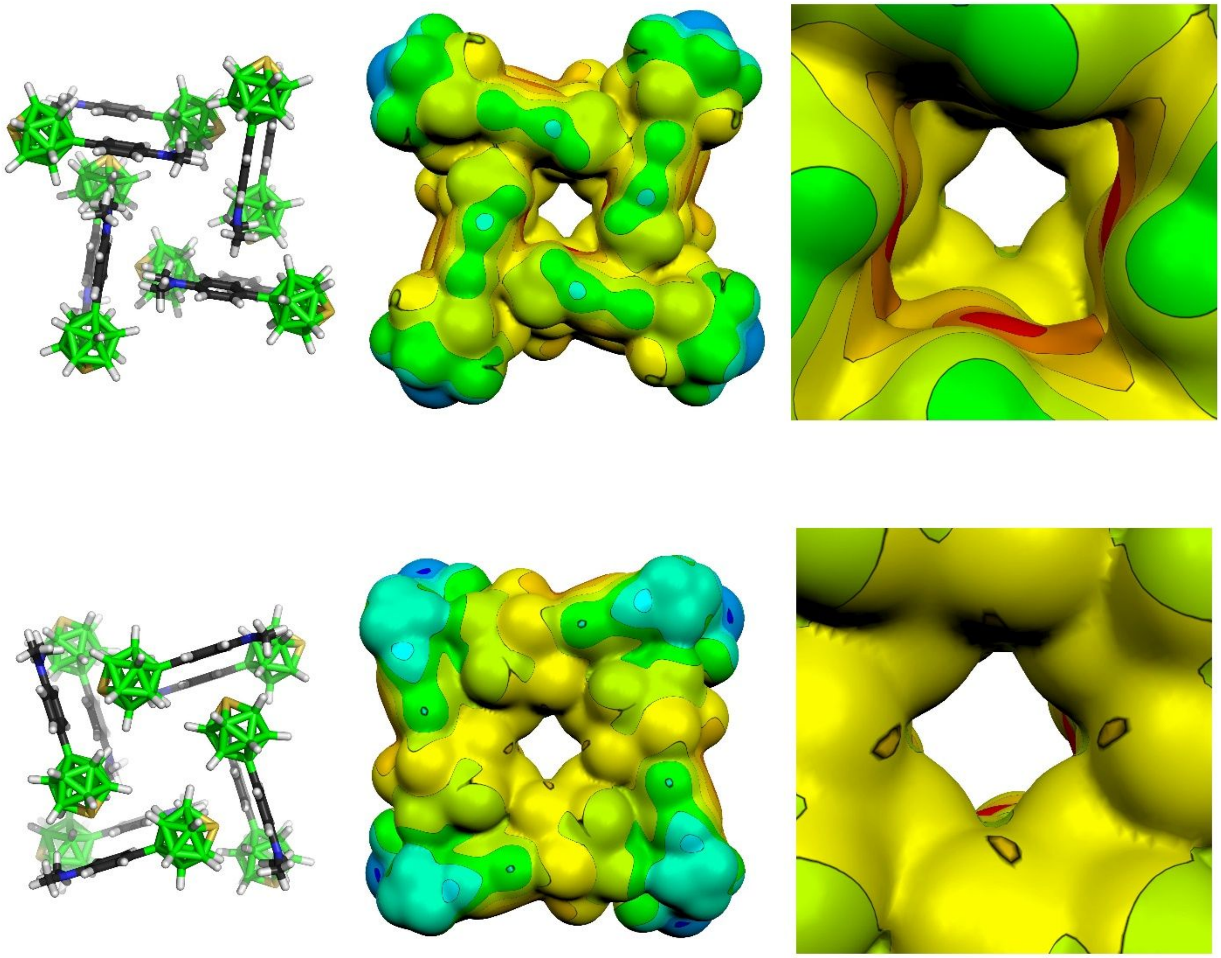

Figure S26: Computed ESP molecular surfaces of **5h** (smaller cavity) in two different orientations. The ESP colour range in kcal/mol.

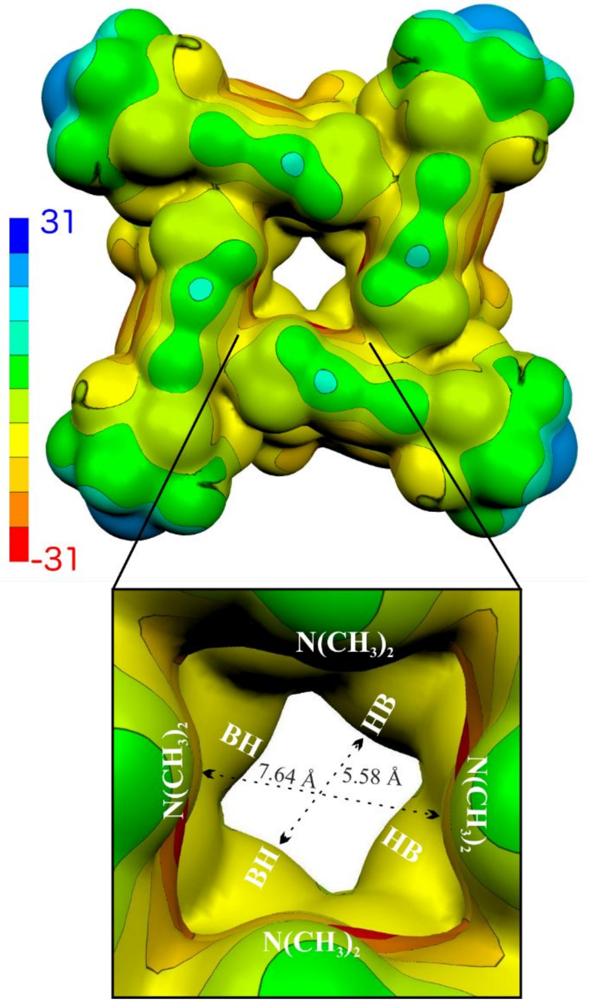

Figure S27: Computed ESP molecular surfaces of **5h** (smaller cavity) with proportions. The ESP color range in kcal/mol.

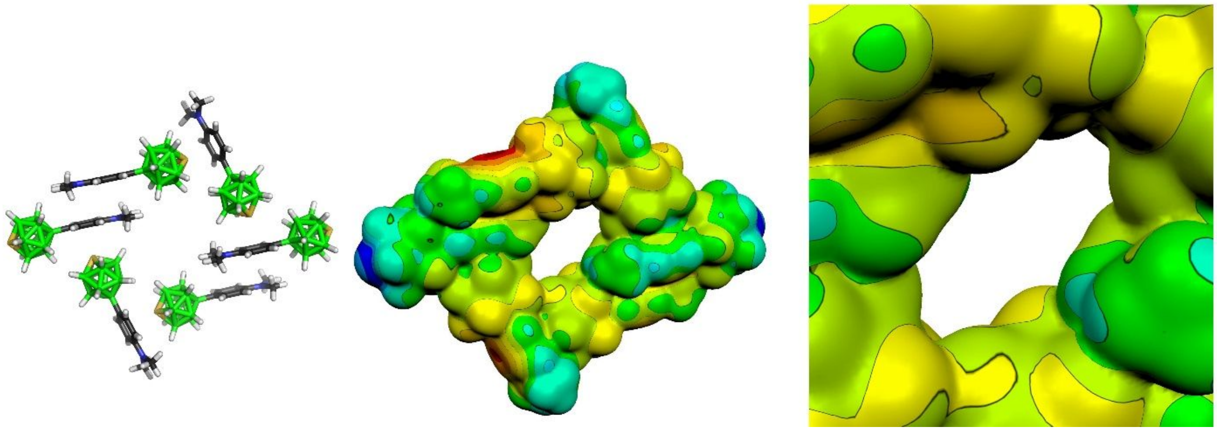

Figure S28: Computed ESP molecular surfaces of **5e** (larger cavity). The ESP color range in kcal/mol.

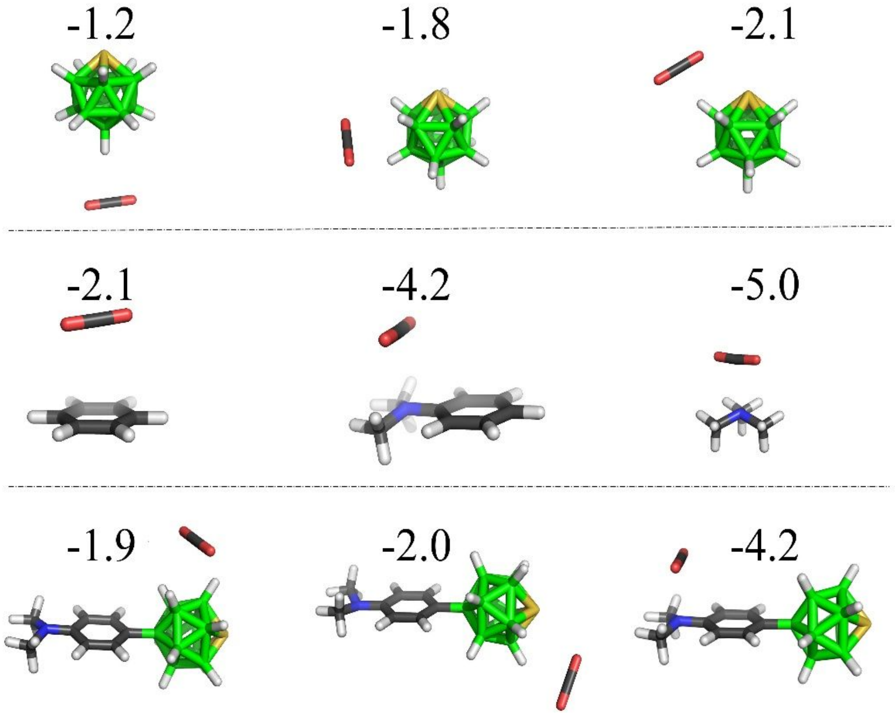

Figure S29: Computed DFT-D3/BLYP/DZVP interaction energy (ΔE) values in kcal/mol.

Adsorption data

Textural properties (specific surface area and porosity) were investigated by the physisorption of dinitrogen at the temperature of liquid nitrogen. Obtained adsorption isotherms are reported in Fig. S30. Sample **5h** degassed at room temperature exhibits very low adsorption capability and isotherm resembles adsorption isotherm of type III according to IUPAC nomenclature. The specific surface area was estimated to be 0.5 m^2/g by the application of the BET theory. It indicates that the material is absolutely non-porous and has large particles, or the pores are still blocked by solvent, which cannot be removed by outgassing at room temperature. To check this, the sample was degassed at the elevated temperature of 70°C (**5h$_{70}$**). The amount of adsorbed gas increased significantly. The specific surface area of **5h$_{70}$** sample is 16.6 m^2/g. Sample **5e,** crystallized in ether, exhibits a specific surface area of 11.5 m^2/g even after outgassing at room temperature due to the easier evaporation of solvent from the sample. Samples **5h$_{70}$** and **5e** were also subjected to adsorption of CO_2 at 273 K (see Fig. S31). CO_2 adsorption isotherms indicate the existence of micropores with sizes between 0.6 and 0.8 nm for **5h$_{70}$** and 0.45 and 0.6 nm for **5e** sample. The specific surface area derived from CO_2 adsorption isotherms is 309 and 105 m^2/g for **5h$_{70}$** and **5e**, respectively. The discrepancy between N_2 and CO_2 probed surface areas can be caused by the differences in kinetic diameters of both molecules (CO_2 is smaller) and by the diffusivity of molecules into pores.

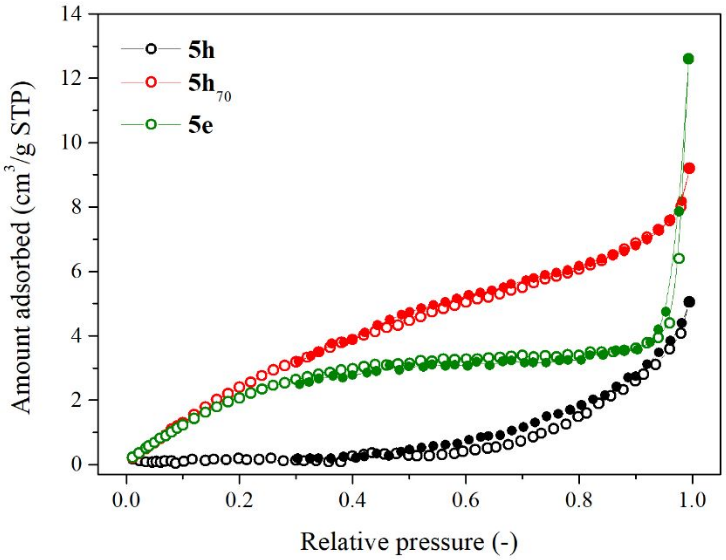

Figure S30: N_2 adsorption isotherms of **5h**, **5h$_{70}$** and **5e** measured at liquid nitrogen bath.

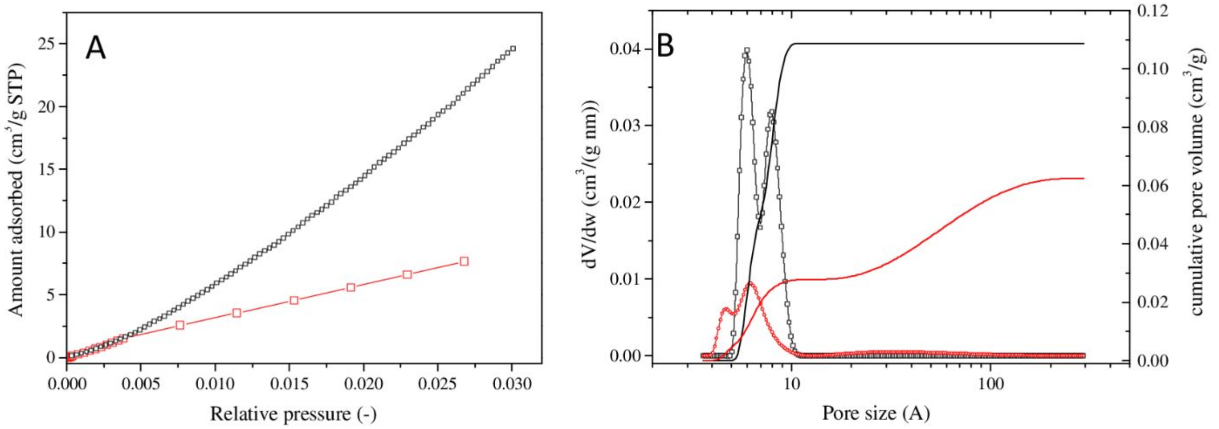

Figure S31: Adsorption isotherms of CO_2 at 273K on $\mathbf{5h_{70}}$ (black) and **5e** (red) (A) and pore size distribution resulted from NL DFT evaluation (B).

Adsorption isotherms of methane, carbon monoxide, and carbon dioxide on $\mathbf{5h_{70}}$ sample at 298 K reported in Fig. 2 of the main text were used for estimation of adsorption selectivity in binary mixture by means of IAS theory. The adsorption selectivity for the separation of a binary mixture is defined by the following equation:

$$\alpha_{i,j} = \frac{x_i / y_i}{x_j / y_j} = \frac{p_j^0}{p_i^0}$$

where x_i is an i-th component molar fraction in the adsorbed phase, y_i is i-th component molar fraction in the gas phase, and p_i^0 is the pressure of pure i-th component necessary for achieving the spreading pressure in the adsorbed phase as is considered binary mixture predicted by ideal adsorbed solution theory (IAST). In general, IAST cannot be solved in close form and solution must be found numerically. For this purpose, we use our own software employing a Scilab package. The pure component adsorption isotherms (Fig. 9 in the main text) were fitted with Sips (Langmuir-Freundlich) model[33] for the purpose of IAST selectivity calculations.

$$V_{ads} = V_{\max} \frac{(bp)^{1/n}}{1 + (bp)^{1/n}}$$

where p (in mbar) is the pressure of the bulk gas at equilibrium with adsorbed phase, b (in $mbar^{-1}$) is the affinity coefficient, V_{max} (in cm^3/g STP) is the saturation capacity of the adsorbent, V_{ads} (in cm^3/g STP) is amount adsorbed and n is an empirical constant.

Sips adsorption isotherm allows us to derive an analytic expression for the calculation of adsorption potential *z* as a function of pressure p^0_i and the analytic expression for its inversion $p^0_i(z)$.

$$z = \frac{\phi}{RT} = \frac{\phi_i^0}{RT} = -\int_0^{p_i^0} \frac{V_i(p_i)}{p_i} dp_i = -V_{\max,i} \cdot n \cdot \ln\left(1 + \left(bp_i^0\right)^{\frac{1}{n}}\right)$$

Obtained expressions can be used straightforwardly for the numeric solution of unique *z* value for which the following equation holds.

$$\sum_{i=1}^{N} \frac{p_i}{p_i^0(z)} - 1 = 0$$

where p_i is the true partial pressure of components in the mixture. The calculation scheme was described in more detail in refs. 34 and 35.

Adsorption data obtained on **5h$_{70}$** sample at 298K fitted by Sips equation are shown in Fig. S32. The obtained fitting parameters were adopted for the calculation of adsorption selectivity by the IAST method. The IAST calculation was performed for 298K in the range of equilibrium pressures up to 1000 mbar for the binary mixture CO_2/CH_4, CO_2/CO, and CH_4/CO.

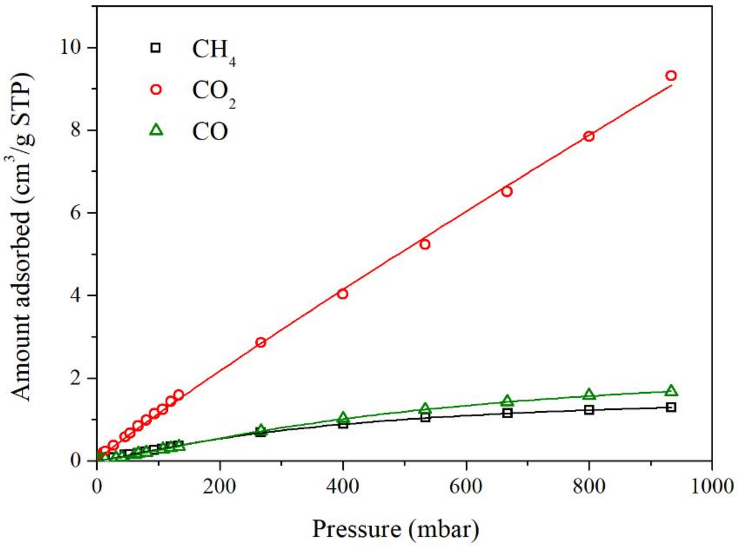

Figure S32: Adsorption isotherms of methane, CO and CO_2 at 298 K on **5h$_{70}$** sample. Points – experimental data, lines – Sips fit.

As can be seen from the isotherms themselves, CO_2 is preferentially adsorbed over CO and CH_4 on the **5h$_{70}$** sample over the entire pressure range considered. Its separation selectivity in an equimolar mixture of CO_2 with methane is around five at pressures below 200 mbar, then it begins to increase and reaches values around 20 at atmospheric pressure (Fig. S33 top). Single component adsorption isotherms of CO_2 and methane and isotherms predicted by IAST for equimolar mixture as a function of total pressure are depicted in Fig. S33 bottom. The preferential adsorption of CO_2 is very probably caused mainly by steric hindrances for methane diffusion since the kinetic diameter of methane is distinctly larger compared to carbon dioxide (3.3 vs 3.8 Å[33]). The CO_2 adsorption is slightly affected by the presence of methane in the mixture compared to the single-component isotherm. This slight decrease in the amount adsorbed of CO_2 indicates some competition in the occupation of the adsorption sites. The selectivity in the binary mixture of CO_2/CO is very similar with only exception of less steep increase with the pressure, thus reaching the selectivity of 10.5 at atmospheric pressure. It can also be explained by differences in diffusivities; the size of CO is similar to methane (3.76 Å). In the case of the CH_4/CO mixture, the adsorbent is almost unselective with the adsorption selectivity equal to 1 ± 0.1 in the whole pressure range. It is caused by the fact that both molecules probably cannot diffuse into material pores and adsorb only on the external surface. In addition, both molecules probably interact only by nonspecific dispersive interactions, and thus none of these components is preferred in adsorption.

Separation of CO_2 from methane is of great interest since carbon dioxide is often found as an impurity in landfill gas and natural gas.[34-36] The presence of carbon dioxide in natural gas can

cause corrosion of the pipelines, decreases the energy content of the gas, and increases the gas volume for transportation. Adsorptive separation is one of the most promising technologies for carbon dioxide removal. Most studies of CO_2/CH_4 separation have focused on zeolites, carbon-based materials, or MOFs. Typical adsorbent selectivity is in the range from 2 to 10. Our material exhibits selectivity at the top of the scale. However, it should be noted that many zeolitic adsorbents or MOFs exhibited significantly higher adsorption capacity. Some examples of adsorbents and their adsorption behaviour parameters are summarized in Table S8.

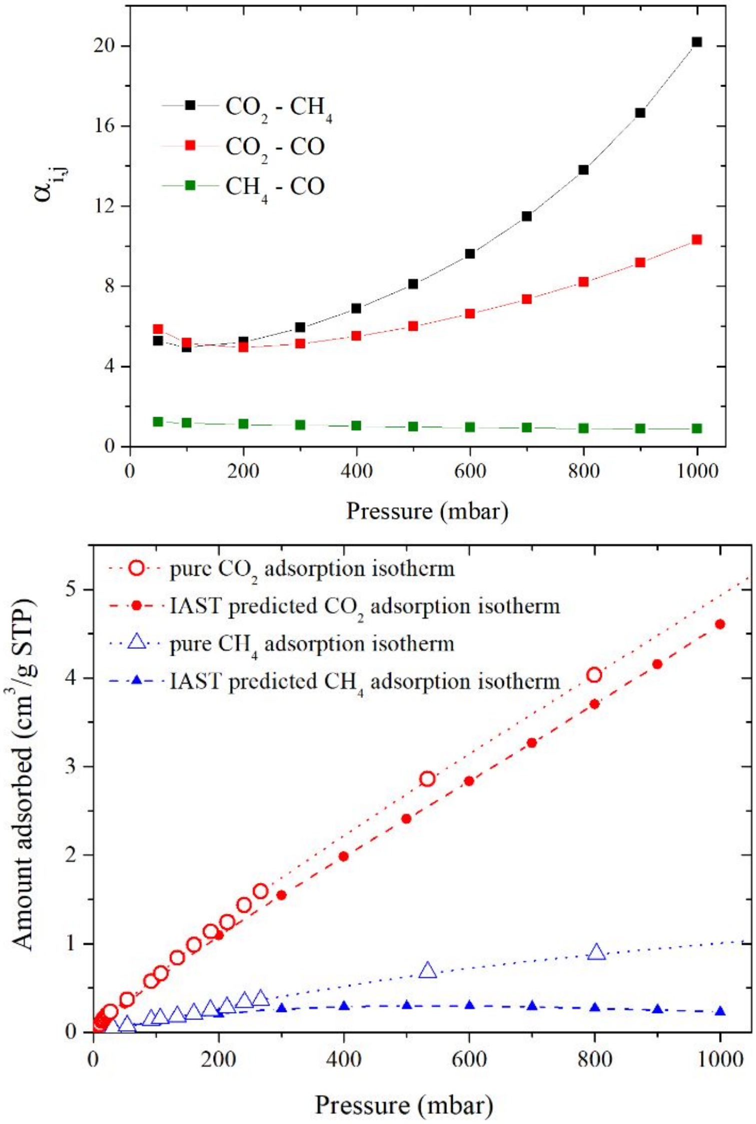

Figure S33: Comparison of adsorption isotherms for CO_2 (red) and CH_4 (blue) as pure-components (empty symbols and dotted lines) and in 50:50 CO_2/CH_4 mixtures (full symbols, dashed lines) on **5h$_{70}$**. Pure component isotherms are plotted with respect to partial pressure of the component in the mixture.

Table S8: Adsorption characteristics of the selected adsorbents

material	CO_2/CH_4 selectivity	Adsorption capacity of CO_2 (mmol/g)	conditions	reference
**5h$_{70}$**	20.3	0.45	CO_2:CH_4 =50:50, 298K, 1 atm	This work
MOF-5	2		CO_2:CH_4 =50:50, 298K, 1 atm	37
Cu-BTC	6	4.2	CO_2:CH_4 =50:50, 298K, 1 atm	38
MAF-X7	12.4	6.5	CO_2:CH_4 =50:50, 298K, 1 atm	39
silicalite	2.5		CO_2:CH_4 =50:50, 300K, 1 atm	40
JLU-Liu46	6.5	4.6	CO_2:CH_4 =5:95, 298K, 1 atm	41
JLU-Liu47	7.3	4.8	CO_2:CH_4 =5:95, 298K, 1 atm	41
Li-ZSM-25	66.9	1.9	CO_2:CH_4 =50:50, 303K, 2 atm	42
Aminated Cu-BTC-graphite oxide	9.6	13.4	CO_2:CH_4 =50:50, 298K, 15 atm	43
GO@MOF-505	8.6	3.9	298K, 1 atm	44
GrO@MIL-101	32	22.4	298K, 25 atm	45
GrO@Cu-BTC	14	8.19	273K, 1 atm	46
ZIF-78	10	2.6	CO_2:CH_4 =50:50, 298K, 1 atm	47
CAU-1	28	7.5	CO_2:CH_4 =50:50, 273K, 1 atm	48
MIL-53(Al)	7	1.3	CO_2:CH_4 =50:50, 303K, 1 atm	49
Na-MOR	1.9	2.2	308K, 1 atm	50
Na-BEA	4.2	2.6	CO_2:CH_4 =50:50, 273K, 1 atm	51
MOF-199	4.4	0.8	CO_2:CH_4 =50:50, 298 K, 1 atm	52
LTA-SIO_2	4.5	1.1	CO_2:CH_4 =50:50, 303K, 1 atm	53
IRH-3	17	2.5	CO_2:CH_4 =50:50, 298K, 1 atm	54
HKUST-1	7	11.4	CO_2:CH_4 =50:50, 273K, 1 atm	55

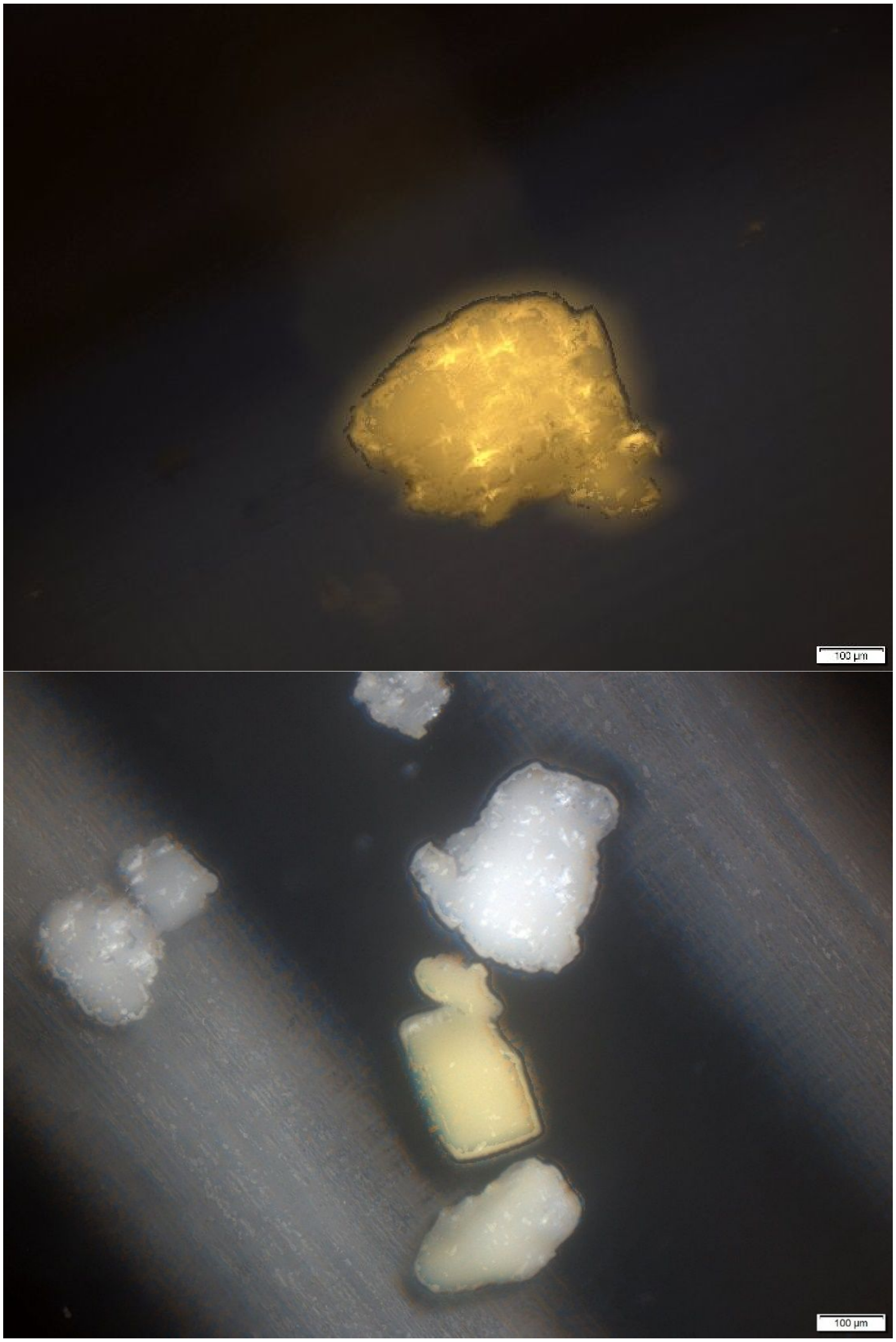

Figure S34: Upper figure: optical microscope snap of the material **5h** after room temperature evacuation in the capillary. Lower figure: the material after adsorption of CO_2 (1bar) in the capillary.

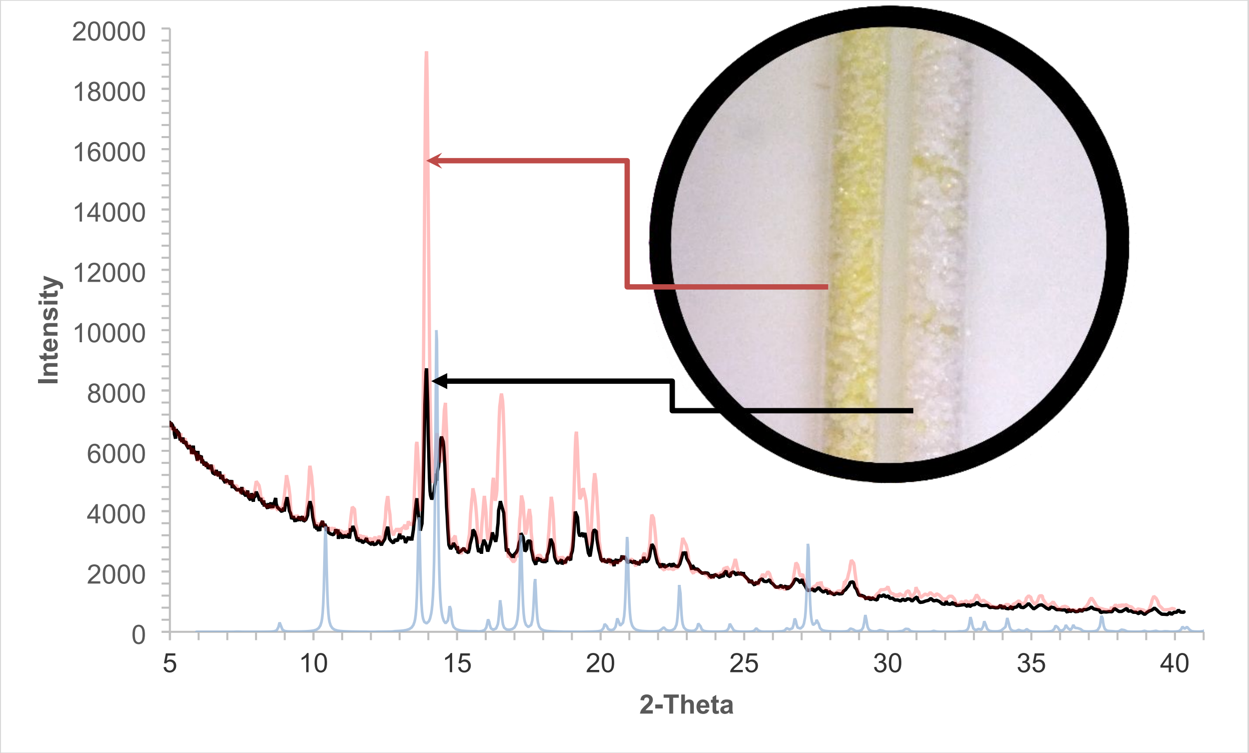

Figure S35: Powder diffractograms of **5h**. Red semi-transparent trace – yellow crystals; the material **5h** after room temperature evacuation in 0.5 mm capillary. Black trace – white crystals; the material **5h** after adsorption of CO_2 (1bar) in 0.5 mm capillary. Blue semi-transparent trace; the simulated pattern from sc-XRD measurement of **5h**.

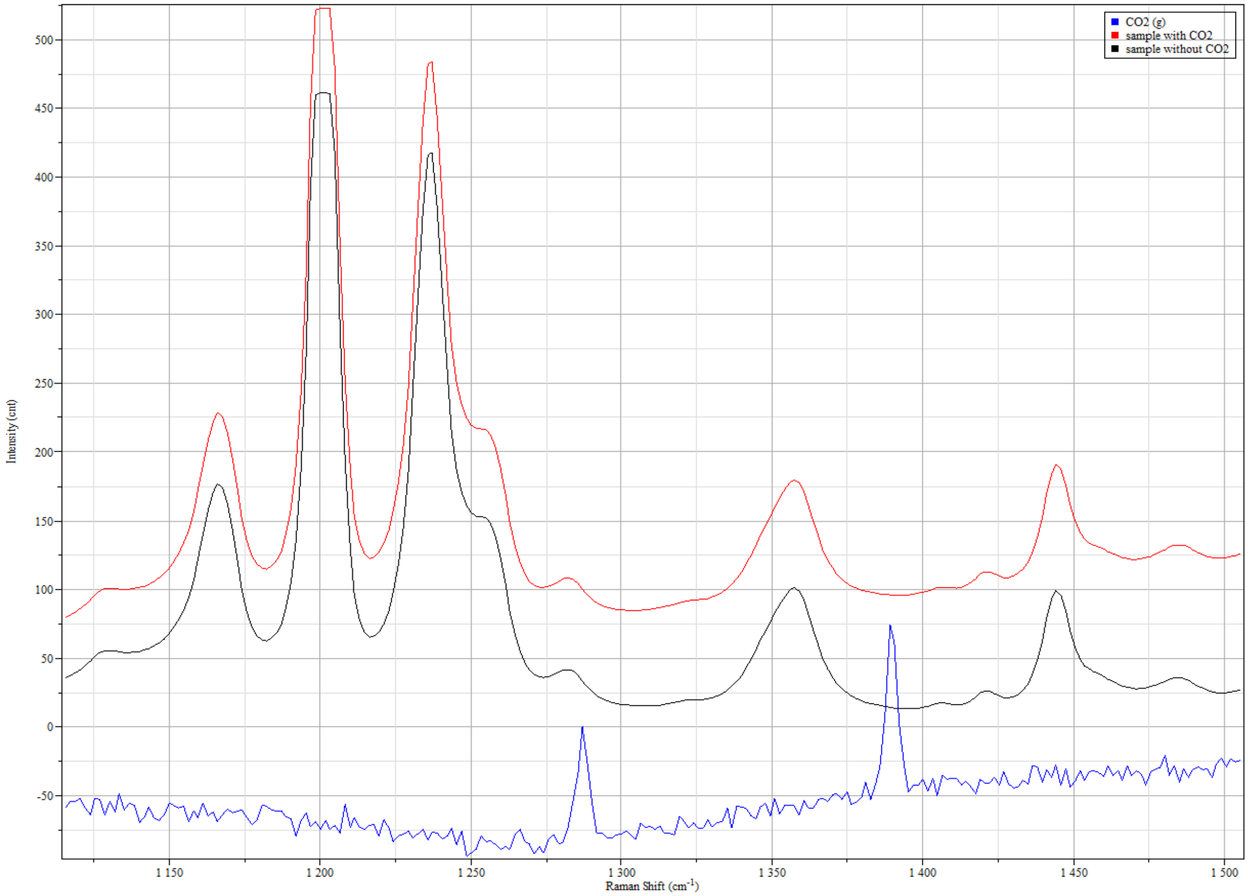

Figure S36: Raman spectra of **5h** (Labram HR, 532 nm laser, grating 600, objective 10x). Red trace; the material **5h** after room temperature evacuation in the capillary. Black trace the material **5h** after adsoption of CO_2 (1bar) in capillary (2s excitation, 1500 scans). Blue trace; the vapour phase over the crystals – signals belong to CO_2 (1s excitation, 10 scans)

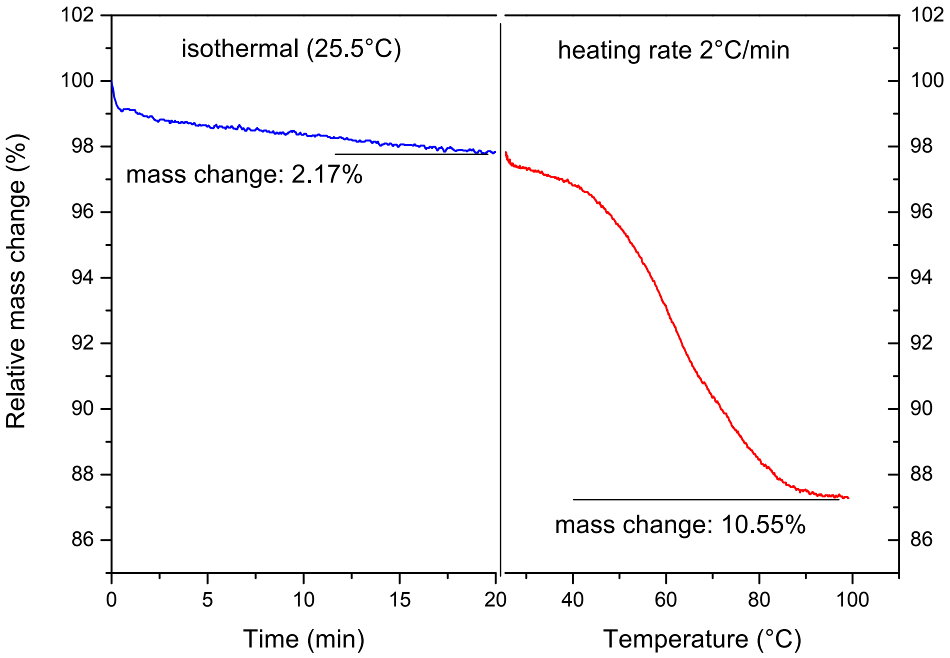

Figure S37: TG of **5h**; the TG experiment was conducted in the flow of dinitrogen (F = 50 ml/min). At first, the sample was flushed at a constant temperature 25.5 °C for 20 min. The mass slowly decreased by 2.17 % during isothermal period. Subsequently, the crucible with the sample was heated at a heating rate of 2°C/min. The mass of samples started to decrease and level-off at 87.28 % of the original mass at ca. 90°C. The mass change was 10.55% during heating; thus, the total relative mass change was 12.72%.

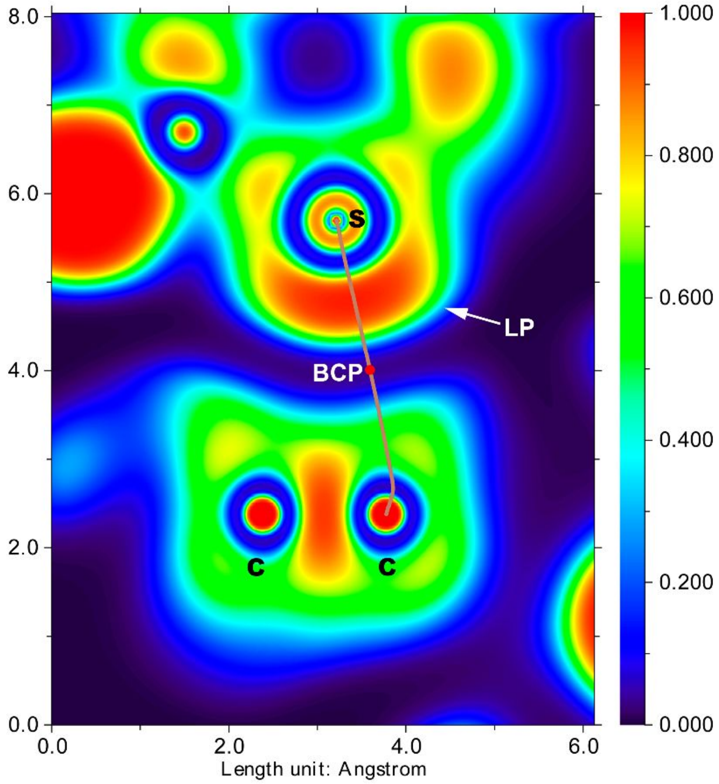

Figure S38: Detail of the 2D ELF plot of compound **3** showing the location of the LP and the bond path connecting the S and C atoms that crosses the LP. The 2D map is defined by the two CC atoms closer to the S atom and the S-atom. Level of theory: PBE0-D4/def2-TZVP.

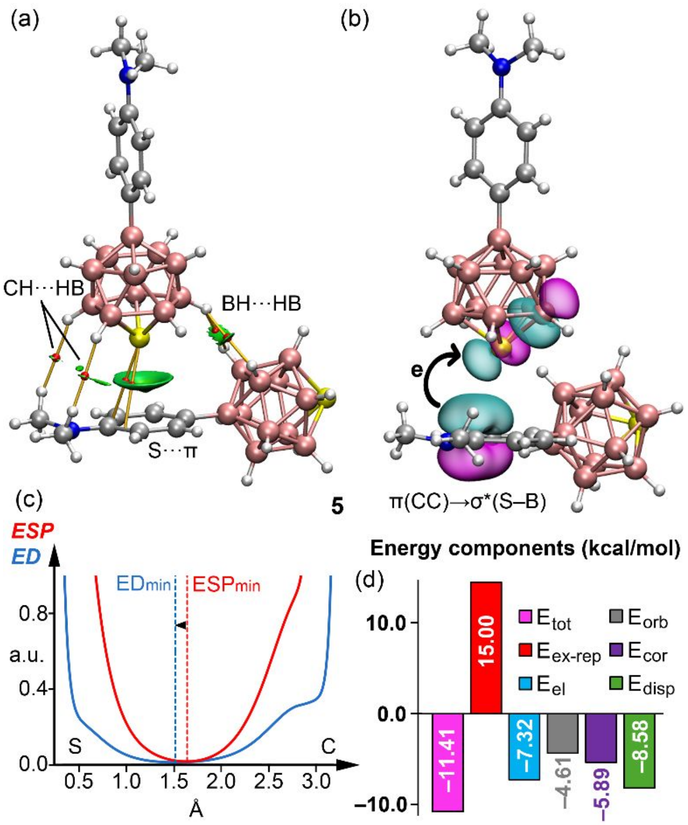

Figure S39: (a) QTAIM distribution of BCPs (red spheres) and bond paths (orange lines) and overlayed NCIplot RDG isosurfaces (ρ cut-off = 0.04 a.u., RDG = 0.5, scale ±0.035 a.u.). (b) Plot of the NBOs involved in the π(CC)→σ*(S–B) charge transfer, including the concomitant stabilization energy. (c) ED vs ESP plot along the path connecting the S to the C-atom. (d) Bar plot of the EDA analysis of the dimer of **5**. Level of theory: PBE0-D4/def2-TZVP.

References

1. Macháček, J.; Plešek, J.; Holub, J.; Hnyk, D.; Všetečka, V.; Císařová, I.; Kaupp, M.; Štíbr, B. New route to 1-thia-*closo*-dodecaborane(11), *closo*-1-$SB_{11}H_{11}$, and its halogenation reactions. The effect of the halogen on the dipole moments and the NMR spectra and the importance of spin–orbit coupling for the ^{11}B chemical shifts. *Dalton Trans.* **2006**, *8*, 1024-1029. DOI: 10.1039/B512345C
2. Sheldrick, G. M. *SHELXT* - Integrated space-group and crystal-structure determination. *Acta Cryst.* **2015**, *A71*, 3–8. DOI: 10.1107/S2053273314026370
3. APEX3 v2016.9-0. *Bruker AXS Inc.* **2016**.
4. Frisch, M. J.; Trucks, G.W.; Schlegel, H. B.; Scuseria, G.E.; Robb, M.A.; Cheeseman, J. R.; Scalmani, G.; Barone, V.; Mennucci, B.; Petersson, G.A.; Nakatsuji, H.; Caricato, M.; Li, X.; Hratchian, H. P.; Izmaylov, A. F.; Bloino, J.; Zheng, G.; Sonnenberg, J. L.; Hada, M.; Ehara, M.; Toyota, K.; Fukuda, R.; Hasegawa, J.; Ishida, M.; Nakajima, T.; Honda, Y.; Kitao, O.; Nakai,H.; Vreven, T.; Montgomery, J. A.; Peralta, Jr., J. E.; Ogliaro, F.; Bearpark, M.; Heyd, J. J.; Brothers, E.; Kudin, K. N.; Staroverov, V. N.; Kobayashi, R.; Normand, J.; Raghavachari, K.; Rendell, A.; Burant, J. C.; Iyengar,S. S.; Tomasi, J.; Cossi, M.; Rega, N.; Millam, J. M.; Klene, M.; Knox, J. E.; Cross, J. B.; Bakken, V.; Adamo, C.; Jaramillo, J.; Gomperts, R.; Stratmann, R. E.; Yazyev, O.; Austin, A. J.; Cammi, R.; Pomelli, C.; Ochterski, J. W.; Martin, R. L.; Morokuma, K.; Zakrzewski, V. G.; Voth, G. A.; Salvador, P.; Dannenberg, J. J.; Dapprich, S.; Daniels, A. D.; Farkas, O.; Foresman, J. B.; Ortiz, J. V.; Cioslowski, J,; D. J. Fox D. J. Gaussian 09, Revision, D.01. *Gaussian, Inc.* **2009**.
5. Flűkiger, P.; Lűthi, H. P.; Portmann, S.; Weber, J. MOLEKEL 4.3. *Swiss Center for Scientific Computing* **2000**.
6. Portmann, S.; Lűthi, H. P. MOLEKEL: An Interactive Molecular Graphics Tool. *Chimia* **2000**, *54*, 766–770. DOI: 10.2533/chimia.2000.766
7. Jeziorski, B.; Moszynski, R.; Szalewicz, K. Perturbation Theory Approach to Intermolecular Potential Energy Surfaces of van der Waals Complexes. *Chem. Rev.* **1994**, *94*, 1887–1930. DOI: 10.1021/cr00031a008
8. Parker, T. M.; Burns, L. A.; Parrish, R. M.; Ryno, A. G.; Sherrill, C. D. Levels of symmetry adapted perturbation theory (SAPT). I. Efficiency and performance for interaction energies. *J. Chem. Phys.* **2014**, *140* (9), 094106. DOI: 10.1063/1.4867135
9. Hostaš, J.; Řezáč, J. Accurate DFT-D3 Calculations in a Small Basis Set. *J. Chem. Theory Comput.* **2017**, *13* (8), 3575–3585. DOI: 10.1021/acs.jctc.7b00365
10. Ahlrichs, R.; Bar, M.; Haser, M.; Horn, H.; Kolmel, C. Electronic structure calculations on workstation computers: The program system Turbomole. *Chem. Phys. Lett.* **1989**, *162* (3), 165–169. DOI: 10.1016/0009-2614(89)85118-8
11. Turney, J. M.; Simmonett, A. C.; Parrish, R. M.; Hohenstein, E. G.; Evangelista, F. A.; Ferman, J. T.; Mintz, B. J.; Burns, L. A.; Wilke, J. J.; Abrams, M. L.; Russ, N. J.; Leininger, M. L.; Janssen, C. L.; Seidl, E. T.; Allen, W. D.; Schaefer, H. F.; King, R. A.; Valeev, E. F.; Sherrill, C. D.; Crawford, T.D. Psi4: an open-source ab initio electronic structure program. *WIREs Comput. Mol. Sci.* **2012**, *2* (4), 556–565. DOI: 10.1002/wcms.93
12. Řezáč, J. Cuby: An integrative framework for computational chemistry. *J. Comput. Chem.* **2016**, *37*, 1230–1237. DOI: DOI: 10.1002/jcc.24312
13. Case, D. A.; Babin, V.; Berryman, J. T.; Betz, R. M.; Cai, Q.; Cerutti, D. S.; Cheatham, T.; Darden, T.; Duke, R. E.; Gohlke, H.; Goetz, A. W.; Gusarov, S.; Homeyer, N.; Janowski, P.;

Kaus, J.; Kolossváry, I.; Kovalenko, A.; Lee, T. S.; LeGrand, S.; Luchko, T.; Luo, R.; Madej, B.; Merz, K. M.; Paesani, F.; Roe, D. R.; Roitberg, A.; Sagui, C.; Salomon-Ferrer, R.; Seabra, G.; Simmerling,C. L. Smith, W.; Swails, J.; Walker, R. C.; Wang, J.; Wolf, R. M.; Wu, X.; Kollman P. A. Amber **2014** (University of California).

14. Pettersen, E. F.; Goddard, T. D.; Huang, C. C.; Couch, G. S.; Greenblatt, D. M.; Meng, E. C.; Ferrin, T. E. UCSF Chimera-a visualization system for exploratory research and analysis. *J. Comput. Chem.* **2004**, *25* (13), 1605–1612. DOI: 10.1002/jcc.20084
15. Wang, J.; Wolf, R. M.; Caldwell, J. W.; Kollman, P. A.; Case, D. A. Development and testing of a general amber force field. *J. Comput. Chem.* **2004**, *25* (9), 1157–1174. DOI: 10.1002/jcc.20035
16. Fanfrlík, J.; Pecina, A.; Řezáč, J.; Lepšík, M.; Sárosi, M. B.; Hnyk, D.; Hobza, P. Benchmark Data Sets of Boron Cluster Dihydrogen Bonding for the Validation of Approximate Computational Methods. *ChemPhysChem.* **2020**, *21* (23), 2599–2604. DOI: 10.1002/cphc.202000729
17. Bader, R. F. W. A quantum theory of molecular structure and its applications, *Chem. Rev.* **1991**, *91* (5), 893-928. DOI: 10.1021/cr00005a013
18. Kitaura, K.; Morokuma, K. A new energy decomposition scheme for molecular interactions within the Hartree-Fock approximation, *Int. J. Quantum Chem.* **1976**, *10*, 325-340. DOI: 10.1002/qua.560100211
19. Becke, A. D.; Edgecombe, K. E. A simple measure of electron localization in atomic and molecular systems. *J. Chem. Phys.* **1990**, *92*, 5397–5403. DOI: 10.1063/1.458517
20. Contreras-García, J.; Johnson, E. R.; Keinan, S.; Chaudret, R.; Piquemal, J.-P.; Beratan, D. N.; Yang, W. NCIPLOT: A Program for Plotting Noncovalent Interaction Regions, *J. Chem. Theory Comput.* **2011**, *7* (3), 625-632. DOI: 10.1021/ct100641a
21. Adamo, C.; Barone, V. Toward reliable density functional methods without adjustable parameters: The PBE0 model. *J. Chem. Phys.* **1999**, *110*, 6158-6170. DOI: 10.1063/1.478522
22. Caldeweyher, E.; Ehlert, S.; Hansen, A.; Neugebauer, H.; Spicher, S.; Bannwarth, C.; Grimme, S. A generally applicable atomic-charge dependent London dispersion correction. *J. Chem. Phys.* **2019**, *150*, 154122. DOI: 10.1063/1.5090222
23. Weigend, F. Accurate Coulomb-fitting basis sets for H to Rn. *Phys. Chem. Chem. Phys.* **2006**, *8* (9), 1057-1065. DOI: 10.1039/B515623H
24. Weigend, F.; Ahlrichs, R. Balanced basis sets of split valence, triple zeta valence and quadruple zeta valence quality for H to Rn: Design and assessment of accuracy. *Phys. Chem. Chem. Phys.* **2005**, *7* (18), 3297-3305. DOI: 10.1039/B508541A
25. Gomila, R. M.; Frontera, A.; Tiekink, E. R. T. Te⋯I secondary-bonding interactions in crystals containing tellurium(II), tellurium(IV) and iodide atoms: supramolecular aggregation patterns, nature of the non-covalent interactions and energy considerations. *Cryst. Eng. Comm.* **2024**, *26* (21), 2784-2795. DOI: 10.1039/D4CE00305E
26. Beccaria, R.; Dhaka, A.; Calabrese, M.; Pizzi, A.; Frontera, A.; Resnati, G. Chalcogen and Hydrogen Bond Team up in Driving Anion⋯ Anion Self-Assembly. *Chem. Eur. J.* **2024**, *30*, e202303641. DOI: 10.1002/chem.202303641
27. Humphrey, W.; Dalke, A.; Schulten, K. VMD: Visual molecular dynamics, *J. Mol. Graph.* **1996**, *14*, 33-38. DOI: 10.1016/0263-7855(96)00018-5
28. Lu, T; Chen, F. Multiwfn: A multifunctional wavefunction analyzer, *J. Comput. Chem.* **2012**, *33*, 580-592. DOI: 10.1002/jcc.22885

29. Glendening, E. D.; Landis, C. R.; Weinhold, F.; Natural bond orbital methods, *WIREs Comput. Mol. Sci.* **2012**, *2*, 1-42. DOI: 10.1002/wcms.51
30. Glendening, E. D.; Badenhoop, J. K.; Reed, A. E.; Carpenter, J. E.; Bohmann, J. A.; Morales, C. M.; Karafiloglou, P.; Landis, C. R.; Weinhold, F., NBO 7.0., **2018**.
31. Spek, A. L. PLATON SQUEEZE: a tool for the calculation of the disordered solvent contribution to the calculated structure factors. *Acta Cryst.* **2015**, *C71*, 9–18. DOI: 10.1107/S2053229614024929
32. Macrae, C. F.; Sovago, I.; Cottrell, S. J.; Galek, P. T. A.; McCabe, P.; Pidcock, E.; Platings, M.; Shields, G. P.; Stevens, J. S.; Towler, M.; Wood, P. A. Mercury 4.0: from visualization to analysis, design and prediction. *J. Appl. Cryst.* **2020**, *53*, 226–235. DOI: 10.1107/S1600576719014092
33. Toth, J. Uniform Interpretation of Gas/Solid Adsorption*. Adv. Colloid Interface Sci.* **1995**, *55*, 1–239. DOI: 10.1016/0001-8686(94)00226-3
34. Myers, A. L.; Prausnitz, J. M. Thermodynamics of Mixed-Gas Adsorption. *AIChE J.* **1965**, *11*, 121–127. DOI: 10.1002/aic.690110125
35. Do, D. D. Adsorption Analysis: Equilibria and Kinetics. *Imperial College Press* **1998**. DOI: 10.1142/p111
36. Reid, C. R.; Thomas, K. M. Adsorption Kinetics and Size Exclusion Properties of Probe Molecules for the Selective Porosity in a Carbon Molecular Sieve Used for Air Separation. *J. Phys. Chem. B* **2001**, *105* (43), 10619–10629. DOI: 10.1021/jp0108263
37. Yang, Q.; Zhong, C. Electrostatic-Field-Induced Enhancement of Gas Mixture Separation in Metal-Organic Frameworks: A Computational Study. *ChemPhysChem.* **2006**, *7*, 1417–1421. DOI: 10.1002/cphc.200600191
38. Yang, Q.; Zhong, C. Molecular Simulation of Carbon Dioxide/Methane/Hydrogen Mixture Adsorption in Metal−Organic Frameworks. *J. Phys. Chem. B* **2006**, *110* (36), 17776–17783. DOI: 10.1021/jp062723w
39. Mu, B.; Li, F.; Walton, K. S. A novel metal–organic coordination polymer for selective adsorption of CO_2 over CH_4. *Chem. Commun.* **2009**, 2493–2495. DOI: 10.1039/B819828D
40. Babarao, R.; Hu, Z.; Jiang, J.; Chempath, S.; Sandler, S. I. Storage and Separation of CO_2 and CH_4 in Silicalite, C_{168} Schwarzite, and IRMOF-1: A Comparative Study from Monte Carlo Simulation. *Langmuir* **2007**, *23* (2), 659–666. DOI: 10.1021/la062289p
41. Liu, B.; Yao, S.; Liu, X.; Li, X.; Krishna, R.; Li, G.; Huo, Q.; Liu, Y. Two Analogous Polyhedron-Based MOFs with High Density of Lewis Basic Sites and Open Metal Sites: Significant CO_2 Capture and Gas Selectivity Performance. *ACS Appl. Mater. Interfaces* **2017**, *9* (38), 32820–32828. DOI: 10.1021/acsami.7b10795
42. Zhao, J.; Xie, K.; Singh, K.; Xiao, G.; Gu, Q.; Zhao, Q.; Li, G.; Xiao, P.; Webley, P. A. Li^+/ZSM-25 Zeolite as a CO_2 Capture Adsorbent with High Selectivity and Improved Adsorption Kinetics, Showing CO_2-Induced Framework Expansion. *J. Phys. Chem. C* **2018**, *122* (33), 18933–18941. DOI: 10.1021/acs.jpcc.8b04152
43. Policicchio, A.; Zhao, Y.; Zhong, Q.; Agostino, R. G.; Bandosz, T. J. Cu-BTC/Aminated Graphite Oxide Composites As High-Efficiency CO_2 Capture Media. *ACS Appl. Mater. Interfaces* **2014**, *6* (1), 101–108. DOI: 10.1021/am404952z
44. Chen, Y.; Lv, D.; Wu, J.; Xiao, J.; Xi, H.; Xia, Q.; Li, Z. A new MOF-505@GO composite with high selectivity for CO_2/CH_4 and CO_2/N_2 separation. *Chem. Eng. J.* **2017**, *308*, 1065–1072. DOI: 10.1016/j.cej.2016.09.138

45. Zhou, X.; Huang, W.; Miao, J.; Xia, Q.; Zhang, Z.; Wang, H.; Li, Z. Enhanced separation performance of a novel composite material GrO@MIL-101 for CO_2/CH_4 binary mixture. *Chem. Eng. J.* **2015**, *266*, 339–344. DOI: 10.1016/j.cej.2014.12.021
46. Huang, W.; Zhou, X.; Xia, Q.; Peng, J.; Wang, H.; Li, Z. Preparation and Adsorption Performance of GrO@Cu-BTC for Separation of CO_2/CH_4. *Ind. Eng. Chem. Res.* **2014**, *53* (27), 11176–11184. DOI: 10.1021/ie501040s
47. Banerjee, R.; Furukawa, H.; Britt, D.; Knobler, C.; O'Keeffe, M.; Yaghi, O. M. Control of Pore Size and Functionality in Isoreticular Zeolitic Imidazolate Frameworks and their Carbon Dioxide Selective Capture Properties. *J. Am. Chem. Soc.* **2009**, *131* (11), 3875–3877. DOI: 10.1021/ja809459e
48. Si, X.; Jiao, C.; Li. F.; Zhang, J.; Wang, S.; Liu, S.; Li, Z.; Sun, L.; Xu, F.; Gabelica, Z.; Schick, C. High and selective CO_2 uptake, H_2 storage and methanol sensing on the amine-decorated 12-connected MOF CAU-1. *Energ. Environ. Sci.* **2011**, *4* (11), 4522–4527. DOI: 10.1039/C1EE01380G
49. Finsy, V.; Ma, L.; Alaerts, L.; De Vos, D.; Baron, G.; Denayer, J. F. M. Separation of CO2/CH4 mixtures with the MIL-53(Al) metal–organic framework. *Micropor. Mesopor. Mater.* **2009**, *120* (3), 221–227. DOI: 10.1016/j.micromeso.2008.11.007
50. Delgado, J. A.; Uguina, M. A.; Gomez, J. M.; Ortega, L. Adsorption equilibrium of carbon dioxide, methane and nitrogen onto Na- and H-mordenite at high pressures. *Sep. Purif. Technol.* **2006**, *48* (3), 223–228. DOI: 10.1016/j.seppur.2005.07.027
51. Xu, X.; Zhao, X.; Sun, L.; Liu, X. Adsorption separation of carbon dioxide, methane, and nitrogen on Hβ and Na-exchanged β-zeolite. *J. Nat. Gas Chem.* **2008**, *17* (4), 391–396. DOI: 10.1016/S1003-9953(09)60015-3
52. Salehi, S.; Anbia, M. High CO_2 Adsorption Capacity and CO_2/CH_4 Selectivity by Nanocomposites of MOF-199. *Energy Fuels* **2017**, *31* (5), 5376–5384. DOI: 10.1021/acs.energyfuels.6b03347
53. Palomino, M.; Corma, A.; Rey, F.; Valencia, S. New Insights on CO_2−Methane Separation Using LTA Zeolites with Different Si/Al Ratios and a First Comparison with MOFs. *Langmuir* **2010**, *26* (3), 1910–1917. DOI: 10.1021/la9026656
54. Mohan, M.; Essalhi, M.; Durette, D.; Rana, L. K.; Ayevide, F. K.; Maris, T.; Duong, A. A Rational Design of Microporous Nitrogen-Rich Lanthanide Metal–Organic Frameworks for CO_2/CH_4 Separation. *ACS Appl. Mater. Interfaces* **2020**, *12* (45), 50619–50627. DOI: 10.1021/acsami.0c15395
55. Yan, X.; Komarneni, S.; Zhang, Z.; Yan, Z. Extremely enhanced CO_2 uptake by HKUST-1 metal–organic framework via a simple chemical treatment. *Micropor. Mesopor. Mater.* **2014**, *183*, 69–73. DOI: 10.1016/j.micromeso.2013.09.009
